# Carbene-Controlled Regioselective Functionalization of Linear Alkanes under Silver Catalysis

**DOI:** 10.1021/jacs.2c11707

**Published:** 2022-12-13

**Authors:** María Álvarez, Francisco Molina, Pedro J. Pérez

**Affiliations:** Laboratorio de Catálisis Homogénea, Unidad Asociada al CSIC, CIQSO-Centro de Investigación en Química Sostenible and Departamento de Química, Universidad de Huelva, 21007 Huelva, Spain

## Abstract

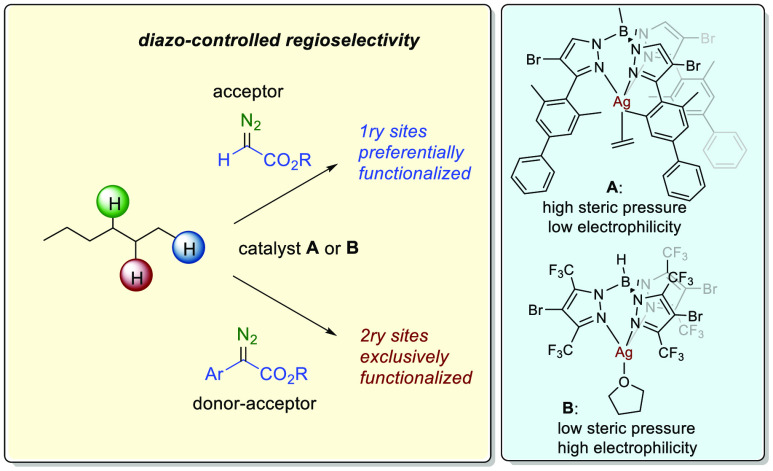

Control of the regioselectivity in the functionalization of C–H bonds of linear alkanes C_2_H_2n+2_ via carbene transfer from diazo compounds is restricted to the use of rhodium-based catalysts, which govern the reaction outcome employing donor–acceptor diazo reagents. At variance with that catalyst-controlled strategy, we present an alternative approach in which employing the appropriate silver complexes containing trispyrazolylborate ligands as catalysts with large differences in their steric and electronic properties, the regioselection is mainly governed by the diazo reagent, which leads to the functionalization of primary or secondary sites of linear alkanes (lacking any activating or directing groups). Donor–acceptor aryl diazoacetates exclusively provide the functionalization of the secondary sites of hexane or pentane, whereas acceptor ethyl diazoacetate leads to an unprecedented level of primary functionalization.

In recent decades, C–H bond functionalization processes have emerged to be one of the most efficient and straightforward synthetic strategies for constructing complex molecules.^1^ However, such development is yet scarce when applied to the unexpensive and available alkanes C_n_H_2n+2_, for which fewer methodologies have been reported for their modification and further conversion into value-added products.^2^ The main drawbacks affecting their reactivity are related to their high bond dissociation energies, the low carbon–hydrogen bond polarity, and the control of the selectivity due to the presence of several potential as well as distinct reaction sites, i.e., the different C–H bonds existing in an alkane molecule.^3^

One of the strategies that has undergone tremendous progress is the metal-catalyzed functionalization of C–H bonds by carbene insertion from a diazo reagent or its precursor (Scheme 1).^4^ At the end of the 90s of the last century, the use of such strategy with alkanes was yet merely a curiosity, with just a few examples known.^5^ However, intensive research has provided a number of catalytic systems, which has allowed the modification of alkane C–H bonds from the simplest one, methane,^6^ to saturated macromolecules exclusively containing C–H bonds.^7^ Our group and Dias’ group developed Cu- and Ag-based catalysts for the effective but unselective functionalization of linear alkanes.^8,9^ However, examples where a high degree of selectivity toward one specific C–H bond in the alkane employed is induced are yet rare. This area is dominated by the ground-breaking research developed by Davies in the past few years (Scheme 1).^10^ With *n*-pentane or *n*-hexane as the model substrates, bearing primary and two distinct secondary C–H bonds, Davies has disclosed rhodium-based catalysts which specifically direct toward one or the other.^10a,10b^ Also, for alkanes also having tertiary C–H bonds, a third catalytic system selective toward that site was also described.^10c^ As a general feature, Davies employs a donor–acceptor carbene from the corresponding diazo compound,^11^ the ligand of the dirhodium catalyst being the crucial variable to direct the carbene transfer toward the desired C–H bond. Apart from rhodium, the silver-based catalyst [Tp^Br3^Ag]_2_, previously reported^12^ by our group for the unselective alkane functionalization using ethyl diazoacetate (acceptor-H carbene) as the carbene source, has been employed by Bi for the selective functionalization of tertiary sites, employing N-triftosylhydrazone as the carbene precursor (donor-H carbene).^13^ No examples of selective alkane primary or secondary C–H functionalization are known with metals distinct from rhodium. It is worth noting that we always refer to alkanes (C_n_H_2n+2_), and not to C_alkyl_–H bonds in molecules also containing activating groups (unsaturated bonds and/or heteroatoms), which usually are more prone to be functionalized since such groups affect the bond dissociation energy and/or the polarity of the pursued bonds. Given the scarcity of selective catalytic systems for alkanes as substrates within this methodology, we targeted the design of silver-based catalysts toward that end, aiming at favoring the yet undescribed selective functionalization of primary or secondary sites. Herein we describe the results of such studies, which have allowed the preferential functionalization of those sites in model alkanes such as pentane or hexane. At variance with the rhodium chemistry, in our case the selectivity is mainly influenced by the carbene substituents since the same catalysts (**A** or **B** in Scheme 1) can direct the functionalization toward different sites.

**Scheme 1:**
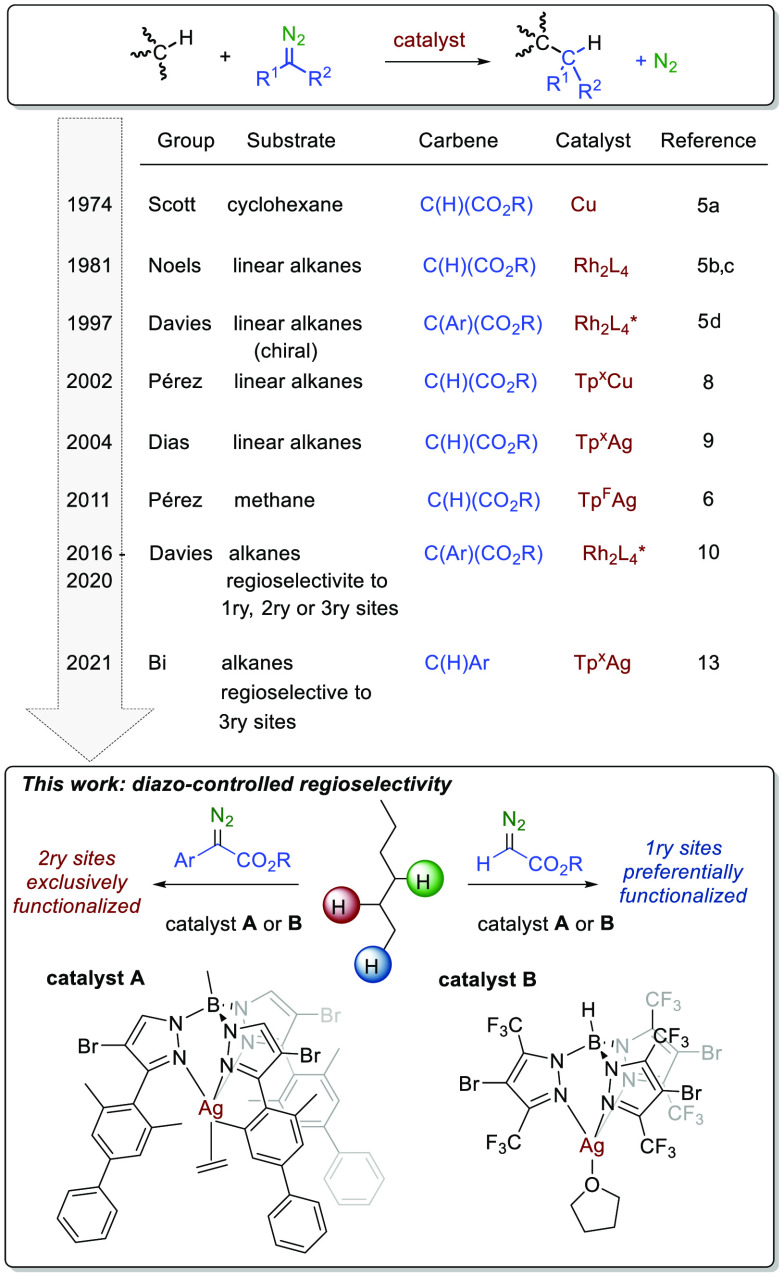
Development of Alkane C–H Bond Functionalization by Carbene Insertion Strategy

Our previous work^6,8,14^ showed that complexes containing the Tp^x^Ag core (Tp^x^ = hydrotrispyrazolylborate ligand) bearing electron withdrawing groups at Tp^x^ ligand were quite active for the functionalization of alkanes, albeit in an unselective manner, since all possible C–H bonds in the alkanes were functionalized. To induce regioselection, an appropriate balance between electronic and steric effects of the three main actors (Figure 1) in this transformation (catalyst, carbene and alkane) is needed. Electrophilicity at the metal center needs to be high enough that the silver-carbene can react with the poor nucleophiles, alkane C–H bonds. However, a strong electrophile would not discriminate between different sites. In addition to the Tp^x^ ligand, the groups of the carbenic carbon can be also varied in the search for such balance. Steric effects require adjustment as well, and this can be done modifying the R^1^ groups at Tp^x^ ligand and/or the substituents at the carbenic carbon. The nature of the alkane C–H bond is crucial given the difference in nucleophilicity and sterics between primary and secondary C–H bonds for linear alkanes.^14a^

**Figure 1:**
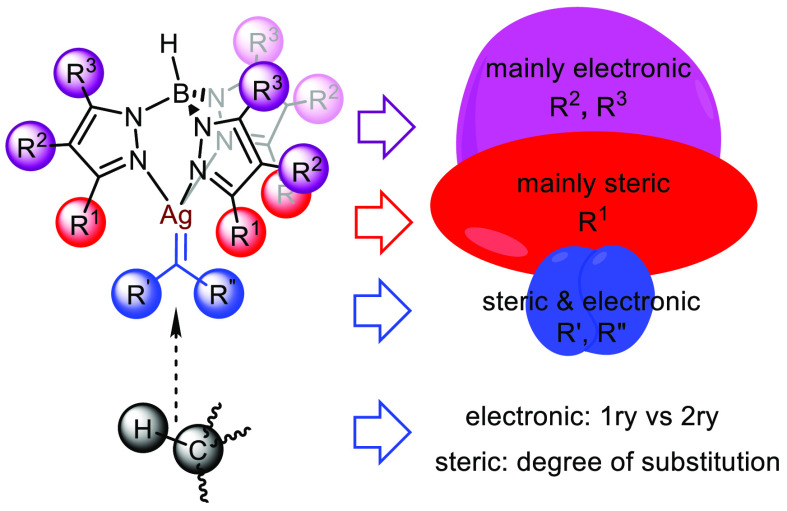
Effect of the different actors (catalyst, diazo, and alkane) in the C–H bond functionalization by carbene insertion strategy.

With all these data in hand, we designed a study in which those variables are evaluated, employing four silver catalysts with different Tp^x^ ligands (Scheme 2), four types of diazo reagents, and linear alkanes C5–C6 as the model substrates. The silver complexes [Tp^Br3^Ag]_2_ (**1**) and Tp^(CF3)2,Br^Ag(THF) (**2**) have been previously described,^12,14b^ whereas those bearing the biphenyl-containing ligands Me-Tp^Me2-biphen^Ag(C_2_H_4_) (**3**) and Me-Tp^Me2-biphen,4Br^Ag(C_2_H_4_) (**4**) have been synthesized for this work. The preparation and characterization procedures of the new ligand Me-Tp^Me2-biphen,4Br^ and complexes **3** and **4** are given in the Supporting Information.

**Scheme 2:**
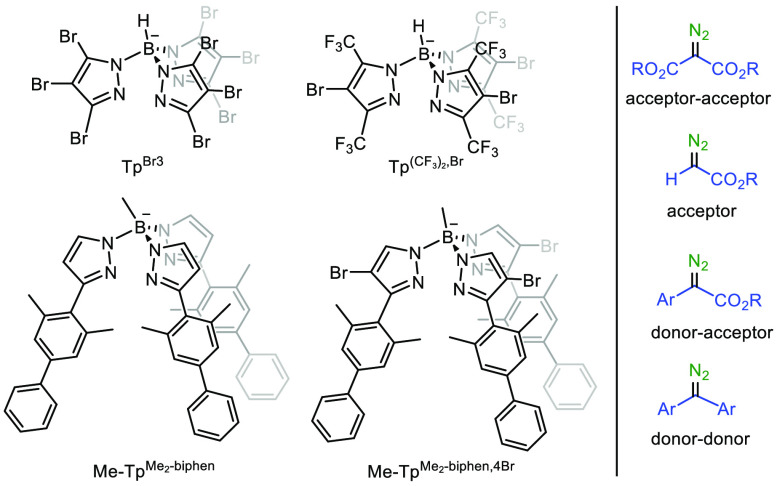
Ligands and Diazo Compounds Employed in This Work

We first employed ethyl diazoacetate (EDA) as the carbene source to evaluate the functionalization of *n*-hexane with the four catalysts **1**–**4**. Table 1 contains the results of this set of experiments; in all cases, a mixture of the three possible products originated from the insertion of the carbene unit into the three different C–H bonds of *n*-hexane was obtained. Nearly quantitative yields (diazo-based) were achieved with catalysts **2** and **4**. Catalysts **3** and **4**, bearing the bulky substituent at the 3-position of the pyrazolyl ring, differed in the regioselectivity since **4** led to 49% of the C1 functionalization compound, whereas **3** gave 37% of the same compound. This difference is induced by the presence of the bromine in the 4-position and should be considered mainly as an electronic effect.

**Table 1:**
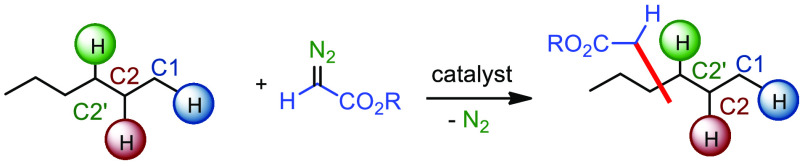

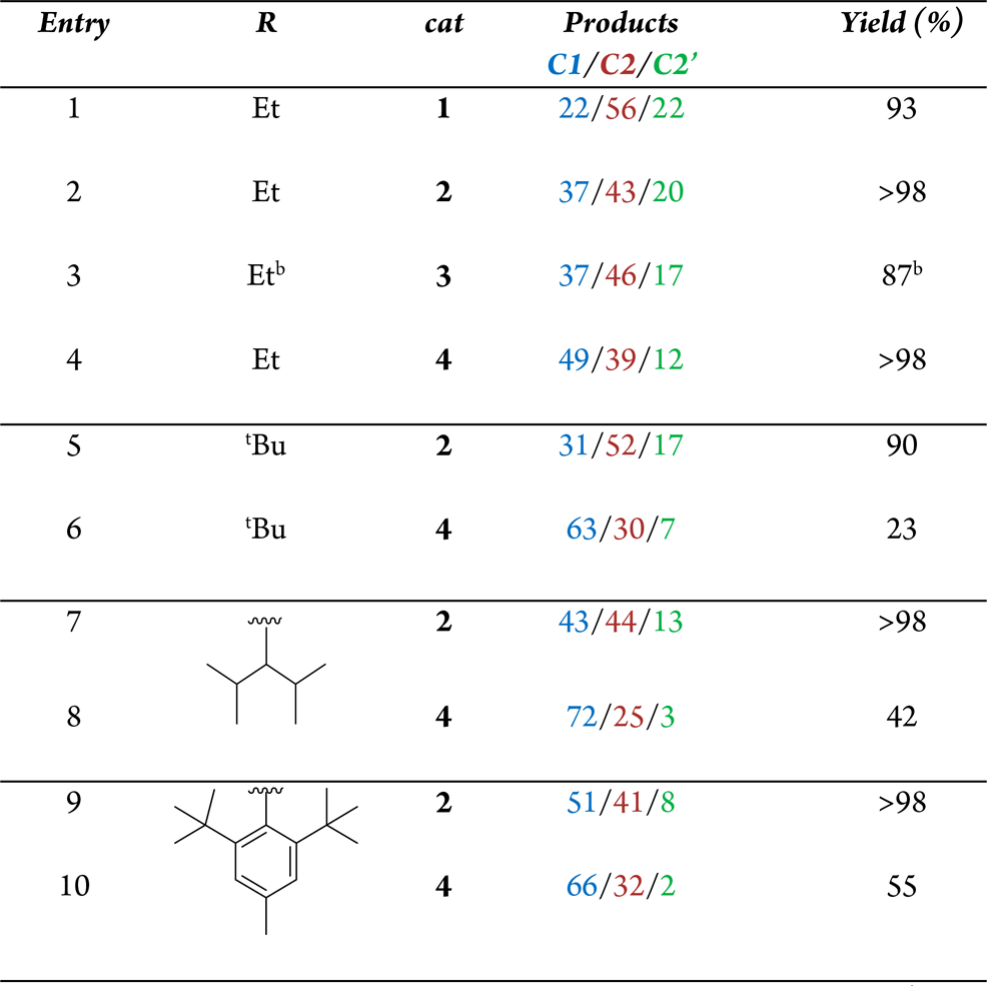
Hexane Functionalization by Carbene Insertion Employing Monosubstituted Acceptor Diazoacetates^a^

a Reaction conditions: 0.15 mmol diazocompound and 6.7 mol % catalyst in hexane (50 mL) at rt for 24 h. Selectivity and yields were determined using GC calibration curves and by ^1^H NMR of the reaction crude (internal standard: trimethoxybenzene).

b At 70 °C.

Aimed at improving the regioselectivity toward the primary C–H bond of *n*-hexane, the variation of the bulkiness of the carbene moiety was investigated. Thus, the parent Et group in EDA was replaced by tBu, 2,4-dimethyl-pent-3-yl, and 2,6-(t-butyl)-4-methylphenyl groups (entries 5–10). We chose catalysts **2** and **4** as representatives of two different environments around the metal centers. The former is a highly electrophilic silver with not much steric pressure induced by the pyrazolyl substituents. On the contrary, catalyst **4** is less electrophilic, but the biphenyl substituents at the pyrazolyl rings greatly affect the catalytic pocket. With the three diazo compounds employed, catalyst **4** gave higher amounts of the product derived from the insertion of the carbene into the terminal C–H bond of hexane. The best result was achieved using the 2,4-dimethyl-pent-3-yl derivative, which gave 72% of such product (entry 8), the highest selectivity known to date toward that site with a linear alkane using the acceptor EDA as the carbene source. Interestingly, only a residual 3% is obtained from the insertion into the C2′–H bond, indicating the relevant steric effect exerted by catalyst **4** and this diazo reagent.

The outstanding selectivity obtained by Davies with rhodium catalysts was achieved with donor–acceptor aryl diazoacetates.^10^ We have now screened the catalytic properties of complexes **2** and **4** toward that end, with Table 2 containing the outcome of this study, which has been done with both *n*-hexane and *n*-pentane. From the array of experiments, several trends can be extrapolated. First, catalyst **2** is more reactive than **4** in terms of yields into functionalized alkanes, and it requires milder conditions, consuming all the diazo reagent in 15 min at room temperature. The bulkier, less electrophilic catalyst **4** needed heating for 3 h to achieve complete conversion.

**Table 2:**
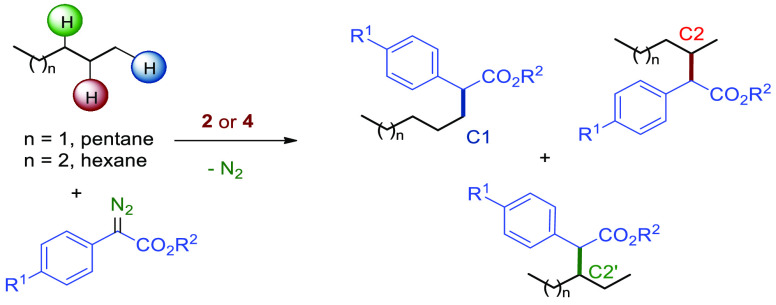
Selective Functionalization of Secondary Sites of Pentane and Hexane^a^

	[Ag]	n	R^1^	R^2^	1ry:2ry ratio	C2:C2′ ratio	yield (%)
1	**4**	2^b^	H	Et	2:98	4.5:1.0	30
2	**2**	2^c^	H	Et	2:98	6.0:1.0	79
3	**4**	2^b^	Br	Me	2:98	4.2:1.0	42
4	**4**	1^d^	Br	Me	2:98	5.5:1.0	43
5	**2**	2^c^	Br	Me	2:98	6.0:1.0	51
6	**2**	1^c^	Br	Me	1:99	6.1:1.0	50
7	**4**	2^b^	Br	CH_2_CF_3_	<1^e^:>99	6.1:1.0	51
8	**4**	1^d^	Br	CH_2_CF_3_	2:98	7.2:1.0	49
9	**2**	2^c^	Br	CH_2_CF_3_	<1^e^:>99	6.1:1.0	>98
10	**2**	1^c^	Br	CH_2_CF_3_	<1^e^:>99	10.0:1.0	75
11	**4**	2^b^	Br	CH_2_CCl_3_	2:98	4.2:1.0	51
12	**4**	1^d^	Br	CH_2_CCl_3_	2:98	4.4:1.0	49
13	**2**	2^c^	Br	CH_2_CCl_3_	1:99	6.6:1.0	>98
14	**2**	1^c^	Br	CH_2_CCl_3_	1:99	10.0:1.0	75
15	**4**	2^b^	Cl	CH_2_CF_3_	1:99	6.1:1.0	51
16	**4**	1^d^	Cl	CH_2_CF_3_	<1^e^:>99	8.9:1.0	55
17	**2**	2^c^	Cl	CH_2_CF_3_	1:99	7.25:1.0	>98
18	**2**	1^c^	Cl	CH_2_CF_3_	<1^e^:>99	10.1:1.0	79
19	**4**	2^b^	CF_3_	CH_2_CF_3_	2:98	5.5:1.0	52
20	**4**	1^d^	CF_3_	CH_2_CF_3_	2:98	7.1:1.0	47
21	**2**	2^c^	CF_3_	CH_2_CF_3_	2:98	6.0:1.0	97
22	**2**	1^c^	CF_3_	CH_2_CF_3_	2:98	9.9:1	86

a Reaction conditions: 0.15 mmol diazocompound and 6.7 mol % catalyst loading in alkane (80 mL). Selectivity and yields were determined using GC calibration curves and by ^1^H NMR of the crude reaction mixture (internal standard: trimethoxybenzene). See SI for full description.

b 70 °C, 3 h.

c Room temperature, 15 min.

d 45 °C, 3 h.

e Products from C1 functionalization not observed.

To our delight, the complete induction of regioselection toward secondary sites has been achieved: independently of the catalyst and conditions employed, the amounts of products derived from the insertion of the carbene into the secondary sites were detected within the 98−99% interval. The observation of the product formed upon insertion into the primary sites was always below 2%. In addition to the effect of the catalyst in the conversions, significant differences are also found in the distribution of products regarding the two secondary sites. This ratio ranges from 4.5:1 to 7.25:1 for hexane and from 5.5:1 to 10.1:1 for pentane. Figure 2 displays the values of the C2:C2′ ratio of products for five diazo compounds (pentane as the alkane). Except for that with R^1^ = Br and R^2^ = Me, for which both catalysts nearly provide the same value, the other four examples show considerable catalyst-induced differences. Catalyst **2** gives nearly the same ratio in the four cases (10:1), whereas catalyst **4** is more sensible to the substituents of the carbene moiety. It is worth mentioning that the previously reported rhodium-based system for the highly selective functionalization of the secondary C–H bonds bond in pentane led to a 29:1 ratio for C2:C2′ sites employing the diazo with R^1^ = Br and R^2^ = CH_2_CCl_3_.^10b^

**Figure 2:**
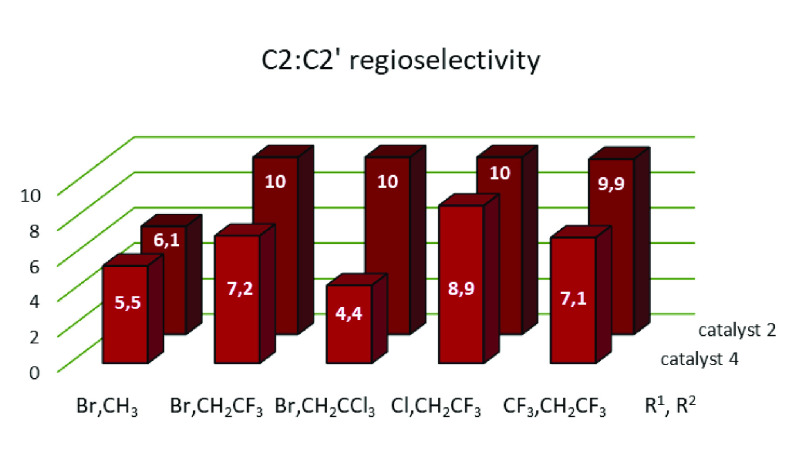
Product ratio C2:C2′ for secondary sites in pentane using catalysts **2** and **4**.

To complete the study on the effect of the nature of the diazo substituents, the use of donor–donor and acceptor–acceptor diazo compounds was also tested with *n*-hexane as the substrate (Scheme 3). When catalysts **2** or **4** were evaluated with diethyl diazomalonate, no reaction was observed at room temperature. Heating at 70 °C resulted in the functionalization of hexane only with the fluorinated catalyst **2**, which led to an unselective mixture of products derived from C1, C2, and C2′ functionalization. The absence of reactivity in this system at room temperature or that of **4** even at high temperature can be explained due to the preference of Tp^x^Ag cores to form a diazo-adduct with this diazomalonate.^15^ The use of diphenyldiazomethane as carbene source representing the donor–donor case led to the olefin formed from silver-catalyzed carbene coupling, and no C–H bond functionalization took place in the alkane.

**Scheme 3:**
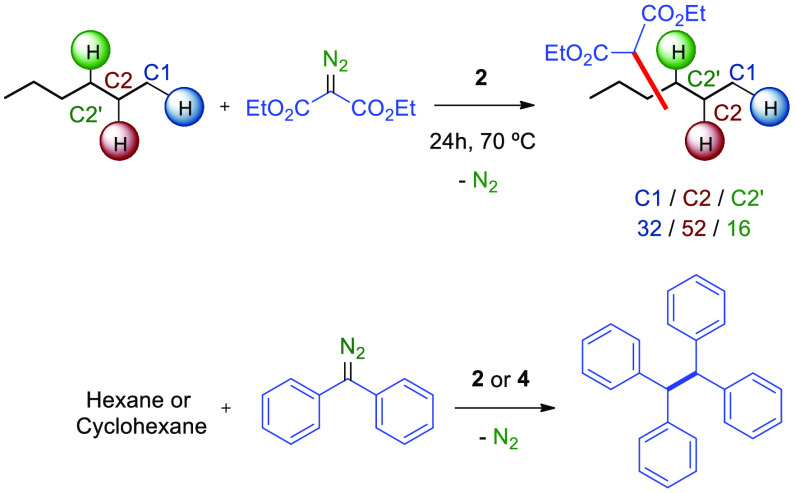
Screening of Acceptor–Acceptor and Donor–Donor Diazo Compounds

The variables that affect the reaction outcome of this catalytic system can be rationalized as follows (Scheme 4). The primary C–H bonds (with a low nucleophilicity compared with the secondary sites)^14a^ can be preferentially functionalized when generating a highly electrophilic silver–carbene intermediate from an acceptor diazo compound N_2_=C(H)CO_2_R. This must be accompanied by a substantial steric effect exerted by a bulky Tp^x^ ligand (catalyst **4**) and a bulky ester substituent (2,4-dimethyl-pent-3-yl), therefore decreasing the reactivity toward the more impeded secondary sites. The use of an acceptor–acceptor diazo compound is not as useful since once the silver–carbene is formed, it is so reactive that no selectivity is induced.

**Scheme 4:**
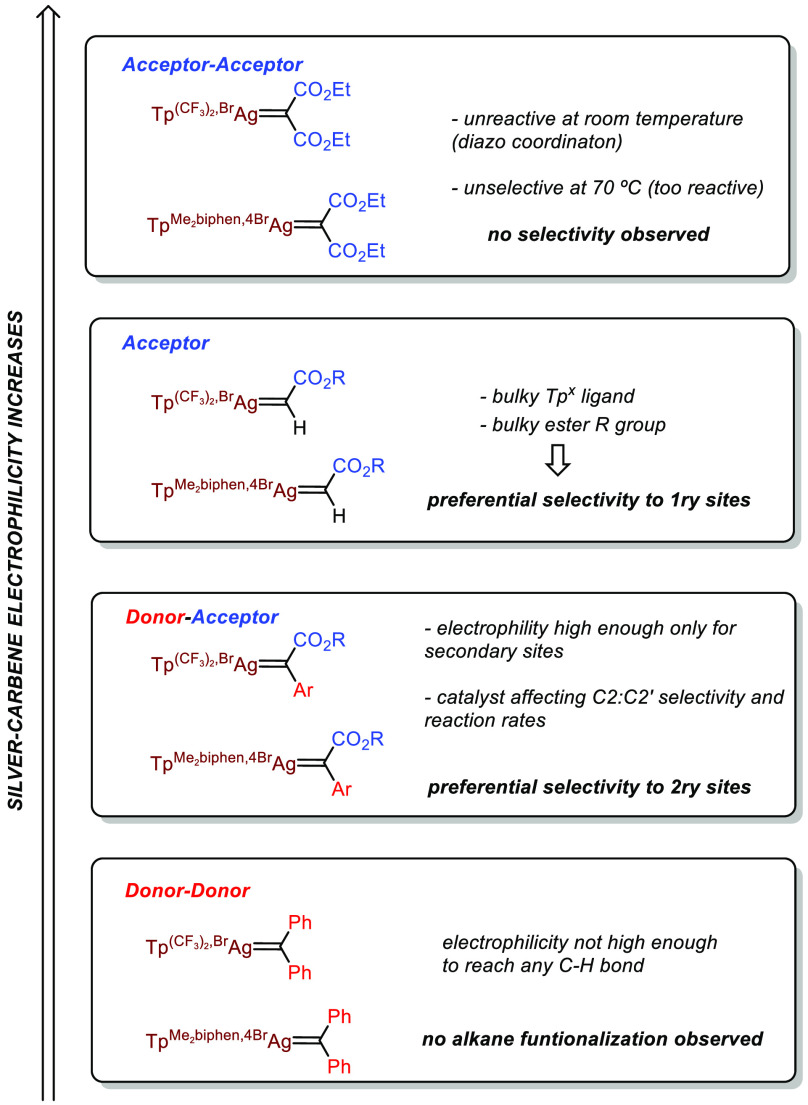
Comparison of the Different Diazo Reagents and Silver Catalysts Employed in This Work

On the contrary, the preferential functionalization of secondary sites requires a less electrophilic silver–carbene intermediate (thus eliminating the reactivity toward primary C–H bonds), which can be achieved by employing donor–acceptor substituents at the parent diazo compound (N_2_=C(Ar)CO_2_R). In this case, the effect of the Tp^x^ ligands employed in this work is only observed in the reaction rate (catalyst **2** operates at room temperature, whereas catalyst **4** requires 45–70 °C) and in the C2:C2′ selectivity (which is lower for catalyst **4**). When moving to a donor–donor diazo compound, the resulting silver–carbene intermediate does not display enough electrophilicity to attack the alkane C–H bonds.

In conclusion, we have found that it is possible to exert regioselection in the silver-catalyzed functionalization of linear alkanes, using pentane or hexane as model substrates, by carbene insertion from diazo compounds. The nature of the diazo compound seems crucial for the control of the regioselectivity: donor–acceptor carbenes direct the reaction to the secondary sites, whereas acceptor carbenes provide preferential insertion in the terminal C–H bonds, the latter enhanced with the use of a bulky Tp^x^ ligand. These results are in contrast with the rhodium chemistry, where the same diazo reagent required distinct catalysts for the selective functionalization of different sites. We believe that these finding will bring new and more selective catalytic systems for the always challenging functionalization of alkanes in a selective manner.

## Supporting Information Available

All procedures and characterization data for new compounds, catalytic experiments, and X-ray diffraction studies (PDF)
