# Supplementary material for: Carbene-Controlled Regioselective Functionalization of Linear Alkanes under Silver Catalysis
Source: J Am Chem Soc. 2022 Dec 13;144(51):23275–9. doi: 10.1021/jacs.2c11707 (PMC9801380; doi:10.1021/jacs.2c11707)
Supplement: Supplementary file 1 — ja2c11707_si_001.pdf [file ja2c11707_si_001.pdf]

Carbene-controlled regioselective functionalization of linear alkanes under silver catalysis

María Álvarez, Francisco Molina and Pedro J. Pérez*

‡ Laboratorio de Catálisis Homogénea, Unidad Asociada al CSIC CIQSO-Centro de Investigación en Química Sostenible and Departamento de Química, Universidad de Huelva, 21007-Huelva (Spain).

Table of Contents

1. General Considerations **S2**
2. Synthesis and characterization of complexes Me-Tp$^{Me2\text{-}biphen}$Ag(C_2H_4) (**3**) y Me-Tp$^{Me2\text{-}biphen,4\text{-}Br}$Ag($C_2H_4$) (**4**). **S2**
3. Synthesis and identification of products generated by catalytic reactions using monosubstituted diazo compounds. **S8**
4. Synthesis and identification of products generated by catalytic reactions using aryl diazo compounds. **S16**
5. Study of the catalytic reactivity of silver complexes with hexane and monosubstituted diazoacetates. **S40**
6. General procedure for the study of reactions with aryl diazoacetates. **S40**
7. Study of the catalytic reactivity of silver complexes with hexane and diethyl diazomalonate. **S40**
8. NMR spectra and GC traces of reaction crudes. **S41**
9. References. **S67**

1. General considerations.

All air and moisture-sensitive manipulations were carried out using standard high vacuum lines and standard Schlenk techniques or inside a glovebox under nitrogen. All reactants were purchased from Sigma-Aldrich or Across and used without further purification. Solvents were dried using a SPS-MBraun system. The compounds 3-(3,5-dimethyl-[1,1'-biphenyl]-4-yl)pyrazole,[1] $[Tp^{Br3}Ag]_2$,[2] $Tp^{(CF3)2,Br)}Ag(THF)$,[3] Me-$Tp^{Me2\text{-}biphen}Tl$[1] and the diazocompounds[4] employed in this work were prepared according to literature procedures. NMR spectra were recorded on Agilent 400 MR or Agilent 500 DD2 spectrometers, ^{1}H and ^{13}C NMR shifts are reported relative to tetramethylsilane. FT-IR spectra were collected on a Nicolet IR200 FTIR spectrometer. Elemental analyses were performed on a Perkin-Elmer Series II CHNS/O Analyzer 2400. Crystal structure determination was carried out using a BRUKER D8 FIXED-CHI diffractometer equipped with an Oxford Cryosystems low-temperature device.

2. Synthesis and characterization of complexes Me-$Tp^{Me2\text{-}biphen}Ag(C_2H_4)$ (3) and Me-$Tp^{Me2\text{-}biphen,4\text{-}Br}Ag(C_2H_4)$ (4).

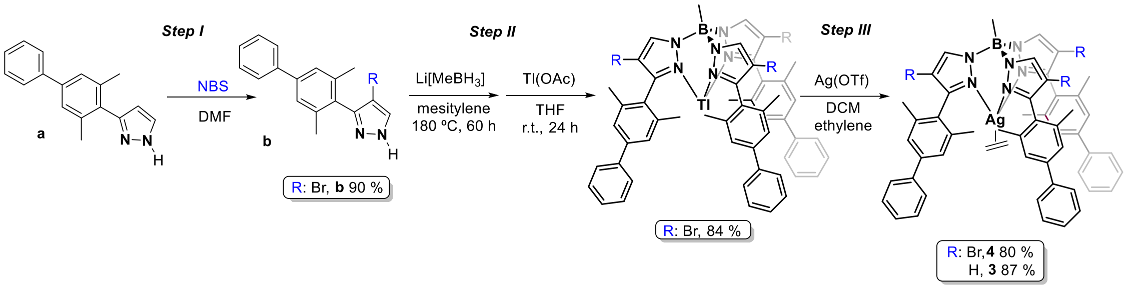

Step I. Synthesis of 3-(3,5-dimethyl-[1,1'-biphenyl]-4-yl)-4-(bromo)-pyrazole (b). The pyrazole **b** was obtained from the bromination of pyrazole 3-(3,5-dimethyl-[1,1'-biphenyl]-4-yl)pyrazole (**a**) in the following manner. In a one-round bottom flask, the pyrazole **a** (1.5 g, 6.05 mmol) was placed before adding 6 mL of DMF. Then N-bromosuccinimide (1.075 g, 6.05 mmol) was added and the mixture was stirred for 1 h. Ethyl acetate (15 mL) was then added and washed with H_2O (8 x 20 mL) to remove all DMF. The organic phase was dried with $MgSO_4$, filtered off, and evaporated leading to the desired pure pyrazole **b** as a beige solid in 90% yield. Analysis calculated for $C_{17}H_{15}BrN_2$ (**b**): C, 62.21; H, 4.91; N, 8.53 %. Found: C, 62.94; H, 4.61; N, 8.65 %. *^{1}H NMR of **b** (400 MHz, $CDCl_3$):* δ 2.50 (s, 6H, CH_3), 7.31 (s, 2H, C$H_{2\text{-}arom}$), 7.36 (m, 1H, CH_{arom}), 7.44 (t, 2H, $J_{H\text{-}H}$ = 8 Hz, CH_{arom}), 7.56 (d, 2H, $J_{H\text{-}H}$ = 8 Hz, CH_{arom}), 7.58 (s, 1H, CH_{pz}). *$^{13}C\{^{1}H\}$ NMR of **b** (100 MHz, $CDCl_3$):* δ 20.1 (CH_3), 95.0 (C_{qpz}), 126.2 (CH_{arom}), 127.1 (CH_{arom}), 127.5 (CH_{arom}), 128.7 (CH_{arom}), 135.9 (CH_{pz}), 140.5 (C_{qarom}), 142.3 (C_{qarom}).

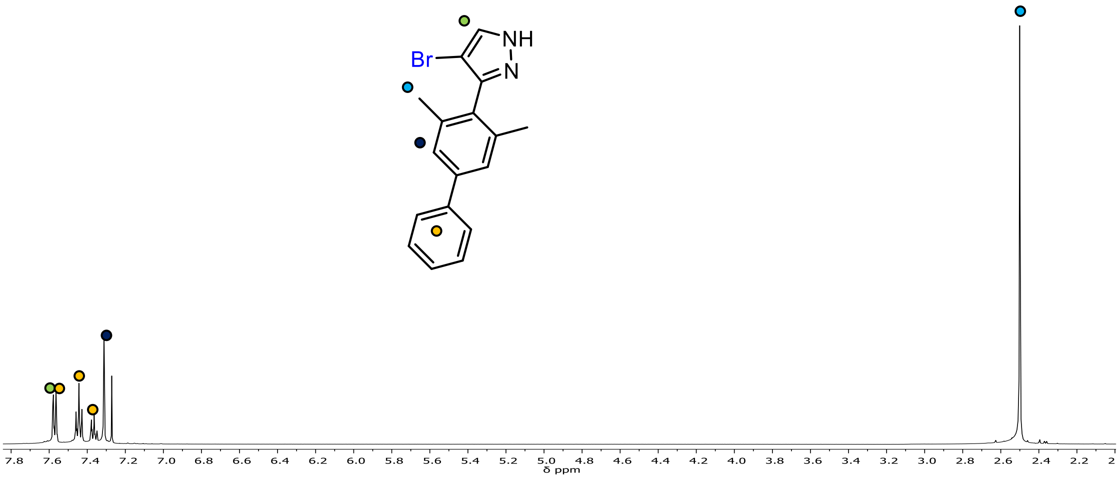

*Figure S1: ^1H NMR spectrum of **b** (400 MHz, $CDCl_3$).*

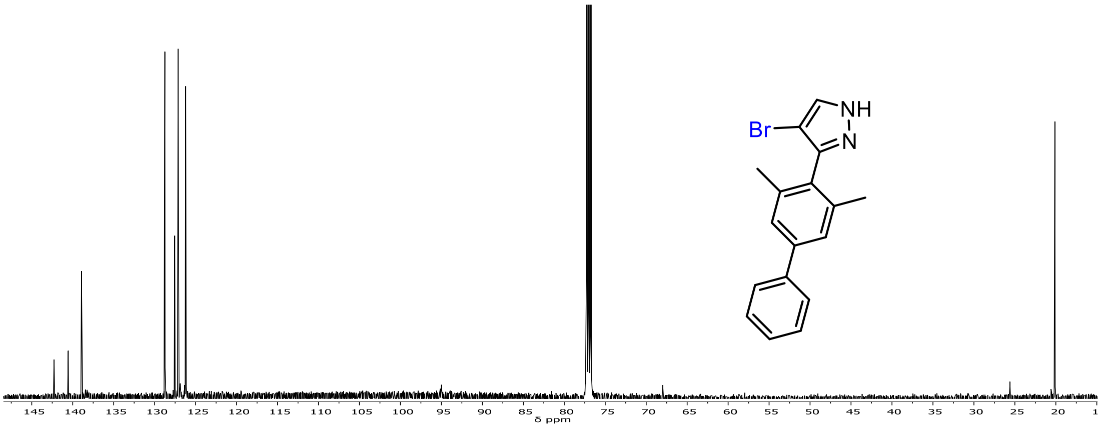

*Figure S2: $^{13}C\{^1H\}$ NMR spectrum of **b** (100 MHz, $CDCl_3$).*

Step II. Synthesis of Tl(Me-Tp$^{Me2\text{-}biphen,4\text{-}Br}$). In a Schlenk tube under nitrogen atmosphere, 3-(3,5-dimethyl-[1,1'-biphenyl]-4-yl)4-(bromo)-pyrazole (1.41 g, 4.32 mmol) and $LiMeBH_3$ (46.82 mg, 1.31 mmol) were placed along with 5 mL of mesitylene, and the mixture was stirred for 5 h at room temperature. The tube was connected to the gas line to release over-pressures due to hydrogen release. After that time, the mixture was heated at 180 °C for 60 h, before being cooled at 50 °C. Volatiles were removed at that temperature under reduced pressure and the residue dissolved in dry THF (10 mL). Tl(OAc) (344.6 mg, 1.31 mmol) was then added and the mixture stirred for 24 h at room temperature. Solid was removed by filtration, volatiles again were removed under reduced pressure and the resulting white solid was washed with MeOH (4 x 30 mL) to remove excess of pyrazole. Tl(Me-Tp$^{Me2\text{-}biphen,4\text{-}Br}$) was obtained as a white solid in 84% yield. Single crystals of the complex were obtained in chloroform and its solid structure determined by X-ray studies. CCDC 2216792 contains the supplementary crystallographic data for this compound. Analysis calculated for $C_{52}H_{45}BrBTlN_2$: C, 51.71; H, 3.67; N, 6.96 %. Found: C, 51.88; H, 4.03; N, 6.65 %. *^1H NMR (400 MHz, $CDCl_3$):* δ 1.12 (s, 3H, BC*H*$_3$), 1.98 (s, 18H, C*H*$_3$), 7.27 (s, 6H, CH_{arom}), 7.29 (m, 3H, CH_{arom}), 7.38 (t, 6H, $J_{H\text{-}H}$ = 8 Hz, CH_{arom}), 7.57 (d, 6H, $J_{H\text{-}H}$ = 8 Hz, CH_{arom}), 7.91 (d, 3H, $J_{H\text{-}Tl}$ = 0.5 Hz, CH_{pz}). *$^{13}C\{^1H\}$ NMR (100 MHz, $CDCl_3$):* δ 19.9 (*C*H$_3$), 94.0 ($C_{q\text{-}pz}$), 125.9

(CH$_{arom}$), 127.0 (CH$_{arom}$), 127.4 (CH$_{arom}$), 128.6 (CH$_{arom}$), 129.6 (C_{qarom}), 134.3 (CH$_{pz}$), 138.3 ($C_{q\text{-}arom}$), 140.5 ($C_{q\text{-}arom}$), 141.3 ($C_{q\text{-}arom}$), 149.7 ($C_{q\text{-}pz}$). *^{11}B{^{1}H} NMR (128 MHz, CDCl$_3$):* δ 0.48 ppm, *B*-Me.

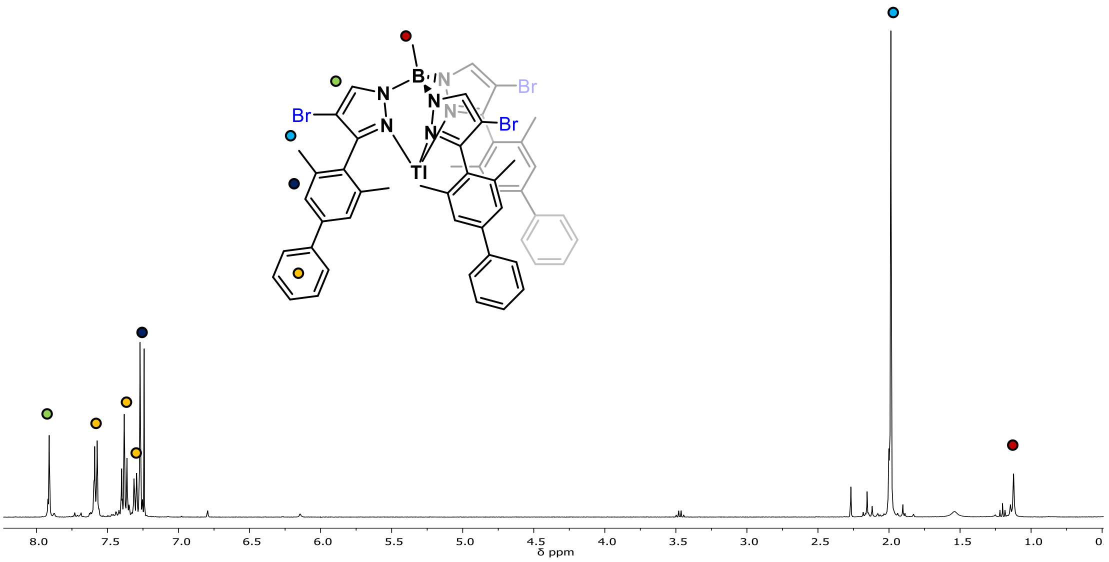

Figure S3: ^{1}H NMR spectrum of Tl(Me-Tp$^{Me2\text{-}biphen,4\text{-}Br}$) (400 MHz, CDCl$_3$).

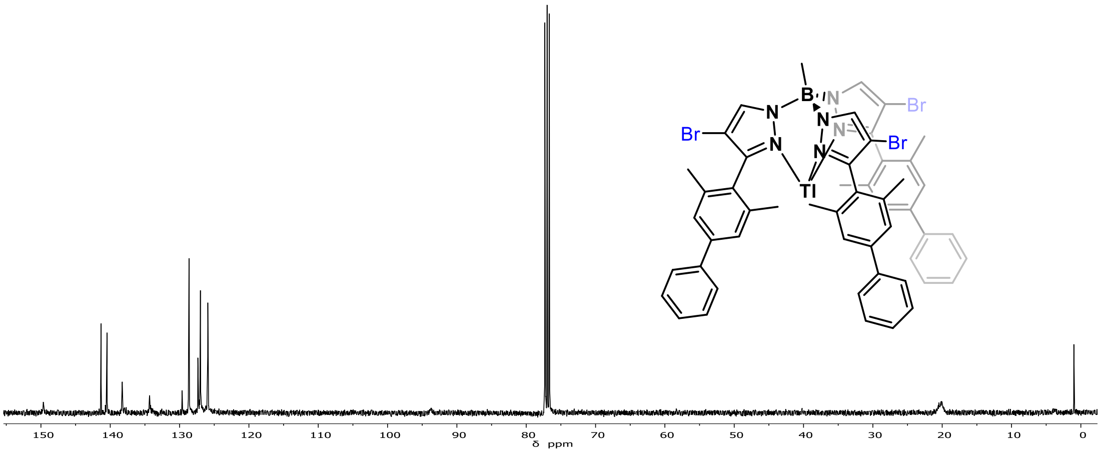

Figure S4: ^{13}C{^{1}H} NMR spectrum of Tl(Me-Tp$^{Me2\text{-}biphen,4\text{-}Br}$) (100 MHz, CDCl$_3$).

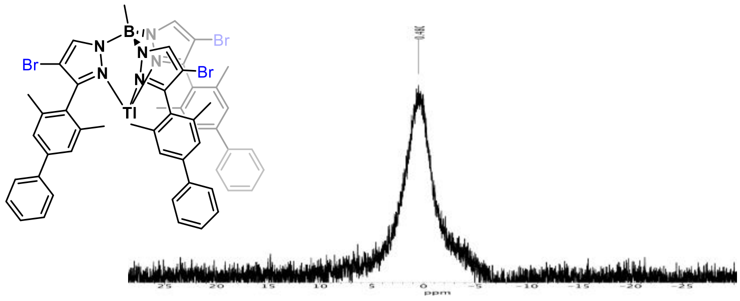

Figure S5: ^{11}B {^{1}H} NMR spectrum of Tl(Me-Tp$^{Me2\text{-}biphen,4\text{-}Br}$) (128 MHz, CDCl$_3$).

Step II. Synthesis of Me-Tp$^{Me2\text{-}biphen}$Ag(C_2H_4) (3) and Me-Tp$^{Me2\text{-}biphen,4\text{-}Br}$Ag($C_2H_4$) (4).

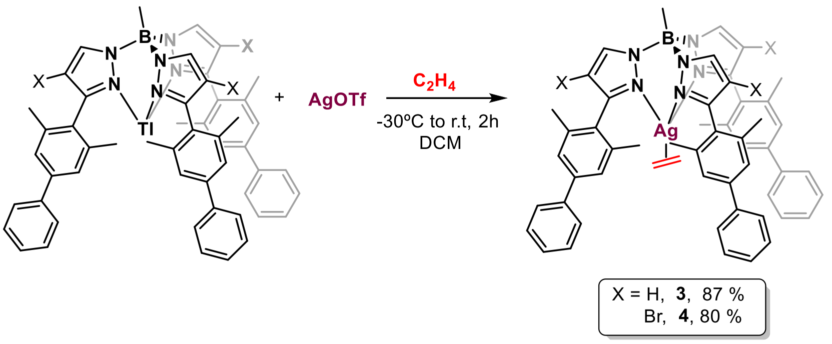

In a Schlenk tube, TlMeTp$^{Me2\text{-}biphen}$ (0.49 g, 0.5 mmol) or TlMeTp$^{Me2\text{-}biphen,4\text{-}Br}$ (0.61 g, 0.5 mmol) were dissolved in 20 mL of DCM. At the same time, inside the dry box, AgOTf (0.13 g, 0.5 mmol) and 20 mL of DCM were placed in another tube covered with aluminum foil to protect it from light. Once out of the dry box, under an ethylene atmosphere and at -30 °C, the thallium salt solution was transferred via cannula to the silver solution. The mixture was stirred at that temperature for 30 min with constant ethylene bubbling through the solution and allowed to reach room temperature, maintaining the ethylene bubbling for another extra hour. Finally, the reaction was stirred for 3 h under an ethylene atmosphere. Then the solution was filterred to remove the salts. Hexane (5 mL) was added to the solution, and the mixture was concentrated by bubbling with ethylene until colorless crystalline material appeared. The mixture was cooled to -30 °C, thus increasing crystallization to obtain 87% (**3**) and 80% (**4**) isolated yields. Analysis calculated for $C_{54}H_{52}BAgN_6$ (**3**): C, 70.77; H, 5.80; N, 9.30 %. Found: C, 70.84; H, 5.80; N, 9.22 %. Analysis calculated for $C_{54}H_{48}BBr_3AgN_6$ (**4**): C, 56.87; H, 4.33; N, 7.37 %. Found: C, 56.78; H, 4.37; N, 7.35 %. *^{1}H NMR of **3** (500 MHz, C_6D_6)***:** δ 1.09 (s, 3H, B-C*H_3*), 2.12 (s, 18H, C*H_3*), 3.75 (br s, 4H, C*$H_{ethylene}$*), 7.09 (m, 3H, C*H_{arom}*),7.16 (m, 6H, C*H_{arom}*), 7.26 (m, 6H, C*H_{arom}*), 7.44 (br s, 6H, C*H_{arom}*), 7.57 (d, 3H, $J_{H\text{-}H}$ = 2 Hz, C*H_{pz}*). *^{13}C{^{1}H} NMR of **3** (125 MHz, C_6D_6):* δ 20.6 (*C*H_3), 103.6 (*C*H_{pz}), 125.8 (*C*H_{arom}), 127.0 (*C*H_{arom}), 127.6 (*C*H_{arom}), 128.6 (*C*H_{arom}), 133.6 (*C*$_{q\text{-}arom}$), 134.7 (*C*H_{pz}), 138.7 (*C*$_{q\text{-}arom}$), 140.8 (*C*$_{q\text{-}arom}$), 141.3 (*C*$_{q\text{-}arom}$), 150.3 (*C*$_{q\text{-}pz}$). *^{11}B{^{1}H} NMR of **3** (160 MHz, C_6D_6):* δ -0.86 (*B*-Me). *^{1}H NMR of **4** (500 MHz, C_6D_6)***:** δ 0.57 (s, 3H, BC*H_3*), 2.07 (s, 18H, C*H_3*), 3.60 (br s, 4H, C*$H_{ethylene}$*), 7.09 (m, 6H, C*H_{arom}*), 7.16 (m, 6H, C*H_{arom}*), 7.26 (m, 6H, C*H_{arom}*), 7.43 (br s, 6H, C*H_{arom}*), 7.62 (s, 3H, C*H_{pz}*). *^{13}C{^{1}H} NMR of **4** (125 MHz, C_6D_6):* δ 20.4 (*C*H_3), 93.1 (*C*$_{qpz}$), 125.4 (*C*H_{arom}), 126.9 (*C*H_{arom}), 128.3 (*C*H_{arom}), 131.0 (*C*H_{arom}), 131.6 (*C*$_{q\text{-}arom}$), 133.5 (*C*H_{pz}), 139.0 (*C*$_{q\text{-}arom}$), 140.5 (*C*$_{q\text{-}arom}$), 140.6 (*C*$_{q\text{-}arom}$), 149.3 (*C*$_{q\text{-}pz}$). *^{11}B{^{1}H} NMR of **4** (160 MHz, C_6D_6):* δ -2.04, *B*-Me.

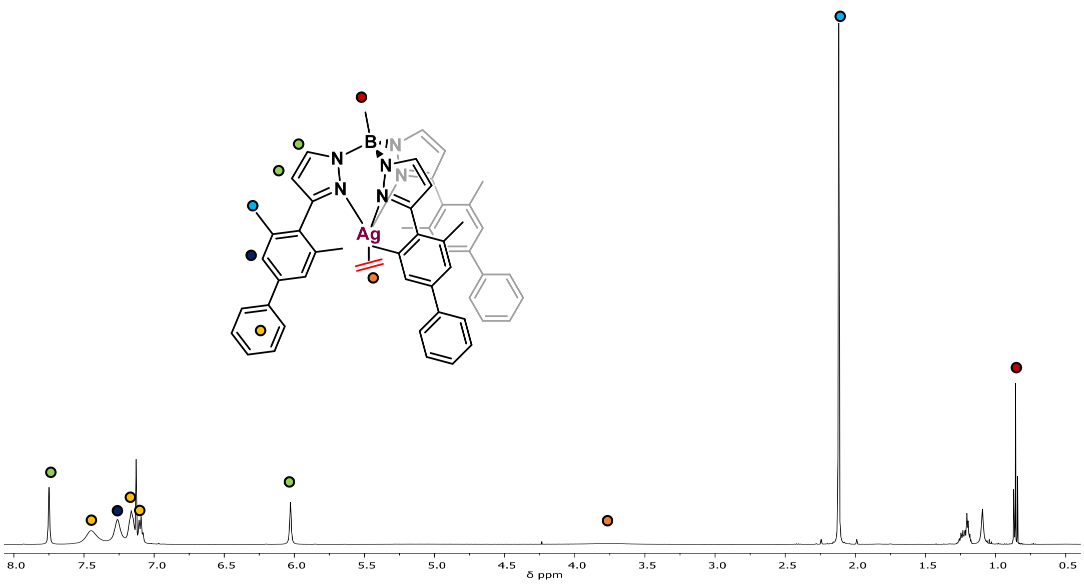

*Figure S6: ^{1}H spectrum of **3** (500 MHz, C_6D_6).*

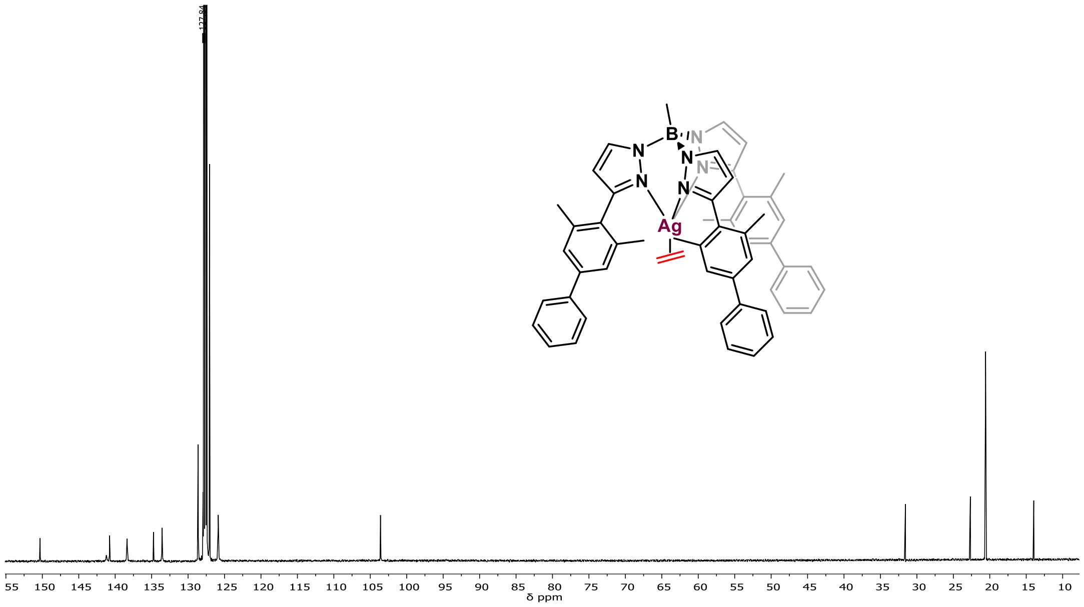

*Figure S7: ^{13}C{^{1}H} spectrum of **3** (125 MHz, C_6D_6)*

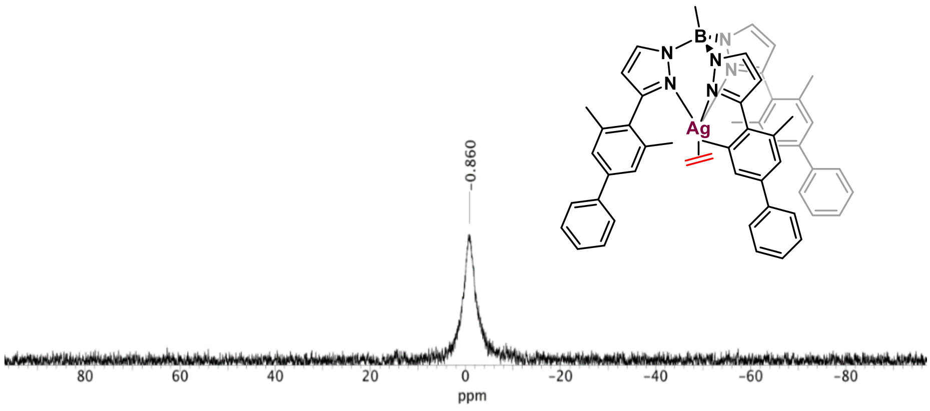

*Figure S8: ^{11}B{^{1}H} spectrum of **3** (160 MHz, C_6D_6)*

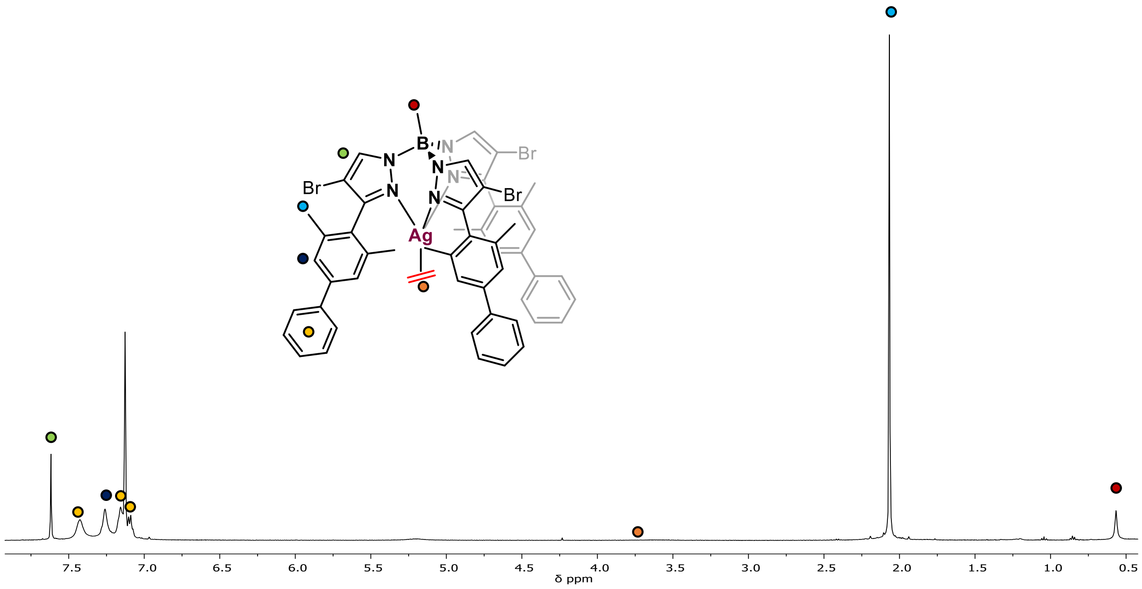

*Figure S9: ^{1}H NMR spectrum of **4** (500 MHz, C_6D_6).*

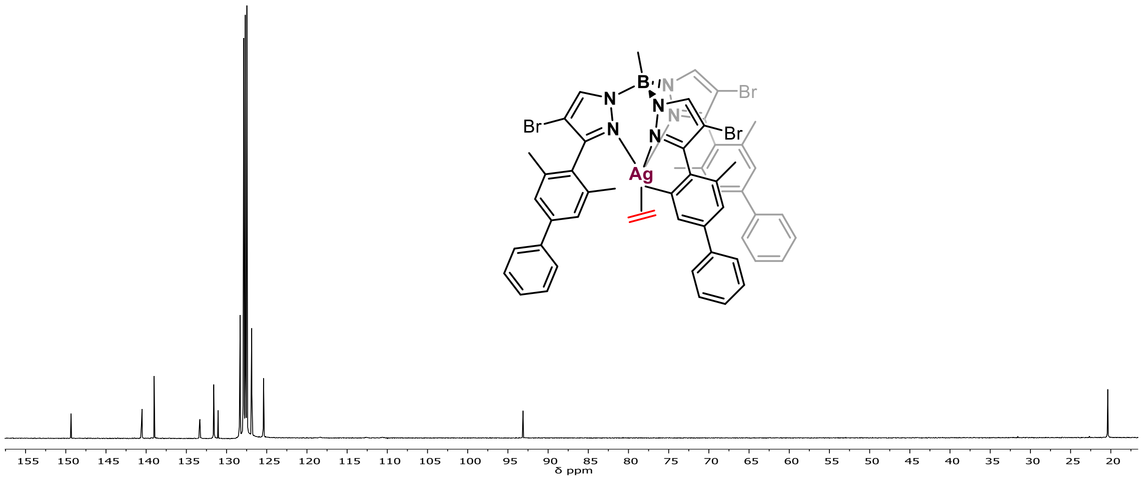

*Figure S10: $^{13}C\{^{1}H\}$ NMR spectrum of **4** (125 MHz, C_6D_6)*

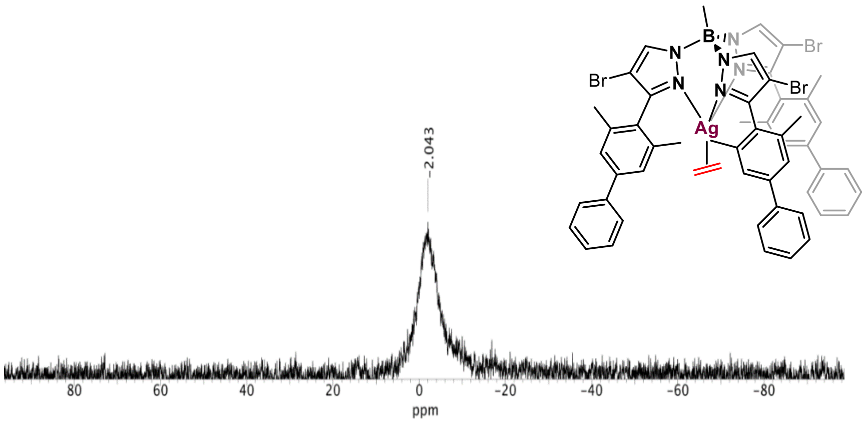

*Figure S11: $^{11}B\{^{1}H\}$ NMR spectrum of **4** (160 MHz, C_6D_6)*

3. Synthesis and identification of products generated by catalytic reactions using monosubstituted diazo compounds.

*tert-butyl octanoate **1A***.[5]

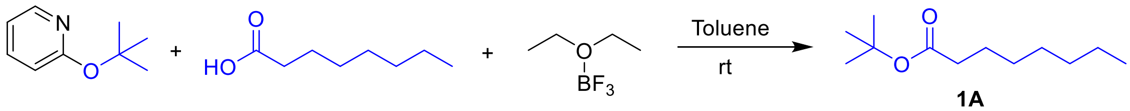

Octanoic acid (0.23 g, 1.64 mmol), tert-butoxypyridine (0.33 g, 2.21 mmol) and boron trifluoride diethyl etherate (0.31 g, 2.21 mmol) in dry toluene (2 mL) were added to a Schlenk tube. The reaction mixture was then allowed to stir at room temperature for 30 min before quenching with anhydrous $NaHCO_3$. Ethyl acetate was then added (30 mL), followed by water (20 mL) and brine (20 mL). The organic layer was separated and dried over anhydrous sodium sulfate, and evaporated under reduced pressure. The resulting residue was then purified by flash column chromatography on silica gel with 0:4 to 1:4 dichloromethane/hexane as eluent, leading to the desired product as a colorless oil with a yield of 89%. *^{1}H NMR* (400 MHz, $CDCl_3$): δ 2.18 (t, *J* = 7.6 Hz, 2H), 1.56 (m, 2H), 1.42 (s, 9H), 1.26 (m, 8H), 0.86 (t, *J* = 6.7 Hz, 3H). *^{13}C{^{1}H} NMR* (100 MHz, $CDCl_3$): δ 173.3, 79.8, 35.6, 31.7, 29.0, 28.9, 28.1, 25.1, 22.6, 14.0.

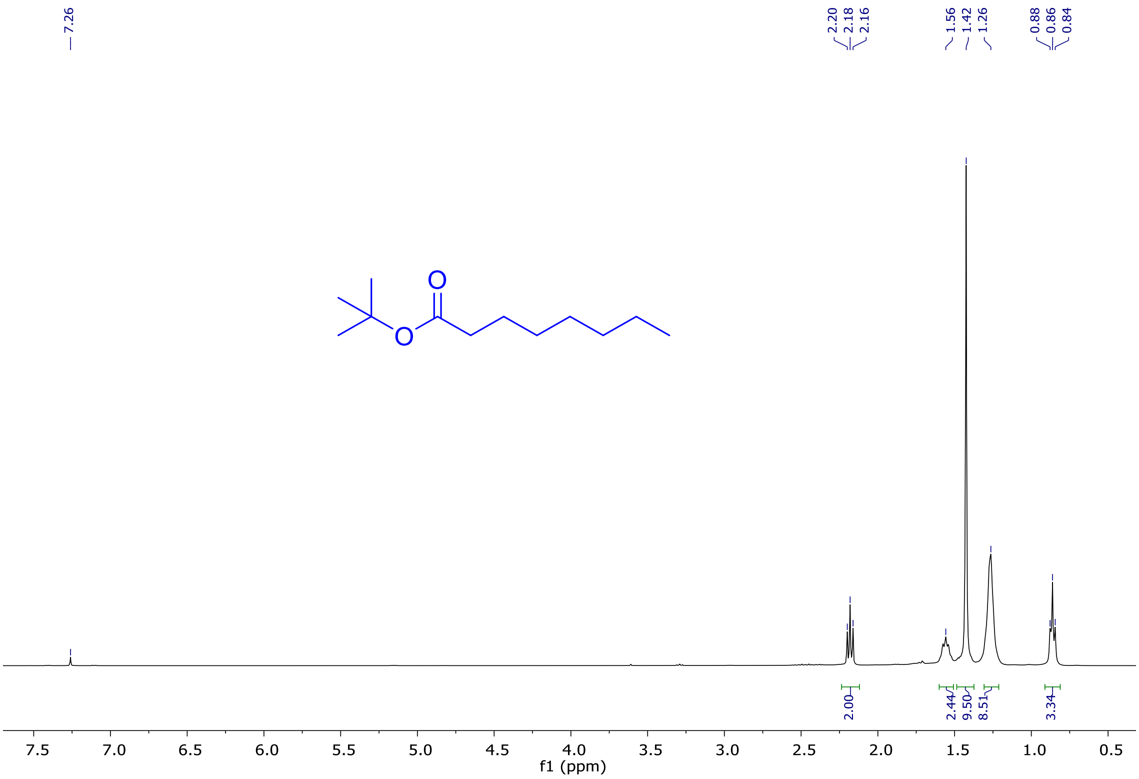

*Figure S12: ^{1}H NMR spectrum of **1A** (400 MHz, $CDCl_3$).*

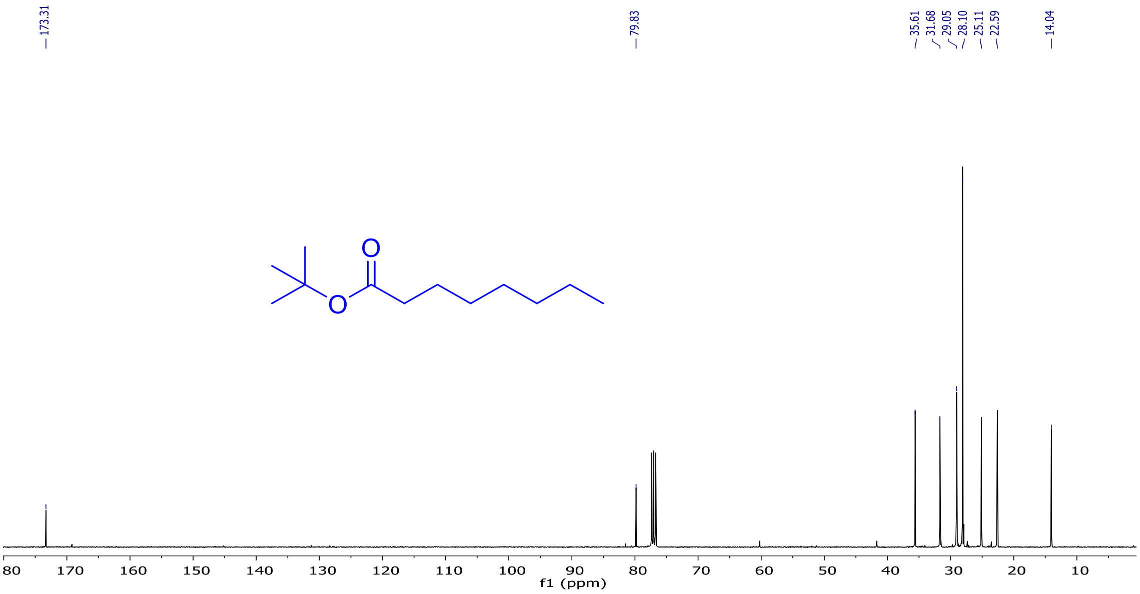

*Figure S13: $^{13}C\{^{1}H\}$ NMR spectrum of **1A** (100 MHz, $CDCl_3$).*

<u>*tert-butyl 3-methylheptanoate **1B** and tert-butyl 3-ethylhexanoate **1C***</u>

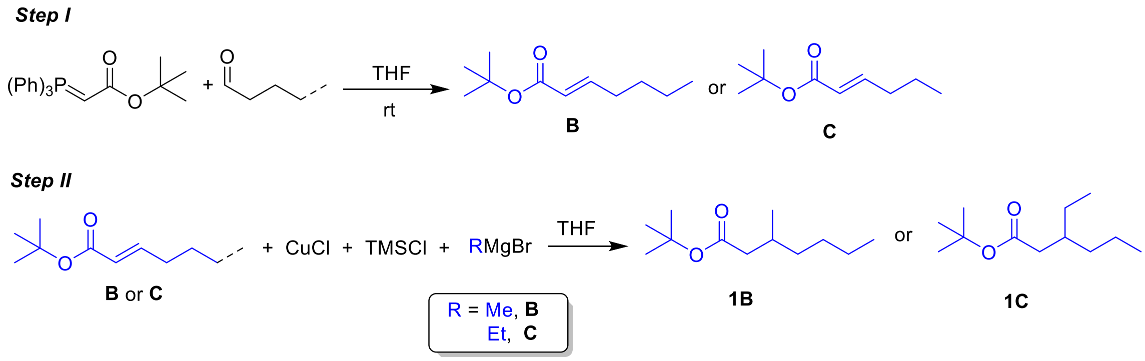

Step I. Following a related protocol,[6] the corresponding aldehyde (13.28 mmol) was dissolved in THF (192 mL) with (tert butoxycarbonylmethylene)triphenylphosphorane (5 g, 13.28 mmol), and the reaction mixture was stirred for 16 h. Then, the solution was concentrated under reduced pressure and Et_2O was added, the resulting solid being discarded upon filtration. The filtrate was taken to dryness and the residue was purified by silica gel chromatography eluting with 0-100% EtOAc-hexane. The products were obtained in 80% (**B**) and 82% (**C**) yields.

Step II. Following a previous protocol,[7] a Schlenk flask was charged with **B** or **C** (6 mmol), THF (7 mL), CuCl (17.8 mg, 0.18 mmol), and $ClSiMe_3$ (1.00 mL, 7.19 mmol), and cooled to 0 °C. The corresponding alkylmagnesiumbromide was added to the mixture. The cold bath was removed. After 1.5 h, saturated aqueous NH_4Cl (20 mL) was added. The aqueous phase was washed with Et_2O (2 x 20 mL) and the combined organic phases were dried ($MgSO_4$). The solvent was removed under reduced pressure and the residue in a glass-oven system, leading to **1B** and **1C** as colorless liquids (71% **1B**, 68% **1C**). *^{1}H NMR of **1B*** (400 MHz, $CDCl_3$): δ 2.19 (dd), 1.99 (dd), 1.90 (m) (ABM

system, J_{AB} = 14; J_{AM} = 6; J_{BM} = 12 Hz), 1.44 (s, 9H), 1.28 (m, 6H), 0.91 (m, 6H). $^{13}C\{^{1}H\}$ *NMR of* ***1B*** (100 MHz, $CDCl_3$): δ 173.8, 78.9, 42.2, 35.4, 29.4, 28.1, 27.1, 21.8. ^{1}H *NMR of* ***1C*** (400 MHz, $CDCl_3$): δ 2.14 (d, *J* =1.1 Hz, 2H), 1.76 (m, 1H), 1.44 (s, 9H), 1.29 (m, 6H), 0.88 (m, 6H). $^{13}C\{^{1}H\}$ *NMR of* ***1C*** (100 MHz, $CDCl_3$): δ 173.1, 79.9, 40.1, 36.3, 35.7, 28.1, 26.3, 19.7, 14.3, 10.8.

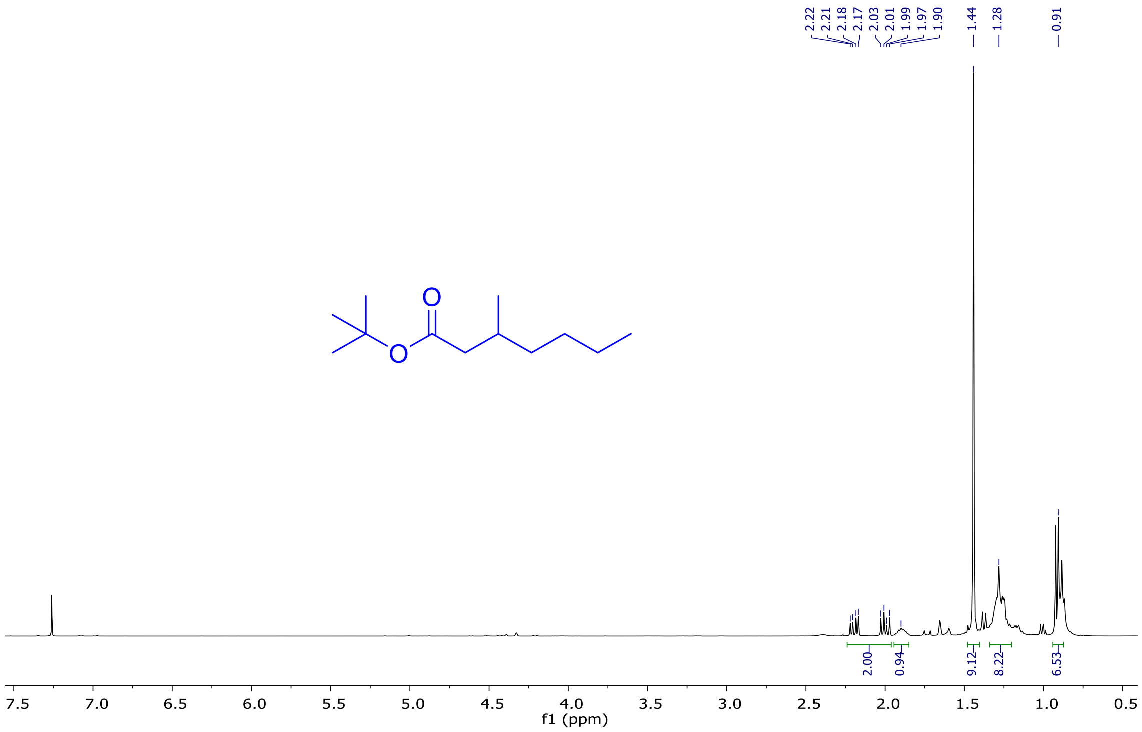

*Figure S14: ^{1}H NMR spectrum of **1B** (400 MHz, $CDCl_3$).*

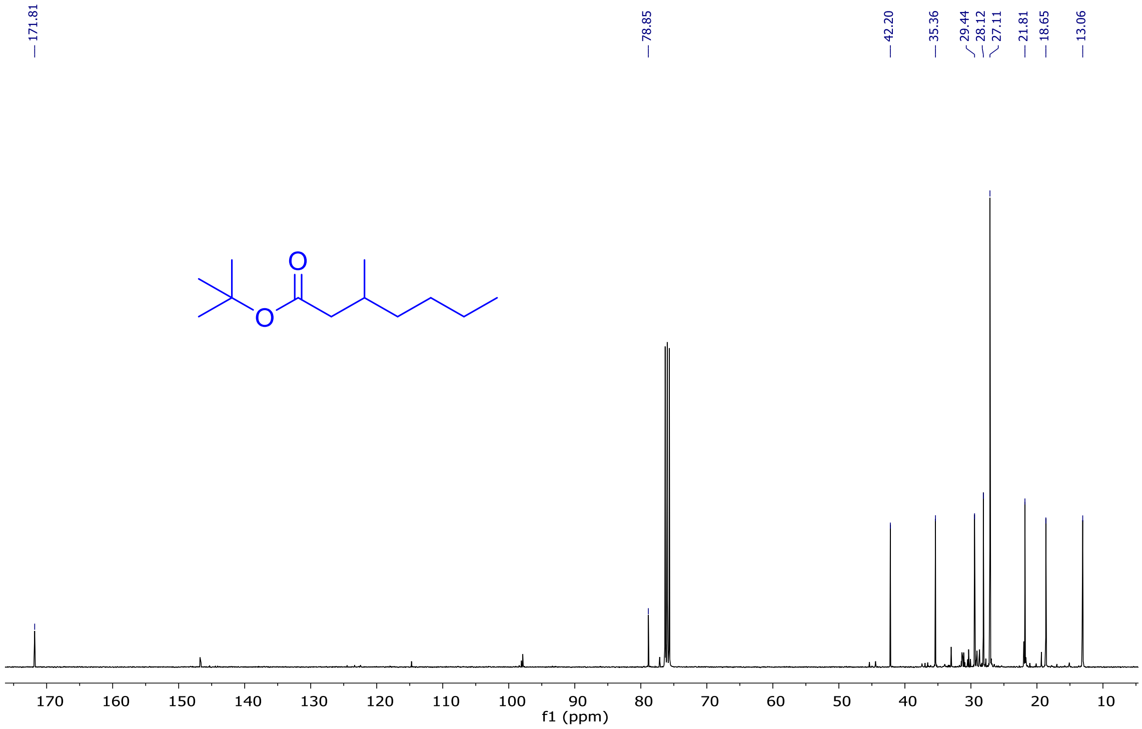

*Figure S15: $^{13}C\{^{1}H\}$ NMR spectrum of **1B** (100 MHz, $CDCl_3$).*

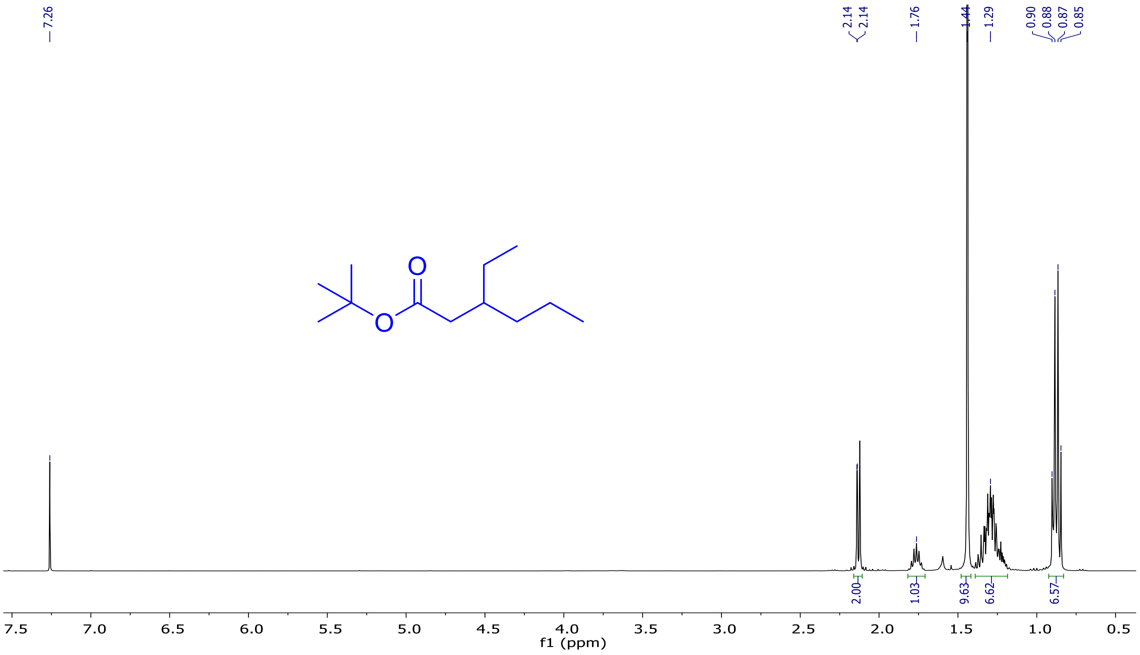

*Figure S16: ^{1}H NMR spectrum of **1C** (400 MHz, $CDCl_3$).*

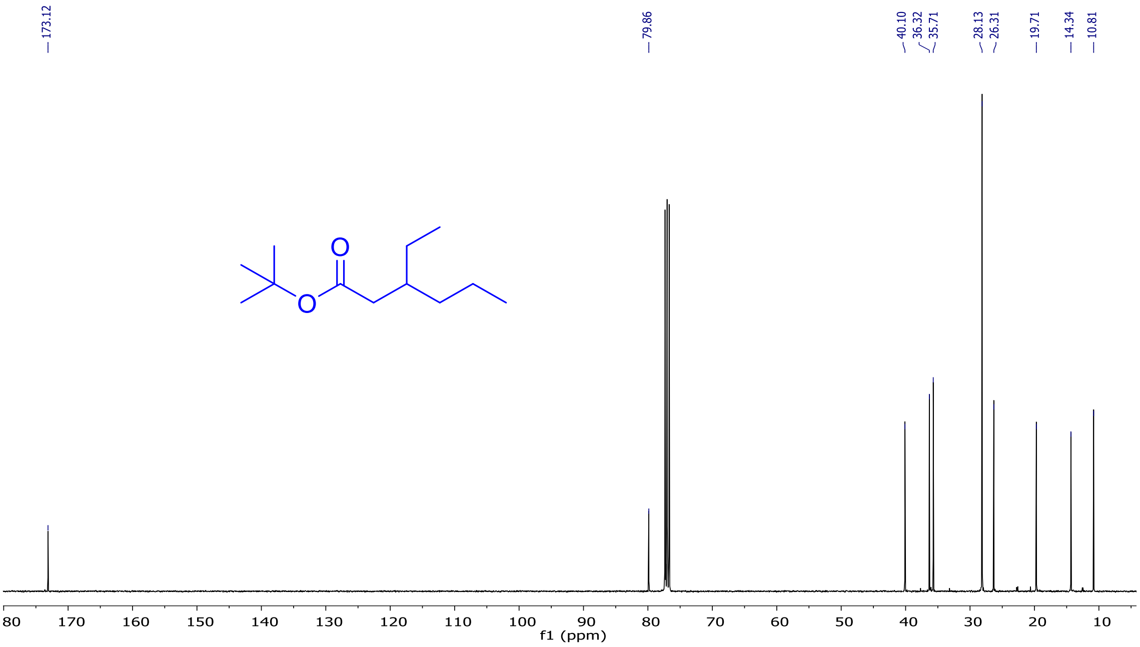

*Figure S17: $^{13}C\{^{1}H\}$ NMR spectrum of **1C** (100 MHz, $CDCl_3$).*

<u>2,4-dimethylpentan-3-yl octanoate **2A**</u>.

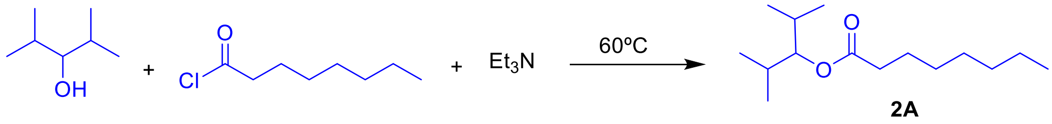

Octanoyl chloride (10 mmol), 2,4-dimethylpentan-3-ol (10 mmol) and triethylamine (50 mmol) were added to an ampoule at 60 °C for 4 h. After the reaction was completed, the product was extracted in diethyl ether, filtered over basic alumina and then purified by vacuum distillation

obtaining the product **2A** as a colorless oil with 75% yield.[8] *^{1}H NMR of **2A*** (400 MHz, $CDCl_3$): δ 4.59 (t, *J* = 6.1 *Hz*, 1H), 2.32 (t, *J* = 7.6 Hz, 2H), 1.89 (m, 2H), 1.64 (m, 2H), 1.30 (m, 8H),0.90-0.85 (m, 3H), 0.86 (d, *J* = 7.1 Hz, 6H), 0.85 (d, *J* = 7.1 Hz, 6H). *$^{13}C\{^{1}H\}$ NMR of **2A*** (100 MHz, $CDCl_3$): δ 173.9, 82.2, 34.5, 31.7, 29.4, 29.3, 29.0, 25.2, 22.6, 19.6, 17.2, 14.1.

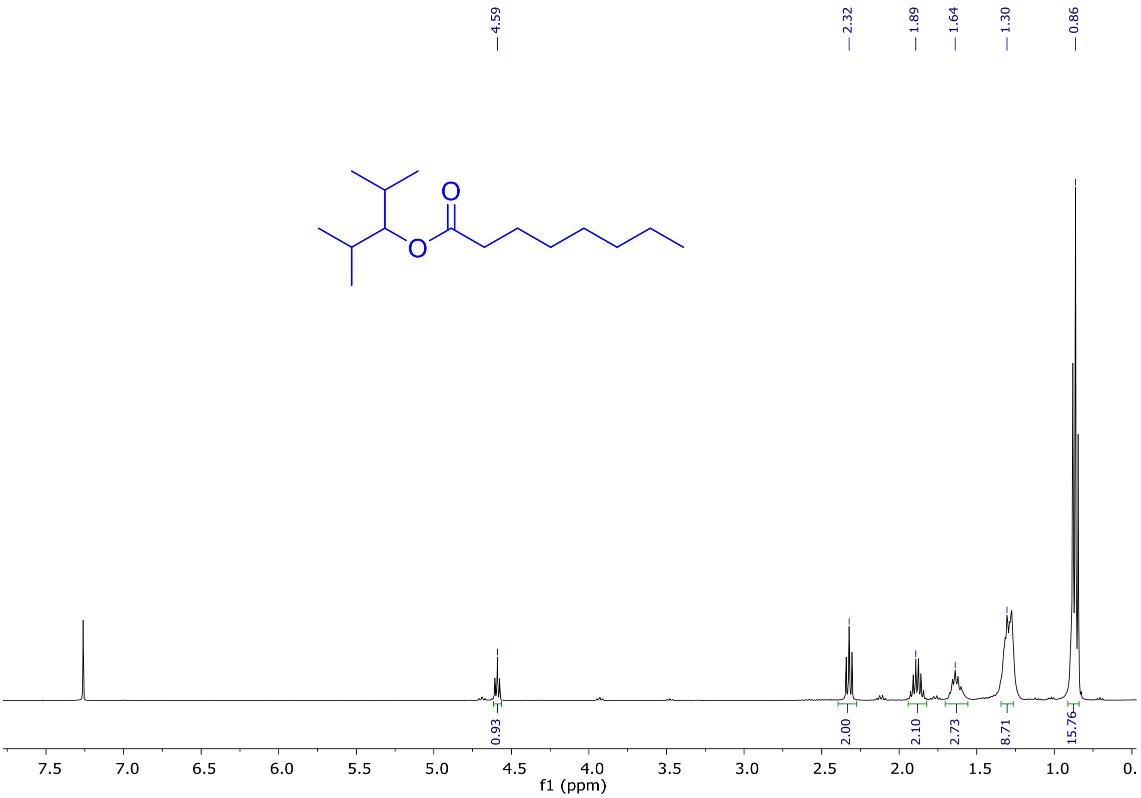

*Figure S18: ^{1}H NMR spectrum of **2A** (400 MHz, $CDCl_3$).*

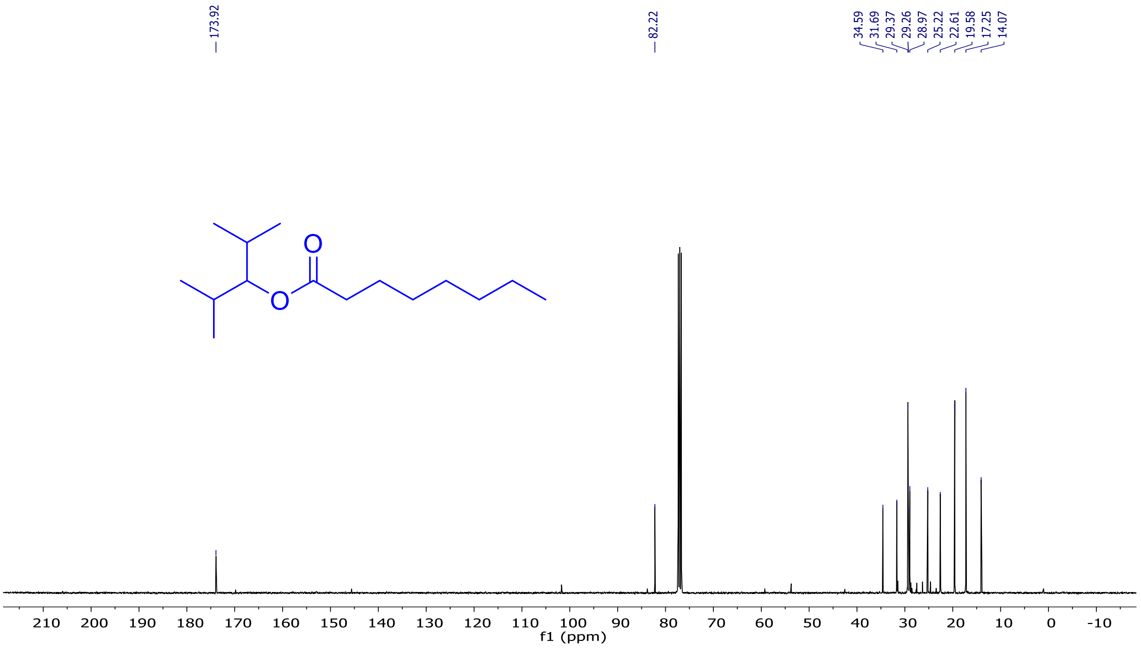

*Figure S19: $^{13}C\{^{1}H\}$ NMR spectrum of **2A** (100 MHz, $CDCl_3$).*

*2,4-dimethylpentan-3-yl 3-methylheptanoate (**2B**) and 2,4-dimethylpentan-3-yl 3-ethylhexanoate (**2C**).*

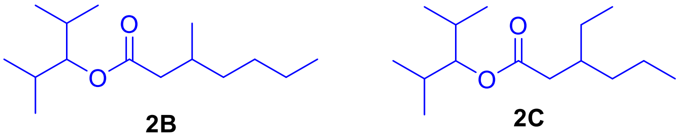

Products **2B** and **2C** could not be synthesized independently, so for the quantification of these products, a calibration curve was made with an internal standard (trimethoxybenzene) of the mixture of products derived from the catalysis, the exact ratio being determined by ^{1}*H NMR*.

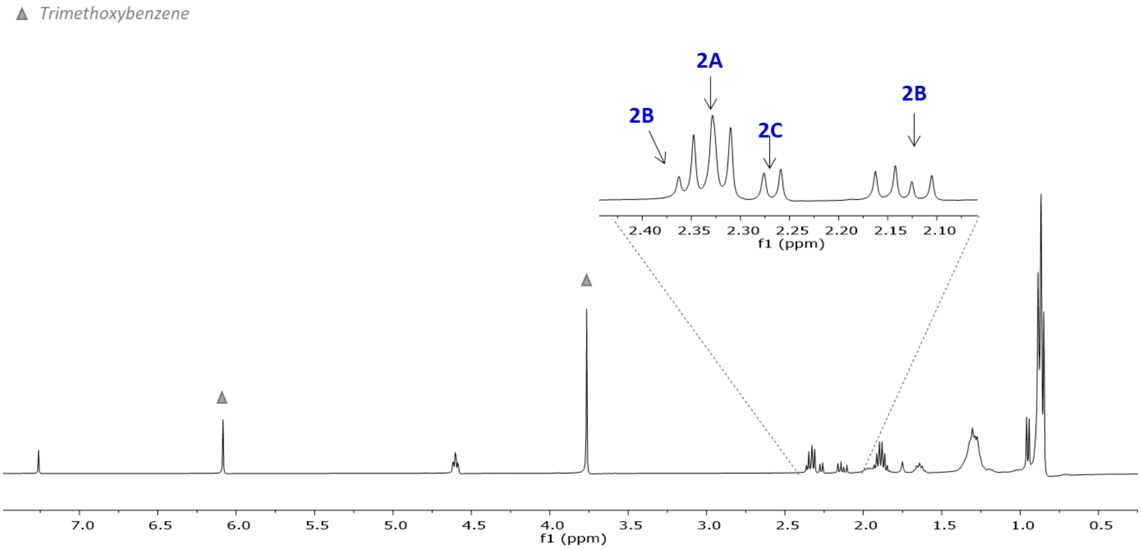

*Figure S20: ^{1}H NMR spectrum of the mixture of **2A, 2B, 2C** (400 MHz, CDCl$_3$).*

*2,6-di-tert-butyl-4-methylphenyl octanoate **3A**.*

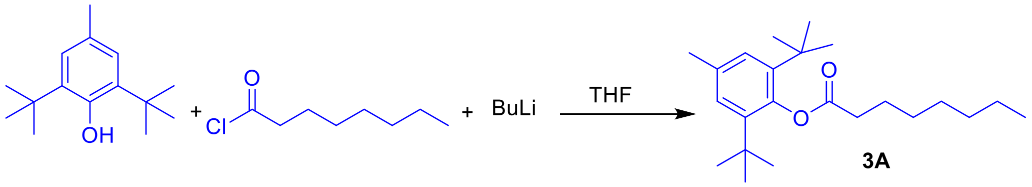

A solution of 2,6-di-*tert*-butyl-4-methylphenol (BHT) (220 mg, 1.00 mmol) in THF (1 mL) was treated with *n*-butyllithium hexane solution (1.05 mmol, 2.5 M) at 0 °C stirring for 30 min at that temperature. Then *n*-octanoyl chloride (0.19 mL, 1.1 mmol) was added to the mixture at 0 °C, stirred for 10 additional minutes, and then the mixture was allowed to warm to room temperature. After 3.5 h of stirring at room temperature, the resulting mixture was filtered through a pad of Celite and the filtrate was concentrated. The residue was purified by bulb to bulb distillation under reduced pressure (15 mmHg, 180-190 °C)[9] to afford **3A** as a colorless oil in 65% yield. ^{1}*H NMR of **2A*** (400 MHz, CDCl$_3$): δ 7.1 (s, 2H), 2.60 (t, *J* = 8.2 Hz, 2H), 2.31 (s, 3H), 1.76 (m, 2H), 1.37-1.16 (m, 8H), 1.32 (s, 18H), 0.85 (m, 3H). ^{13}*C{^{1}H} NMR of **2A*** (100 MHz, CDCl$_3$)

δ 173.7, 145.9, 147.9, 134.4, 127.0, 125.5, 35.8, 35.2, 31.5, 30.3, 29.2, 28.9, 24.2, 22.6, 21.5, 14.09.

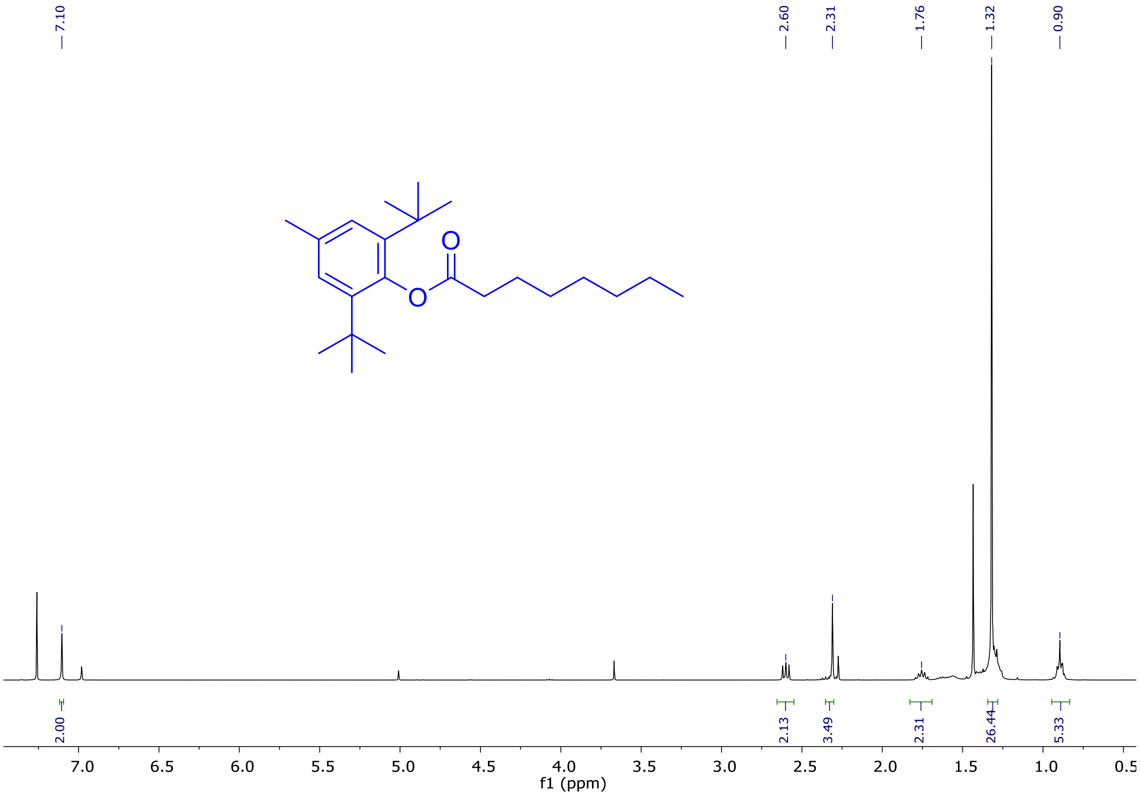

*Figure S21: ¹H NMR spectrum of **3A** (400 MHz, $CDCl_3$).*

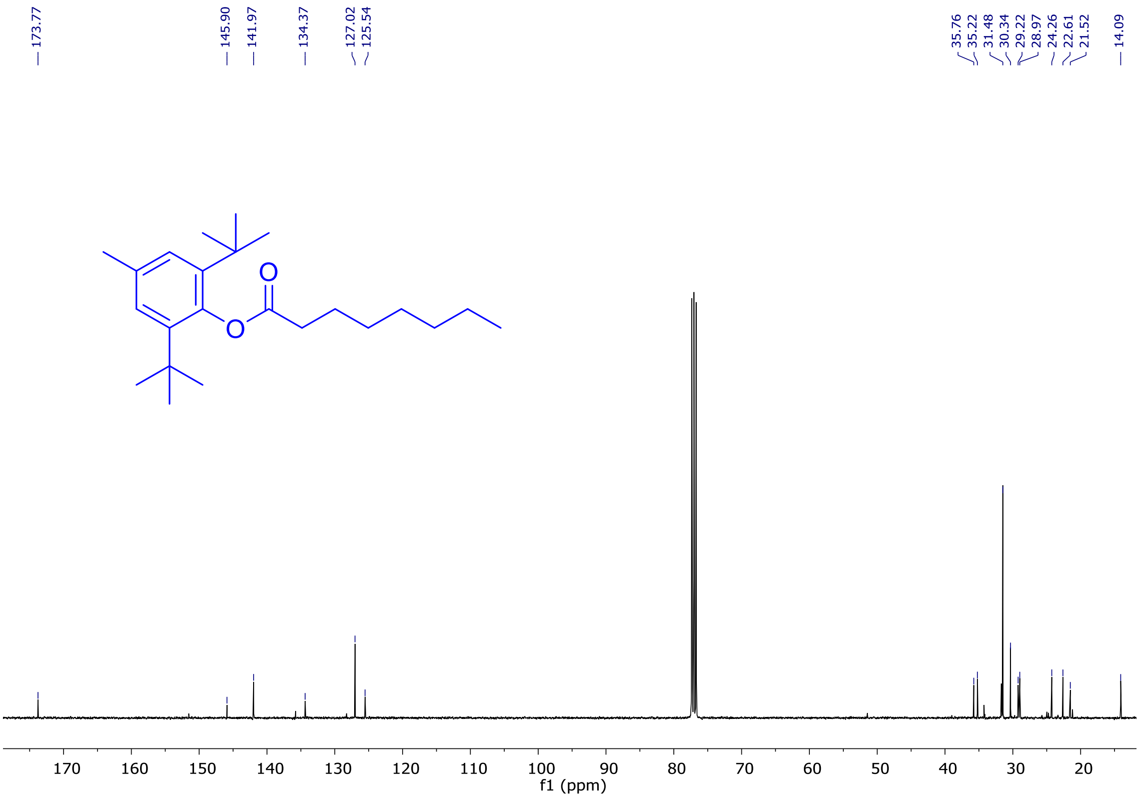

*Figure S22: $^{13}C\{^1H\}$ NMR spectrum of **3A** (100 MHz, $CDCl_3$).*

*<u>2,6-di-tert-butyl-4-methylphenyl 3-methylheptanoate (**3B**) and 2,6-di-tert-butyl-4-methylphenyl 3-ethylhexanoate (**3C**)</u>*.

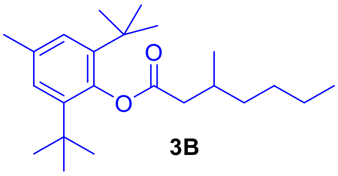

3B

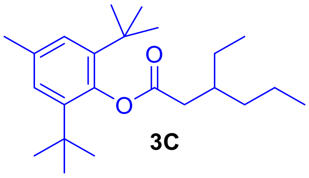

3C

Products **3B** and **3C** could not be synthesized independently, so for the quantification of these products, a calibration curve was made with an internal standard (trimethoxybenzene) of the mixture of products derived from the catalysis, the exact ratio being determined by *^{1}H NMR*.

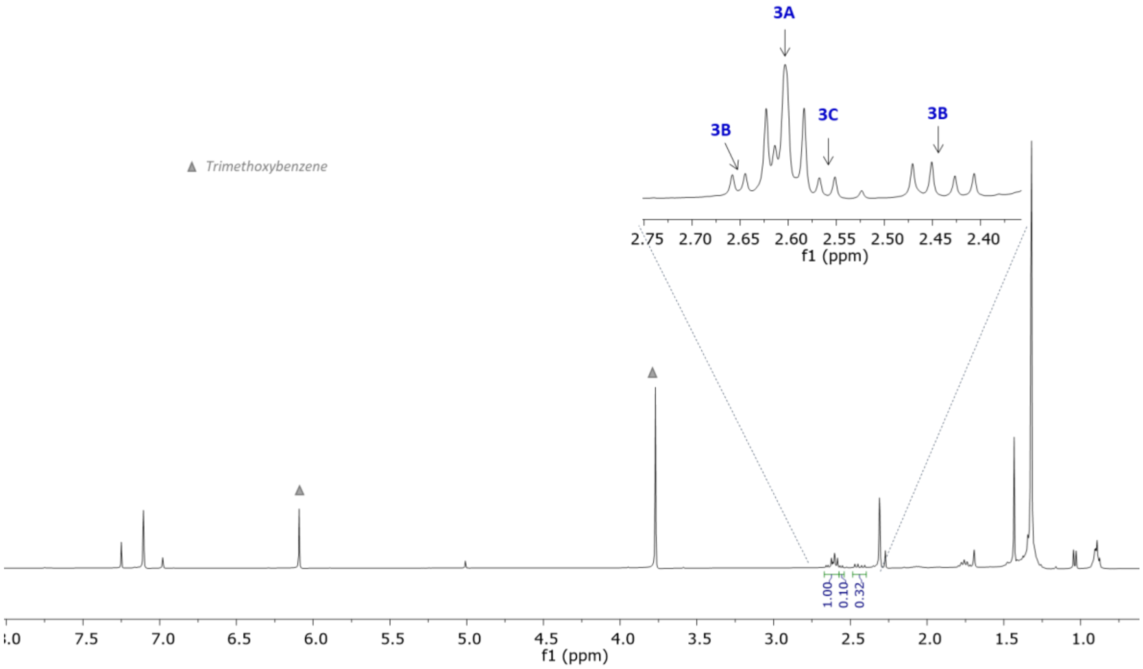

*Figure S23: ^{1}H NMR spectrum of mixture of **3A, 3B, 3C** (400 MHz, $CDCl_3$).*

4. Synthesis and identification of products generated by catalytic reactions using aryl diazo compounds.

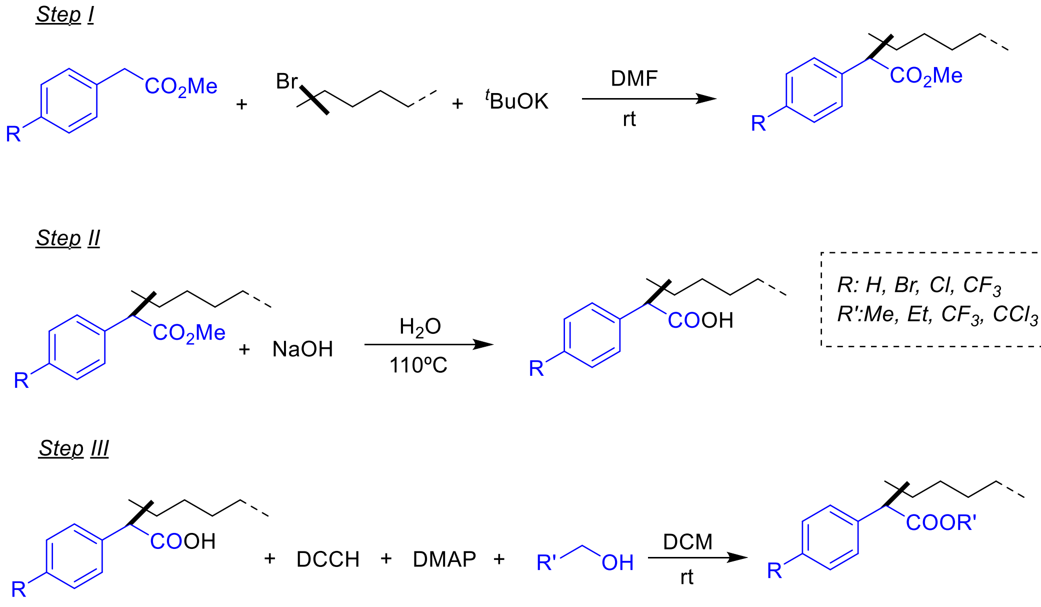

All products were synthesized following the procedures described in the literature.[4] This consists of three reaction steps as shown in the scheme above.

Step I. In a Schlenk tube potassium tert-butoxide K^{t}BuO (6 mmol, 1.2 equiv.) was suspended in 10 mL of dry dimethylformamide (DMF) at 0 °C under an N_2 atmosphere, and 2-(4-X-phenyl)acetate (X = Br, Cl, CF_3, H; 5.0 mmol, 1 equiv.) was added at once, followed (10 min) by the corresponding n-bromoalkane (pentane or hexane, 6 mmol, 1.2 equiv.). The reaction was stirred at 0 °C during 5 min and then at room temperature for 1.5 h. Water (10 mL) was added and the solution was extracted with DCM (2 x 30 mL). The combined organic layers were washed with saturated, aqueous solution of NH_4Cl (20 mL) and water (3 x 10 mL), dried over $MgSO_4$ and filtered off. Then the filtrate was concentrated obtaining in all cases the products as colorless oils in 90-95% yields. Some of the products were used without further purification prior to the next step, others were purified by flash column chromatography (hexanes/diethyl ether = 65/1).

Step II. The products obtained in step I (7 mmol) were added to a one round- bottom flask with NaOH (35 mmol, 5 equiv.) and H_2O (50 mL). The reaction was heated at 120 °C for 18h. Then the solution was treated with Et_2O (3 x 30 mL), phases were separated, and the aqueous phase was brought to pH = 2 with HCl. Extraction with diethyl ether (3 x 40mL), followed by treatment with $MgSO_4$ led, upon evaporation of the solvent, to the products as colorless or slightly yellow oils with yields between 40-60%. The products were used without prior purification in the next step.

Step III. The product obtained in the step II (4 mmol, 1 equiv) was placed in a Schlenk tube and dissolved in 20 mL of dry DCM. Under nitrogen, the corresponding alcohol (4.4 mmol, 1.1 equiv.)

and DMAP (1.2 mmol, 0.3 equiv.) were added at room temperature. The mixture was then cooled at 0°C, then DCC (4.4 mmol, 1.1 equiv) was slowly added dissolved in DCM (2 mL) and with vigorous stirring. The reaction was left stirring at rt for 16h. Then it was filtered off and washed twice with DCM. The solvent was evaporated and the products were purified by silica gel chromatography (Hexane:Et_2O, 95:5). In all cases, colorless oils were obtained with yields within the range 54-95%.

2,2,2-trifluoroethyl 2-(4-bromophenyl)heptanoate 1a. 94 % Yield. ^{1}H *NMR* (400 MHz, $CDCl_3$): δ 7.44 (d, J = 8.4 Hz, 2H), 7.19 (d, J = 8.4 Hz, 2H), 4.51 (dq, 1H, J_{H-H} = 12.7, J_{H-F} = 8.5 Hz, C*H*HCF$_3$), 4.40 (dq, 1H, J_{H-H} = 12, J_{H-F} = 8.4 Hz, CH*H*CF$_3$), 3.60 (t, J =7.7 Hz, 1H), 2.05 (m, 1H), 1.78 (m, 1H), 1.28 (m, 6H), 0.86 (t, J = 7.0 Hz, 3H). $^{13}C\{^{1}H\}$ *NMR* (100 MHz, $CDCl_3$): δ 172.3, 137.0, 131.8, 129.6, 121.7 (q, *C*F$_3$, J_{C-F} = 277 Hz), 121.5, 77.2, 60.4 (q, *C*H$_2$CF$_3$, J = 36.5 Hz), 50.7, 33.2, 31.4, 26.9, 22.3, 13,9. $^{19}F\{^{1}H\}$ *NMR* (375 MHz, $CDCl_3$): δ -73.78.

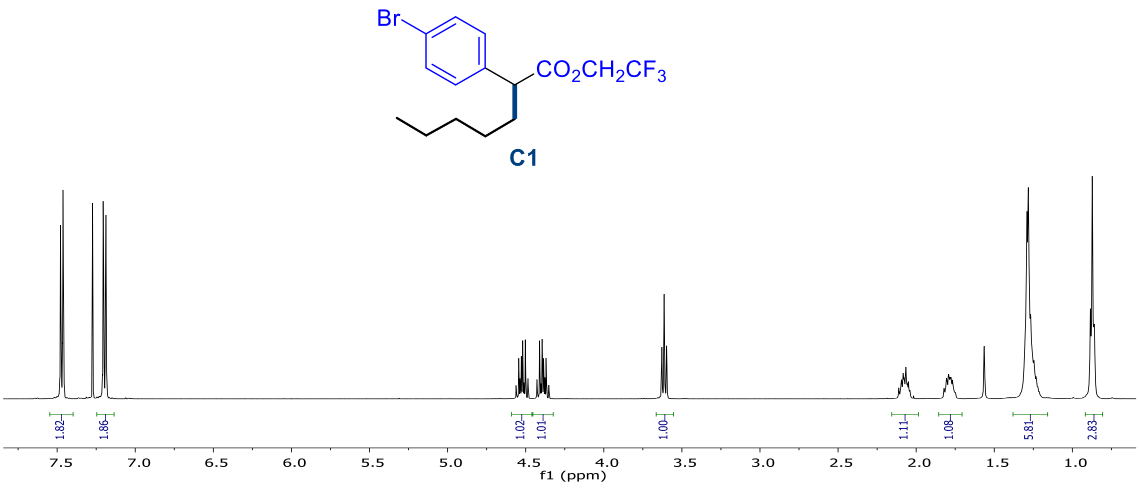

*Figure S24: ^{1}H NMR spectrum of **1a** (400 MHz, $CDCl_3$).*

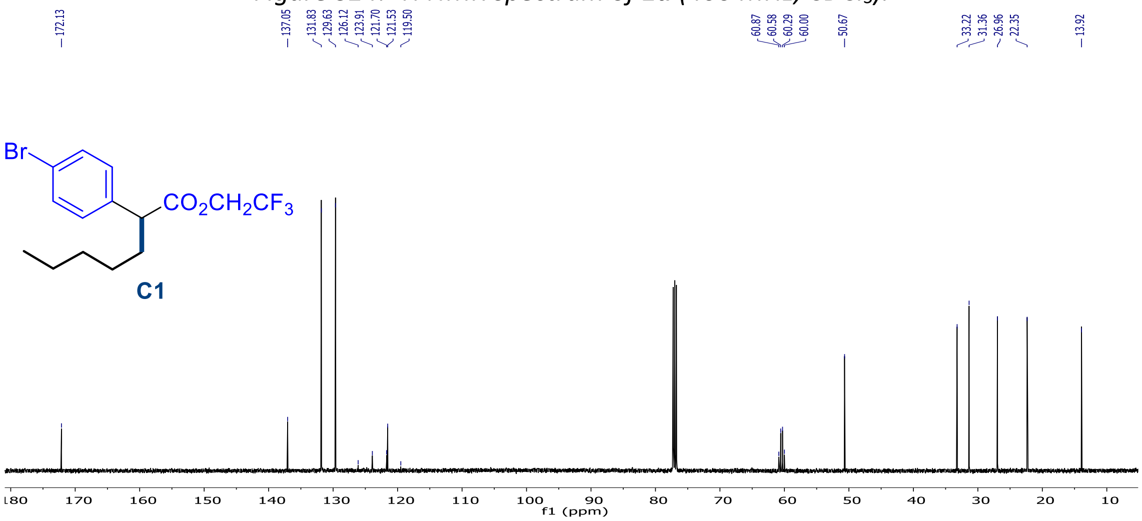

*Figure S25: $^{13}C\{^{1}H\}$ NMR spectrum of **1a** (100 MHz, $CDCl_3$).*

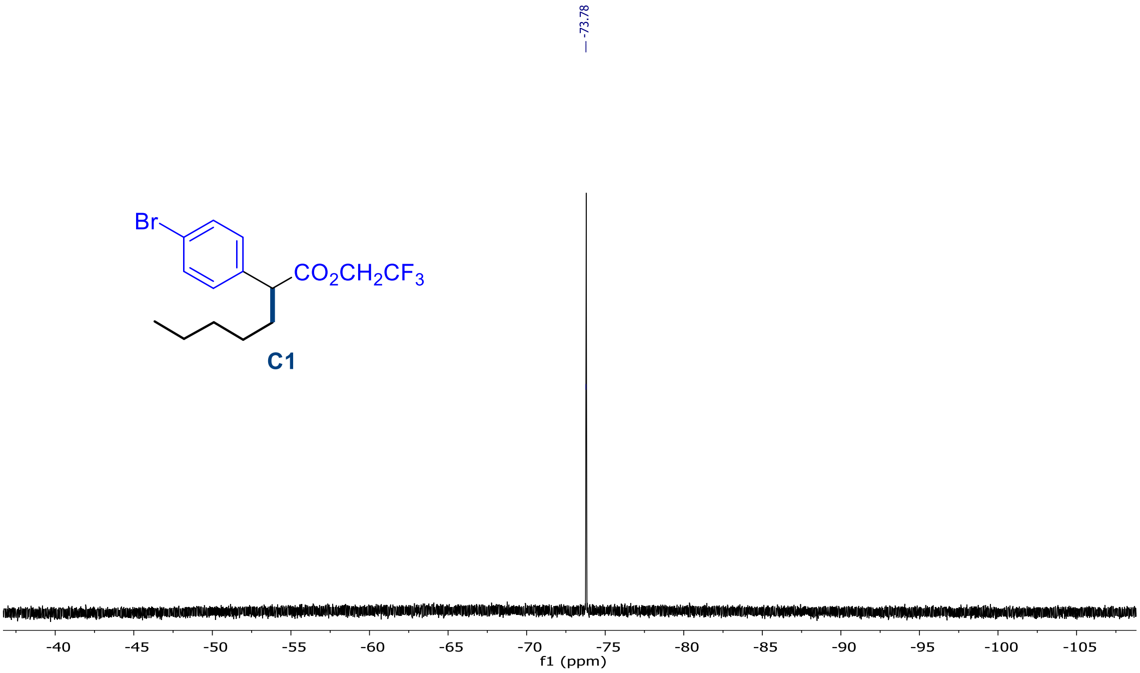

Figure S26: $^{19}F\{^{1}H\}$ *NMR spectrum of* ***1a*** *(375 MHz,* $CDCl_3$*).*

2,2,2-trifluoroethyl-2-(4-bromophenyl)-3-ethylpentanoate 1c. 85% Yield. ^{1}H *NMR* (400 MHz, $CDCl_3$): δ 7.44 (d, J = 9 Hz, 2H), 7.24 (d, J = 9 Hz, 2H), 4.53 (dq, 1H, $J_{H\text{-}H}$ = 12.7, $J_{H\text{-}F}$ = 8.5 Hz, C*H*HCF$_3$), -4.43 (dq, 1H, $J_{H\text{-}H}$ = 12.7, $J_{H\text{-}F}$ = 8.5 Hz, CH*H*CF$_3$), 3.52 (d, J = 11 Hz, 1H), 2.10 (m, 1H), 1.49 – 1.38 (m, 2H), 1.24 (m, 1H), 1.06 (m, 1H), 0.91 (t, J = 7.4 Hz, 3H), 0.73 (t, J = 7.4 Hz, 3H); $^{13}C\{^{1}H\}$ *NMR* (100 MHz, $CDCl_3$): δ 172.2, 136.3, 131.7, 130.3, 121.6, 77.2, 60.2, 54.5, 42.8, 22.5, 20.8, 9.9, 9.4. $^{19}F\{^{1}H\}$ *NMR* (375 MHz, $CDCl_3$): δ -73.73.

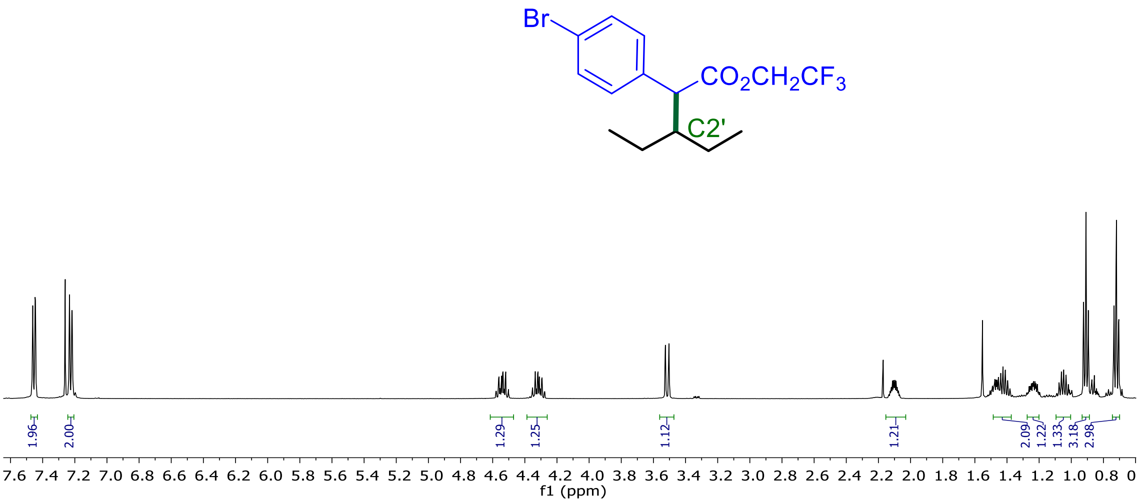

Figure S27: ^{1}H *NMR spectrum of* ***1c*** *(400 MHz,* $CDCl_3$*).*

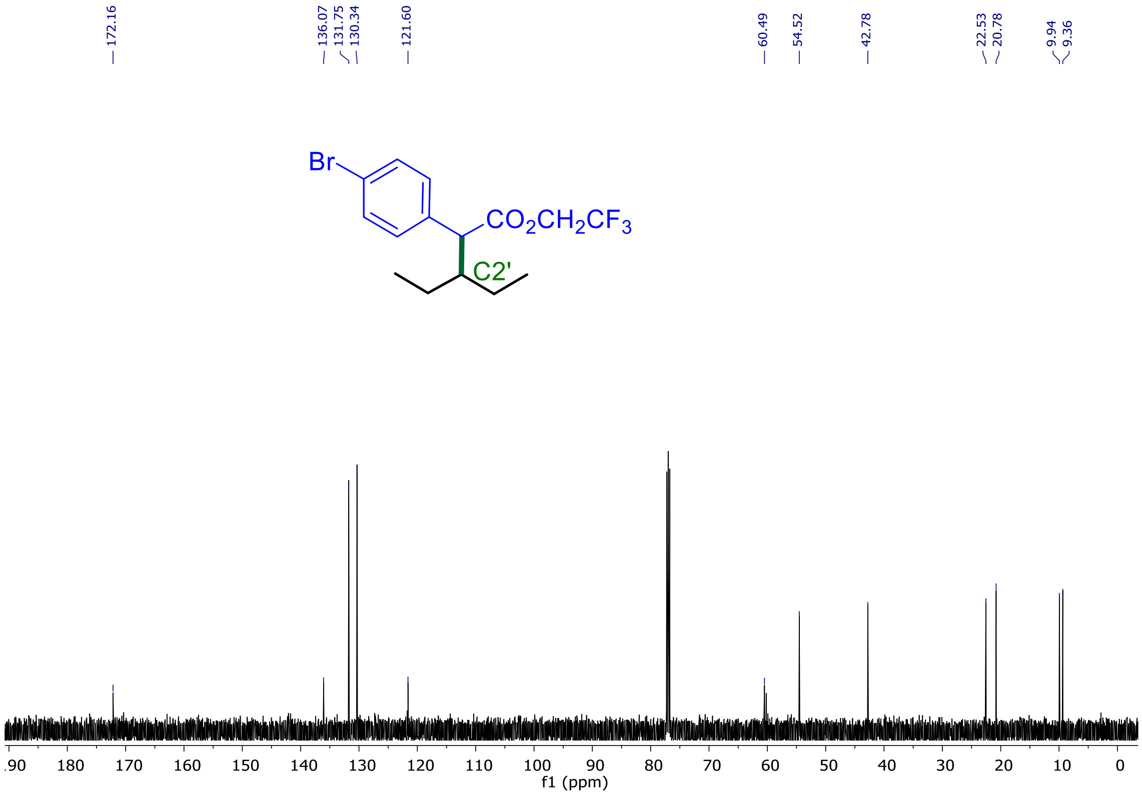

*Figure S28: $^{13}C\{^1H\}$ NMR spectrum of **1c** (100 MHz, $CDCl_3$).*

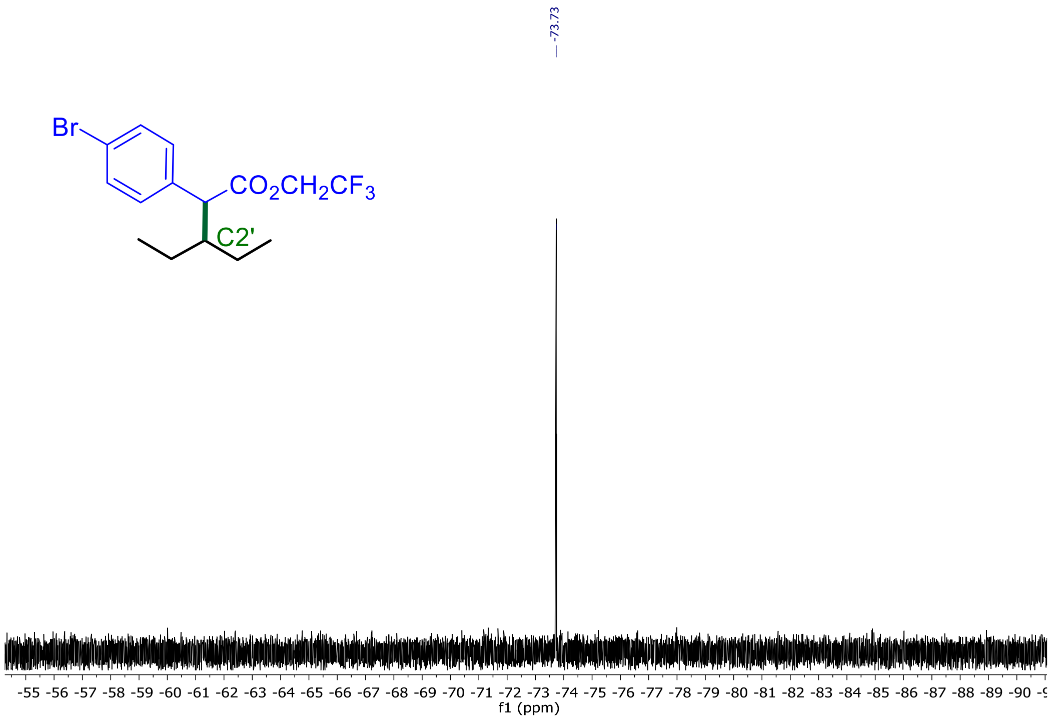

*Figure S29: $^{19}F\{^1H\}$ NMR spectrum of **1c** (375 MHz, $CDCl_3$).*

2,2,2-trifluoroethyl-2-(4-trifluorophenyl)heptanoate 2a. 86 % Yield. *^{1}H NMR* (400 MHz, $CDCl_3$): δ 7.59 (d, *J* = 8.1 Hz, 2H), 7.43 (d, *J* = 8.1 Hz, 2H), 4.53 (m, 1H, C*H*HCF_3), 4.39 (m, 1H, CH*H*CF_3), 3.70 (t, *J* = 7.7 Hz, 1H), 2.11 (m, 1H), 1.81 (m, 1H), 1.32 (m, 6H), 0.87 (t, *J* = 6.5 Hz, 3H). *^{13}C{^{1}H} NMR* (100 MHz, $CDCl_3$): δ 170.9, 141.0, 128.9 (q, C_qCF_3, J_{C-F} = 32.6 Hz), 127.3, 124.6 (q, *C*$HCCF_3$, J_{C-F} = 3.7 *Hz*), 123.1 (q, *C*F_3, J_{C-F}= 277 Hz), 120.4 (q, *C*F_3, J_{C-F}= 277 Hz), 59.7 (q, *C*H_2CF_3, *J* = 36.6 Hz), 54.7, 50.6, 33.9, 30.3, 25.9, 31.3, 12.9. *^{19}F{^{1}H} NMR* (375 MHz, $CDCl_3$): δ -62.62, -73.73.

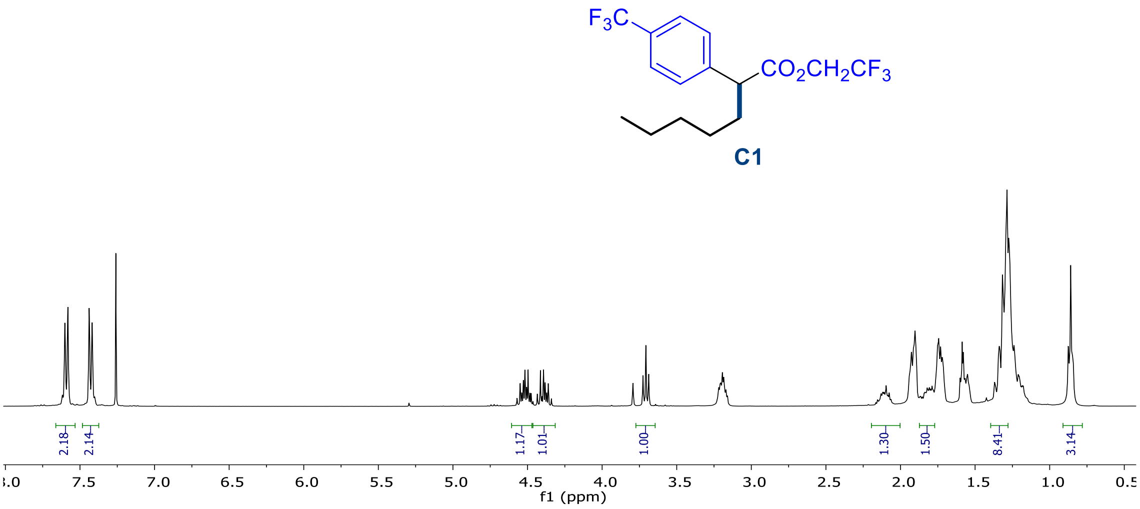

*Figure S30: ^{1}H NMR spectrum of **2a** (400 MHz, $CDCl_3$).*

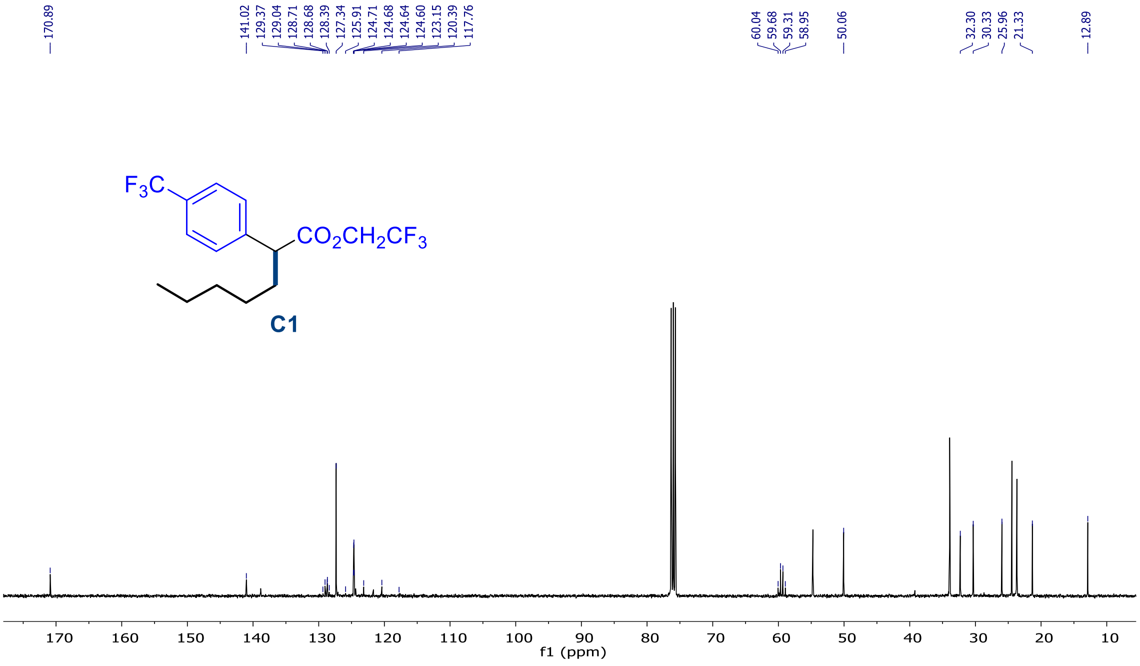

*Figure S31: ^{13}C{^{1}H} NMR spectrum of **2a** (100 MHz, $CDCl_3$).*

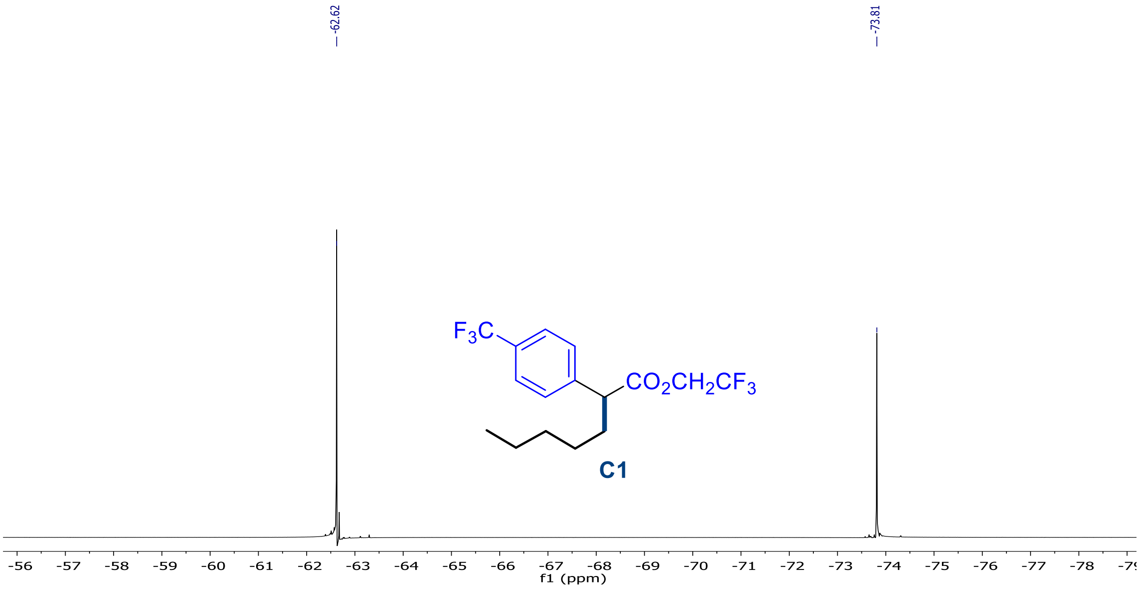

*Figure S32:^{19}F{^{1}H} NMR spectrum of **2a** (375 MHz, $CDCl_3$).*

2,2,2-trifluoroethyl-2-(4-trifluorophenyl)-3-methylhexanoate 2b. 78% Yield, as a 60:40 mixture of diastereoisomers. Major isomer. *^{1}H NMR* (400 MHz, $CDCl_3$): δ 7.58 (d, *J* = 8.1 Hz, 2H), 7.44 (d, *J* = 8.1 Hz, 2H), 4.54 (m, 1H, C*H*HCF$_3$), 4.34 (m, 1H, CH*H*CF$_3$), 3.45 (d, *J* =10.4 Hz, 1H), 2.27 (m, 1H), 1.44-1.15 (m, 6H), 0.92 (t, *J* = 7.2 Hz, 3H), 0.70 (d, *J* = 6.7 Hz, 3H). Minor isomer. *^{1}H NMR* (400 MHz, $CDCl_3$): δ 7.58 (d, *J* = 8.1 Hz, 2H), 7.44 (d, *J* = 8.1 Hz, 2H), 4.54 (m, 1H, C*H*HCF$_3$), 4.34 (m, 1H, CH*H*CF$_3$), 3.46 (d, *J* =10.4 Hz, 1H), 2.27 (m, 1H), 1.44-1.15 (m, 6H), 1.03 (d, *J* = 6.5 Hz, 3H), 0.77 (t, *J* = 7.3 Hz, 3H). *^{13}C{^{1}H} NMR* (100 MHz, $CDCl_3$): δ 170.8, 140.1, 129,0 (q, C_qCHF$_3$, J_{C-F} = 32.7 Hz), 128.0, 124.5 (q, *C*HCCF$_3$, J_{C-F} = 3.2 Hz), 121.7 (q, *C*F$_3$, J_{C-F} = 277 Hz), 59.9 (q, *C*H$_2$CF$_3$, J_{C-F} = 36.5 Hz), 57.1, 36.2, 35.3, 35.2, 34.4, 18.7, 18.4, 16.5, 15.5, 13.0, 12.9.*^{19}F{^{1}H} NMR* (375 MHz, $CDCl_3$): δ -62.62, -73.81.

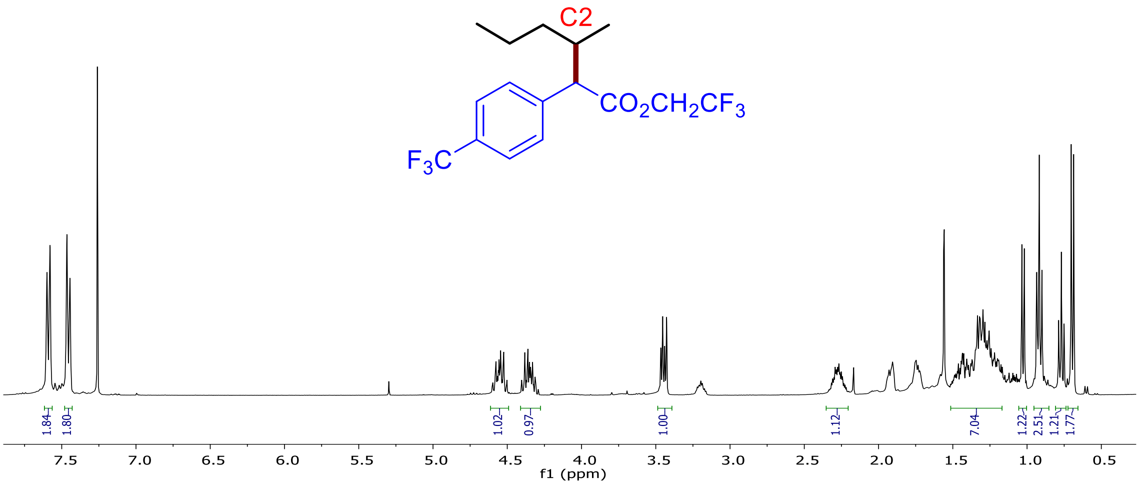

*Figure S33: ^{1}H NMR spectrum of **2b** (400 MHz, $CDCl_3$).*

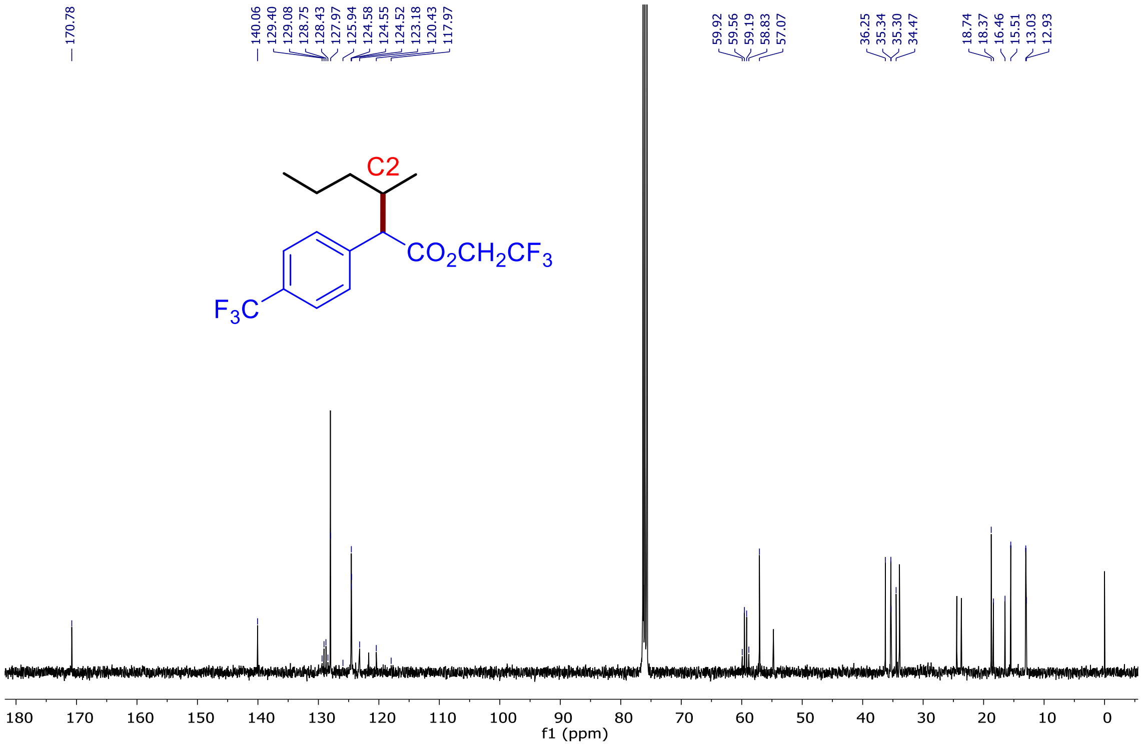

*Figure S34: $^{13}C\{^{1}H\}$ NMR spectrum of **2b** (100 MHz, $CDCl_3$).*

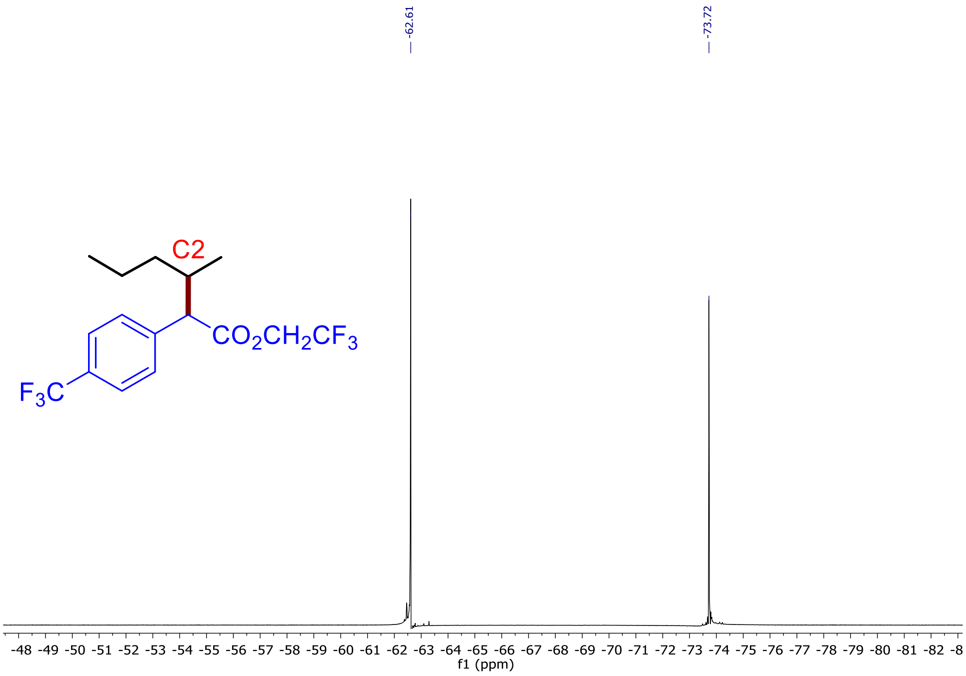

*Figure S35: $^{19}F\{^{1}H\}$ NMR spectrum of **2b** (375 MHz, $CDCl_3$).*

2,2,2-trifluoroethyl-2-(4-trifluorophenyl)-3-ethylpentanoate 2c. 71% Yield. *^{1}H NMR* (400 MHz, $CDCl_3$): δ 7.59 (d, J = 8.3 Hz, 2H), 7.48 (d, J = 8.3 Hz, 2H), 4.60 (dq, 1H, $J_{H\text{-}H}$ = 12.7, $J_{H\text{-}F}$ = 8.4 Hz, CH*H*CF_3, C*H*HCF_3), 4.26 (dq, 1H, $J_{H\text{-}H}$ = 12.7, $J_{H\text{-}F}$ = 8.4 Hz, CH*H*CF_3, CH*H*CF_3), 3.63 (d, J = 11 Hz, 1H), 2.17 (m, 1H), 1.47 (m, 2H), 1.26 (m, 1H), 1.05 (m, 1H), 0.93 (t, J = 7.5 Hz, 3H), 0.73 (t, J = 7.5 Hz, 3H); *$^{13}C\{^{1}H\}$ NMR* (100 MHz, $CDCl_3$): δ 171.9, 140.1, 138.8 (q, C_qCCF_3, J = 32.6 Hz), 128.1, 124.5

(q, *C*HCCF$_3$, J_{C-F}= 3.7 Hz), 123.2 (q, *C*F$_3$, J_{C-F} = 277 Hz), 121.7 (q, *C*F$_3$, J_{C-F} = 277 Hz), 59.6 (q, *C*H$_2$CF$_3$, J_{C-F} = 36.5 Hz), 53.9, 41.9, 21.4, 19.8, 8.9, 8.3. $^{19}F\{^1H\}$ *NMR* (375 MHz, CDCl$_3$) δ -62.62, -73.77.

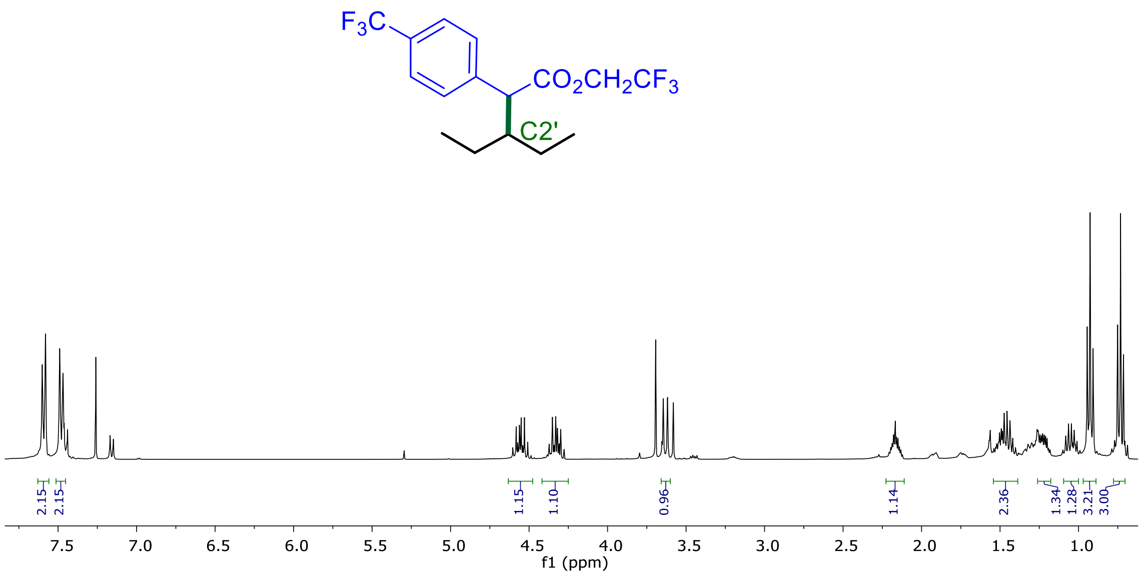

*Figure S36: ^{1}H NMR spectrum of **2c** (400 MHz, CDCl$_3$).*

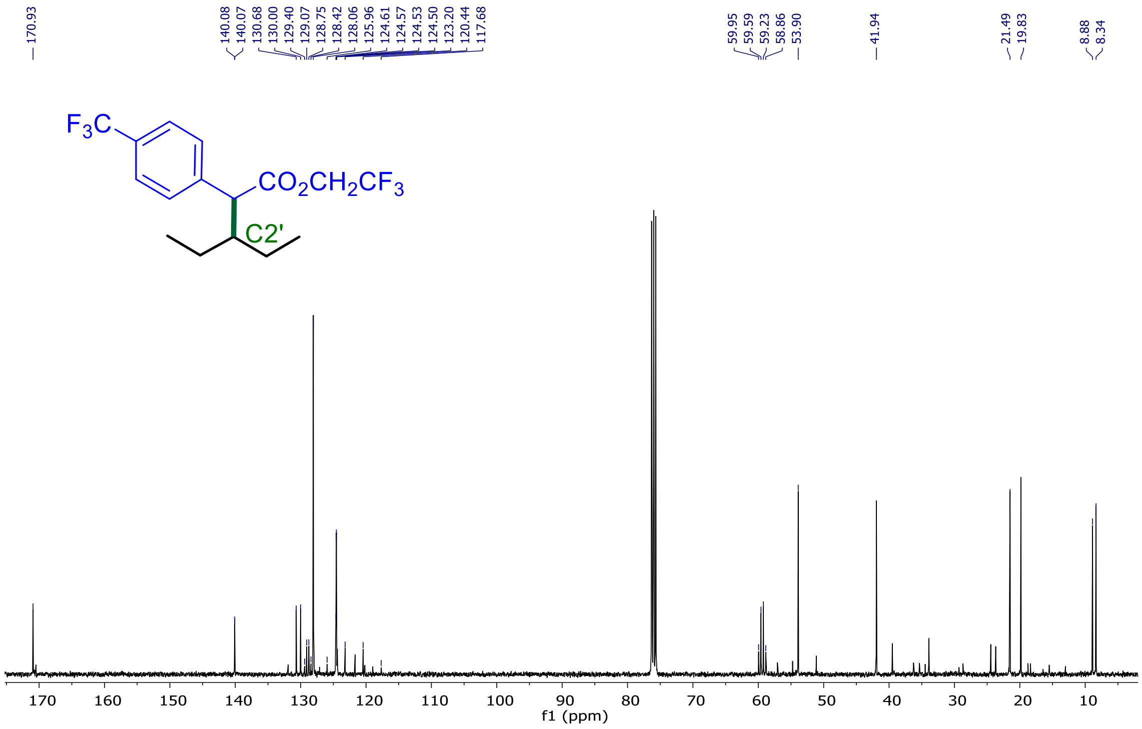

*Figure S37: $^{13}C\{^1H\}$ NMR spectrum of **2c** (100 MHz, CDCl$_3$).*

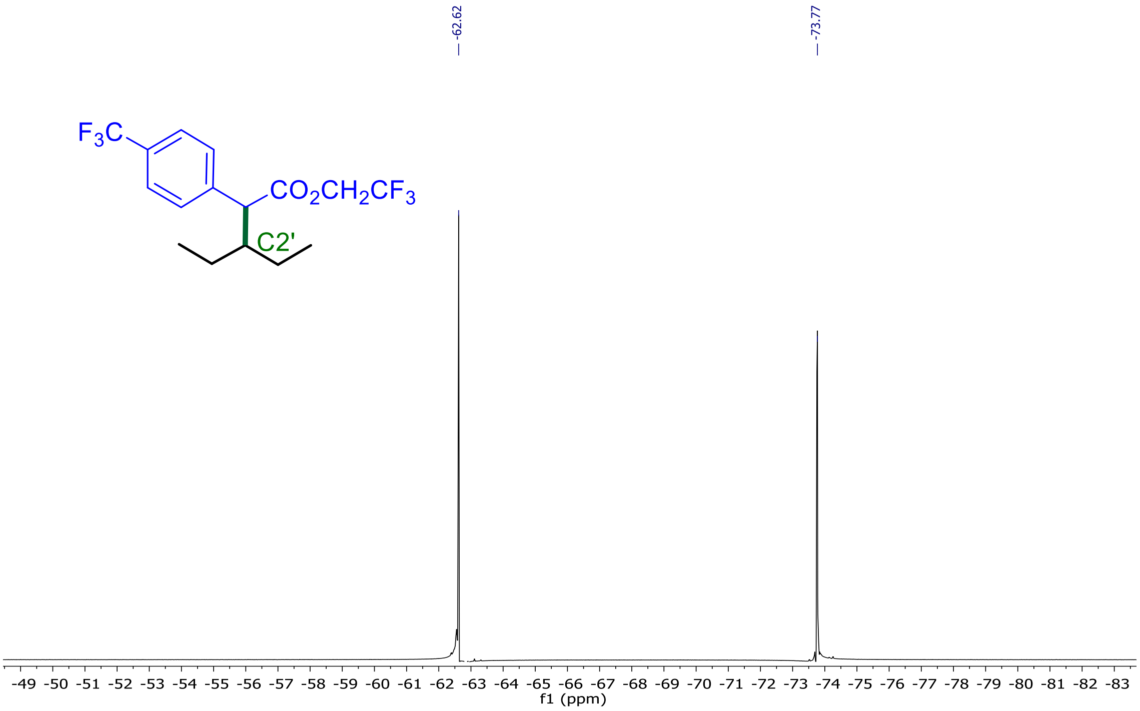

Figure S38:$^{19}F\{^{1}H\}$ *NMR spectrum of* ***2c*** *(375 MHz,* $CDCl_3$*).*

2,2,2-trifluoroethyl-2-(4-chlorophenyl)heptanoate 3a. 74% Yield. Impurified with **3b** and **3c**. ^{1}H *NMR* (400 MHz, $CDCl_3$): δ 7.24-7.16 (m, 4H), 4.44 (m, 1H, CH*H*CF_3), 4.28 (m, 1H, C*H*HCF_3), 3.54 (d, *J* = 11 Hz, 1H), 2.14 (m, 1H), 1.98 (m, 1H), 1.25 (m, 6H), 0.79 (m, 3H; $^{13}C\{^{1}H\}$ *NMR* (100 MHz, $CDCl_3$): δ 172.2, 136.5, 133.4, 129.9, 60.2, 50.6, 34.9, 33.27, 31.4, 26.9, 22.4, 13.9. $^{19}F\{^{1}H\}$ *NMR* (375 MHz, $CDCl_3$): δ -73.79.

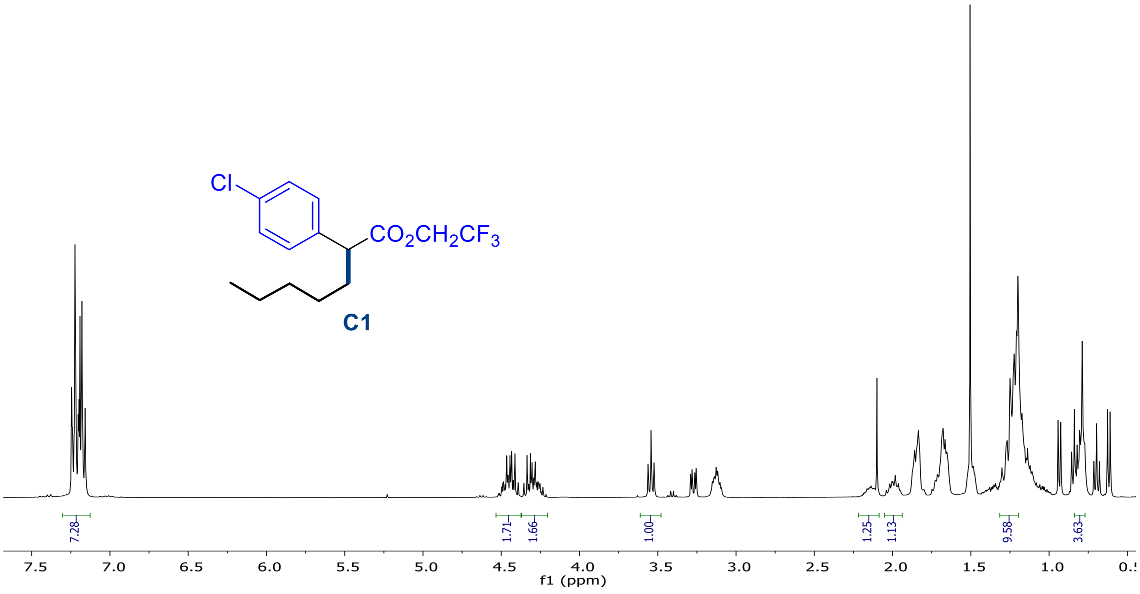

Figure S39: ^{1}H *NMR spectrum of* ***3a*** *(400 MHz,* $CDCl_3$*).*

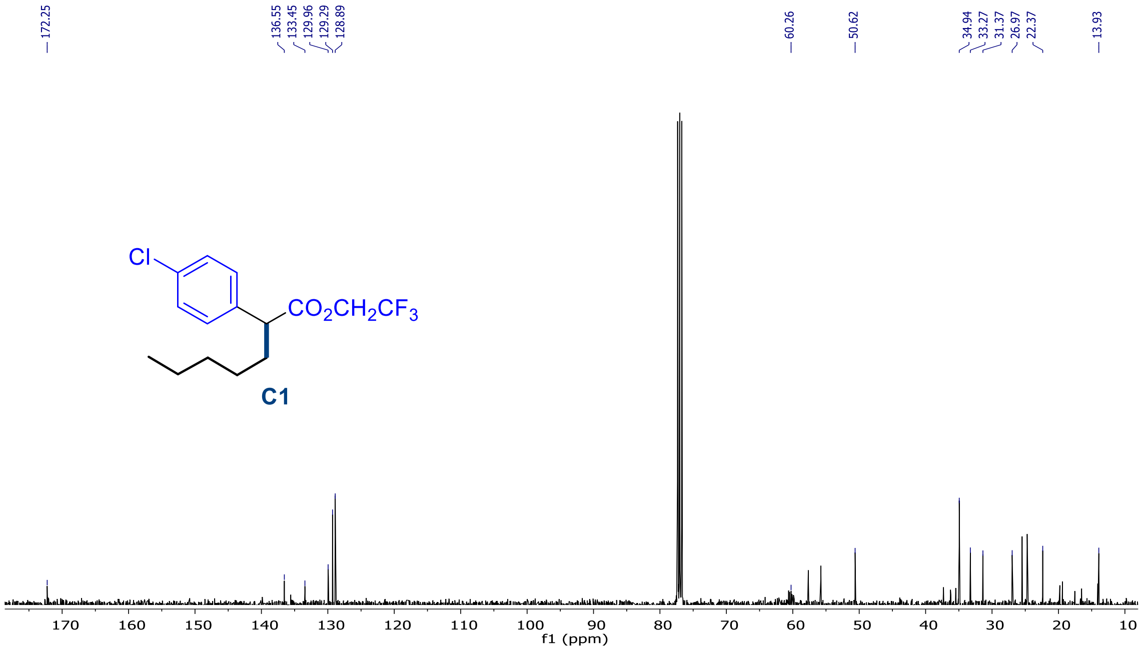

Figure S40: $^{13}C\{^1H\}$ NMR spectrum of **3a** (100 MHz, $CDCl_3$).

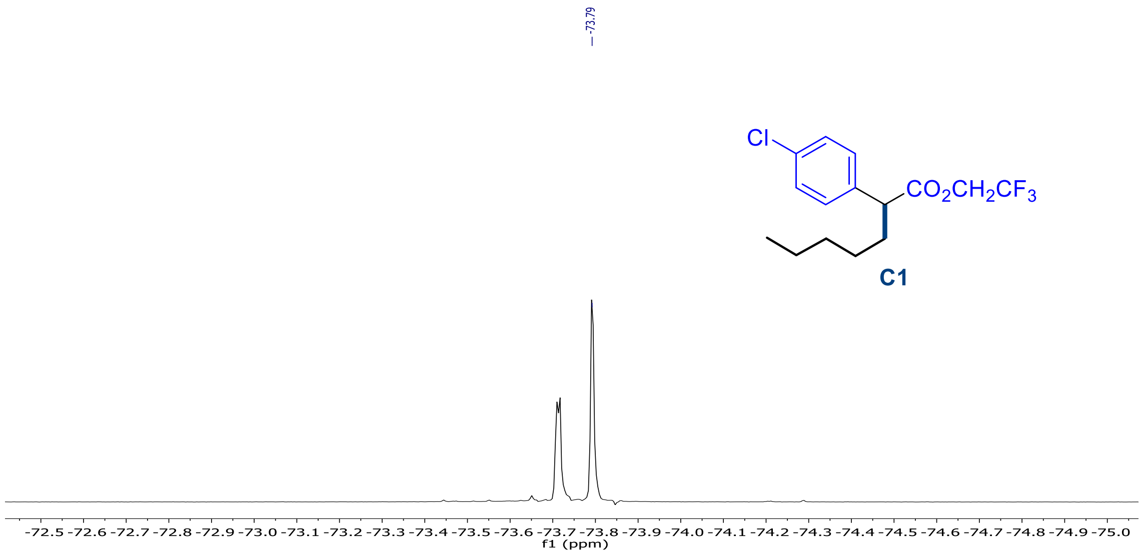

Figure S41: $^{19}F\{^1H\}$ NMR spectrum of **3a** (375 MHz, $CDCl_3$).

2,2,2-trifluoroethyl-2-(4-chlorophenyl)-3-methylhexanoate 3b. 92 % Yield, as a 45:55 mixture of diastereoisomers. Major isomer. ^1H *NMR* (400 MHz, $CDCl_3$): δ 7.24-7.18 (m, 4H), 4.46 (m, 1H, C*H*HCF_3), 4.26 (m, 1H, CH*H*CF_3), 3.26 (d, *J* =10.5 Hz, 1H), 2.14 (m, 1H), 1.47-1.07 (m, 4H), 0.83 (t, *J* = 6.5 Hz, 3H), 0.61 (d, *J* = 7.1 Hz, 3H). Minor isomer. ^1H *NMR* (400 MHz, $CDCl_3$): δ 7.24-7.18 (m, 4H), 4.46 (m, 1H, C*H*HCF_3), 4.26 (m, 1H, CH*H*CF_3), 3.26 (d, *J* =10.5 Hz, 1H), 2.14 (m, 1H), 1.47-1.07 (m, 4H), 0.94 (d, *J* = 7.1 Hz, 3H), 0.69 (t, *J* = 6.5 Hz, 3H). $^{13}C\{^1H\}$ *NMR* (100 MHz, $CDCl_3$): δ 171.1, 134.6, 132.5, 128.9, 127.8, 120.5 (q, CF_3, $J_{C\text{-}F}$ = 277 Hz), 59.5 (q, CH_2CF_3, $J_{C\text{-}F}$ = 36.7 Hz), 56.7, 36.3,

35.2, 35.1, 34.4, 18.8, 18.4, 16.5, 15.5, 13.1, 12.9. *$^{19}F\{^1H\}$ NMR* (375 MHz, $CDCl_3$): δ -73.73.

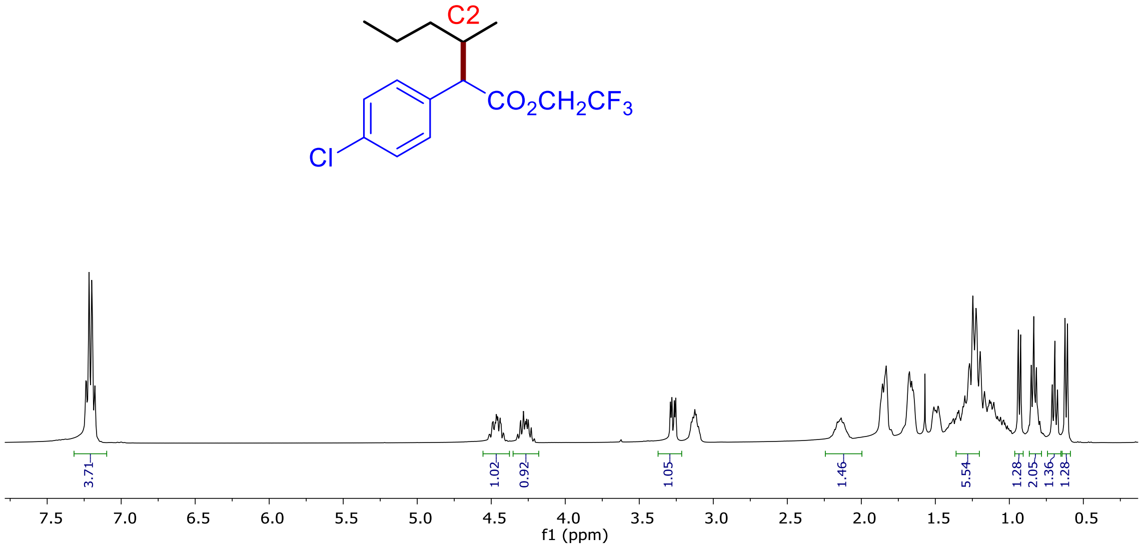

*Figure S42: ^{1}H NMR spectrum of **3b** (400 MHz, $CDCl_3$).*

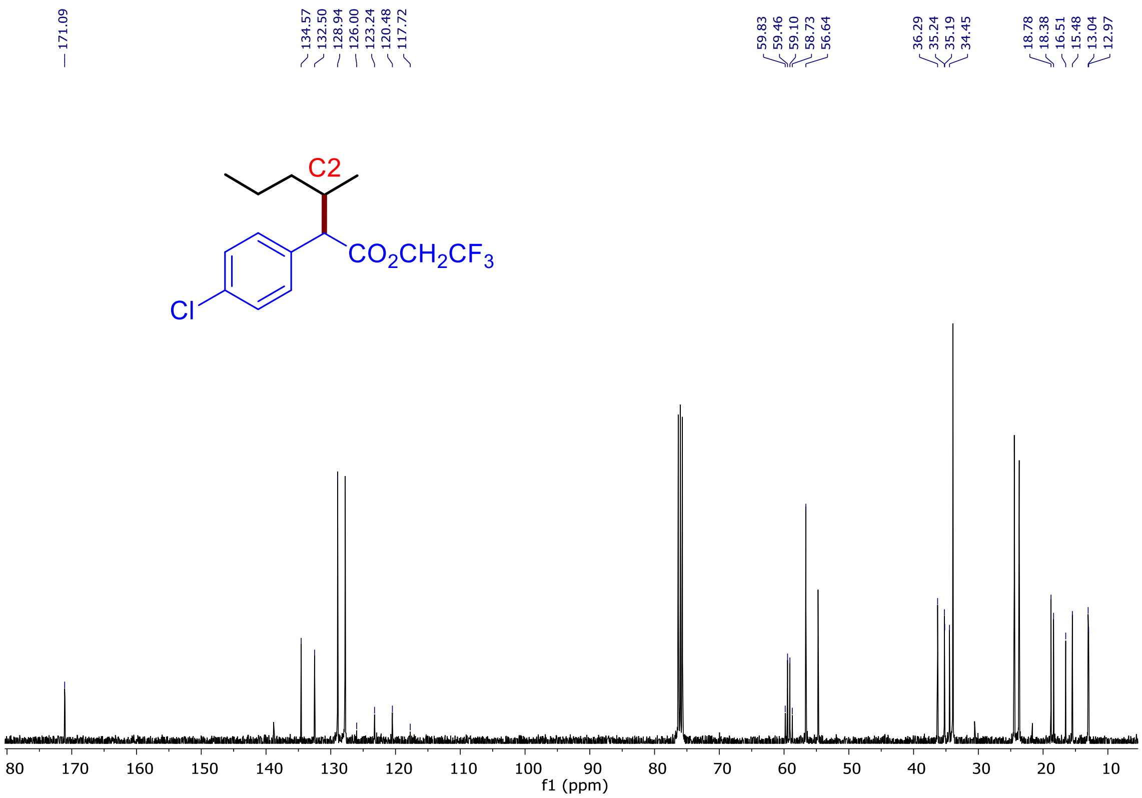

*Figure S43: $^{13}C\{^1H\}$ NMR spectrum of **3b** (100 MHz, $CDCl_3$).*

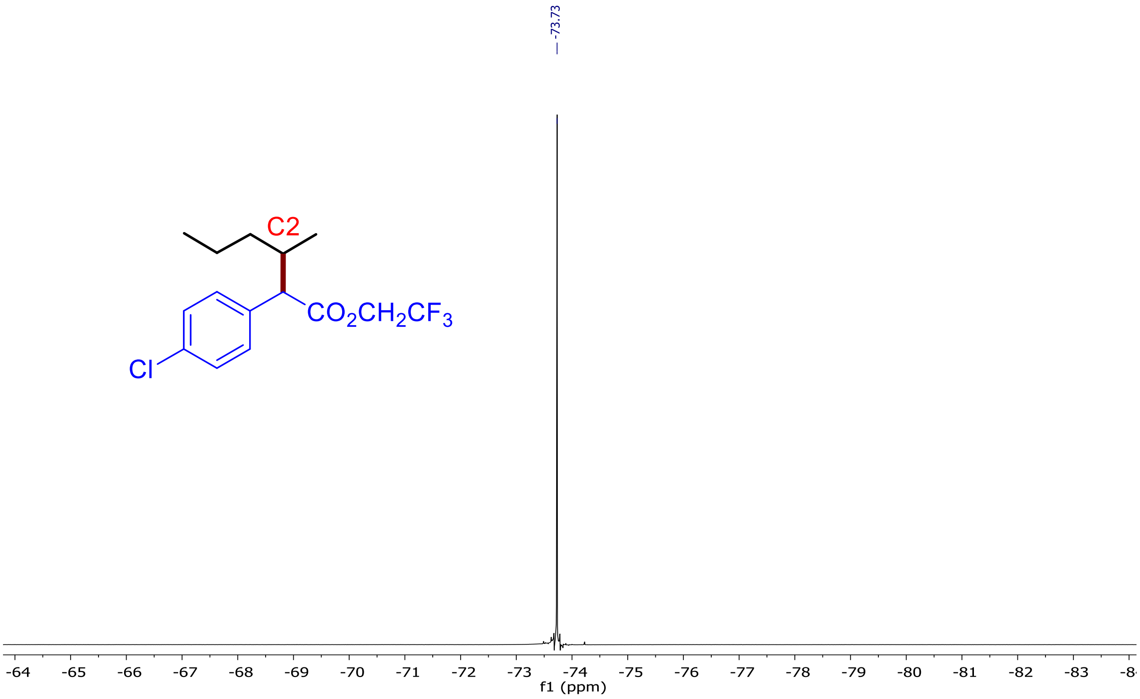

Figure S44: ^{19}*F{*^{1}*H} NMR spectrum of* ***3b*** *(375 MHz, $CDCl_3$).*

2,2,2-trifluoroethyl-2-(4-chlorophenyl)-3-ethylpentanoate 3c. 83% Yield ^{1}*H NMR* (400 MHz, $CDCl_3$): δ 7.22 (m, 4H), 4.52 (dq, 1H, $J_{H\text{-}H}$ = 12.7, $J_{H\text{-}F}$ = 8.4 Hz, CH*H*CF_3, CH*H*CF_3), 4.22 (dq, 1H, $J_{H\text{-}H}$ = 12.7, $J_{H\text{-}F}$ = 8.4 Hz, CH*H*CF_3, CH*H*CF_3), 3.45 (d, *J* = 11 Hz, 1H), 2.04 (m, 1H), 1.44-1.30 (m, 2H), 1.16 (m, 1H), 0.97 (m, 1H), 0.84 (t, *J* = 7.5 Hz, 3H), 0.65 (t, *J* = 7.5 Hz, 3H); ^{13}*C{*^{1}*H} NMR* (100 MHz, $CDCl_3$): δ172.3, 135.6, 133.5, 130.0, 128.8, 121.5 (q, *C*F_3, $J_{C\text{-}F}$ = 277 Hz), 60.5 (q, *C*H_2CF_3, $J_{C\text{-}F}$ = 36.5 Hz), 54.5, 42.8, 22.5, 20.8, 9.9, 9.4. ^{19}*F{*^{1}*H} NMR* (375 MHz, $CDCl_3$): δ -73.73.

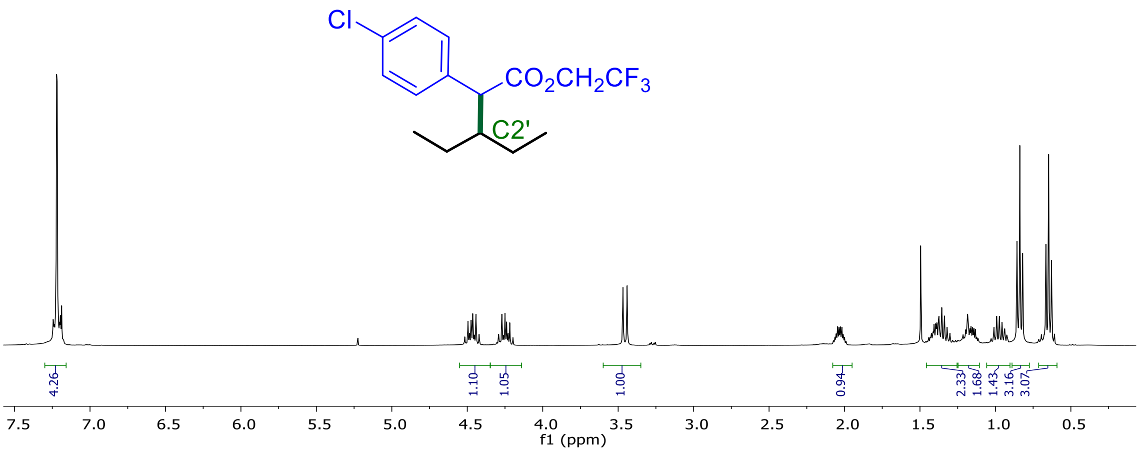

Figure S45: ^{1}*H NMR spectrum of* ***3c*** *(400 MHz, $CDCl_3$).*

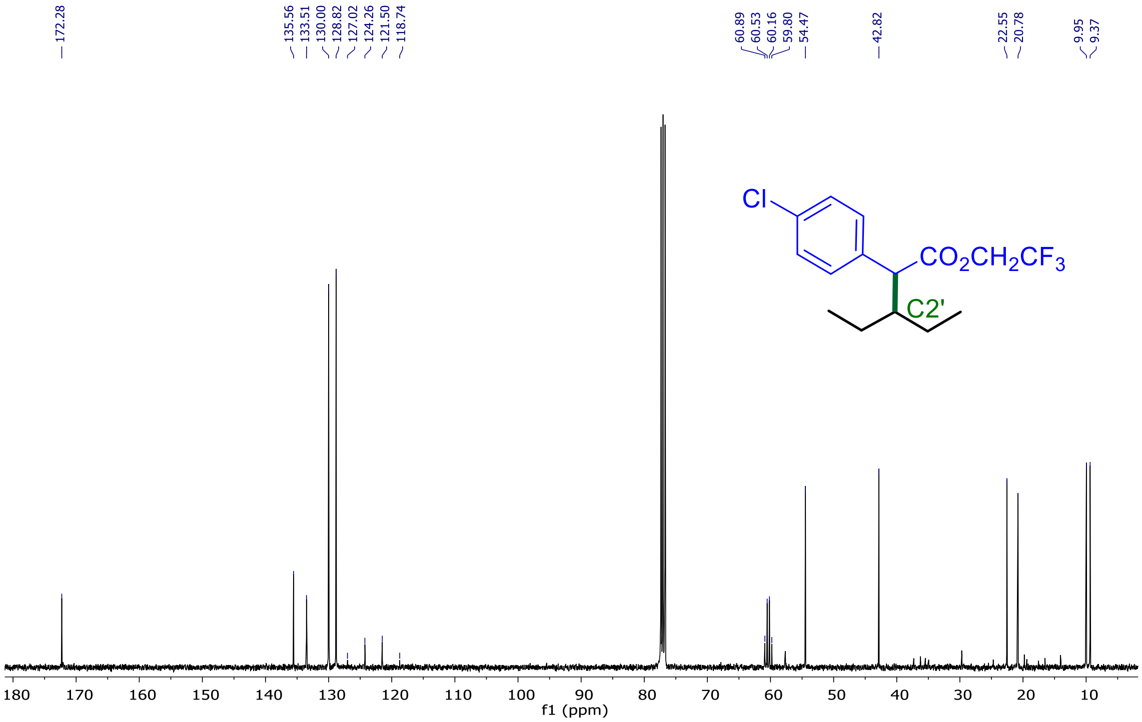

*Figure S46: $^{13}C\{^1H\}$ NMR spectrum of **3c** (100 MHz, $CDCl_3$).*

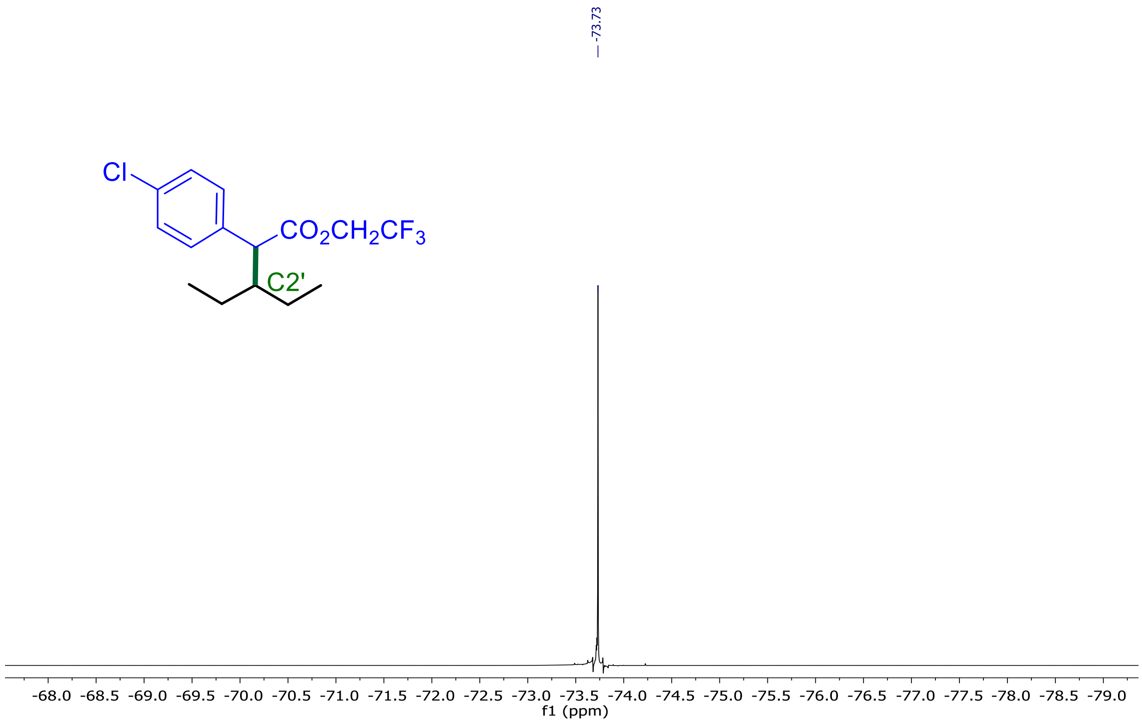

*Figure S47:$^{19}F\{^1H\}$ NMR spectrum of **3c** (375 MHz, $CDCl_3$).*

Ethyl 2-phenyloctanoate 4a. 99 % Yield. *^1H NMR* (400 MHz, $CDCl_3$): δ 7.31-7.22 (m, 4H), 4.12 (m, 2H), 3.51 (t, *J* =7.7 Hz, 1H), 2.06 (m, 1H), 1.75 (m, 1H), 1.27 (m, 8H), 1.21 (t, *J* = 7.1 Hz, 3H), 0.86 (m, 3H). *$^{13}C\{^1H\}$ NMR* (100 MHz, $CDCl_3$): δ 174.2, 139.5, 128.5, 127.9, 127.1, 60.6, 51.8, 33.6, 31.6, 29.0, 27.5, 22.6, 14.1, 14.0.

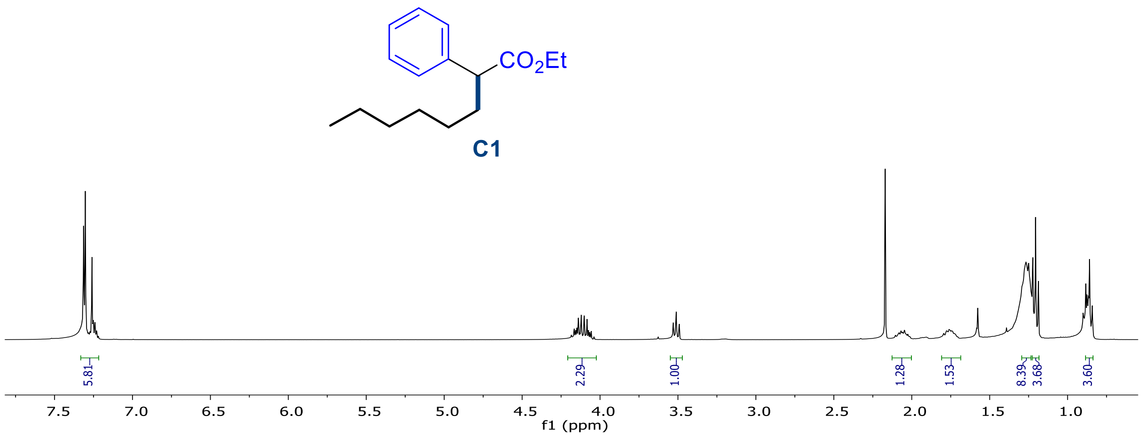

*Figure S48: ¹H NMR spectrum of **4a** (400 MHz, $CDCl_3$).*

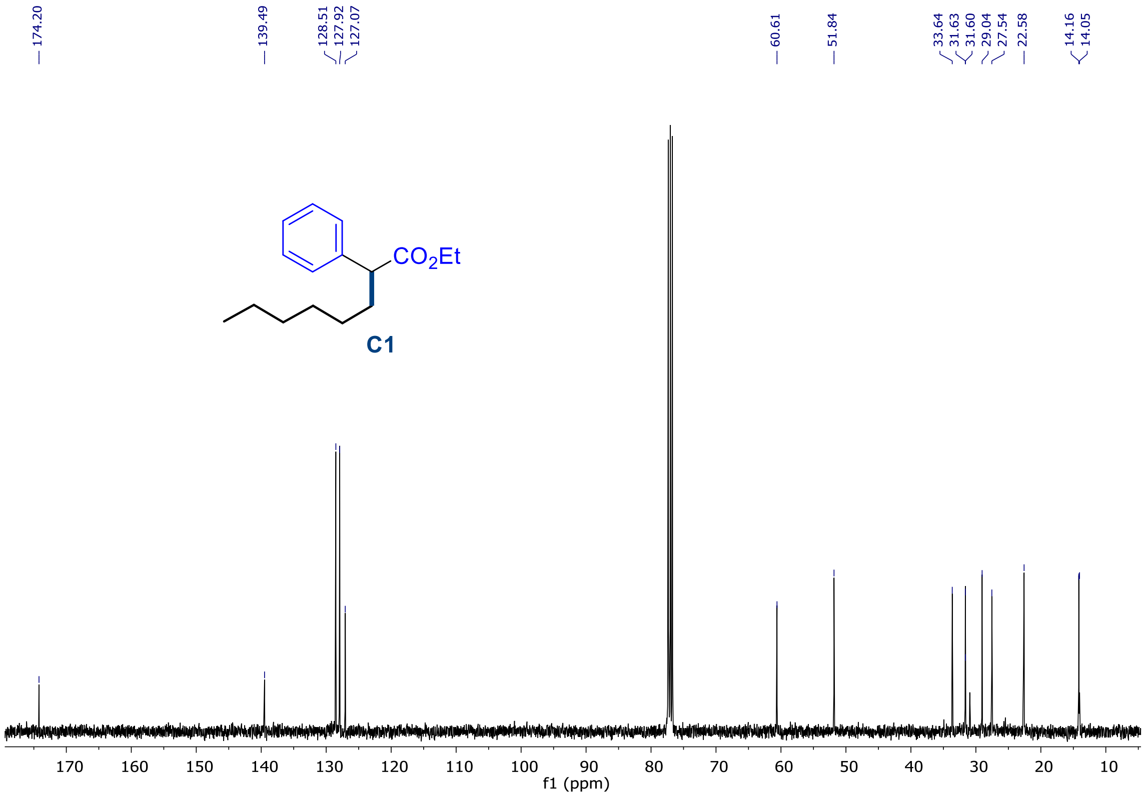

*Figure S49: $^{13}C\{^{1}H\}$ NMR spectrum of **4a** (100 MHz, $CDCl_3$).*

Methyl 2-(4-bromophenyl)octanoate 5a. 70 % Yield. *¹H NMR* (400 MHz, $CDCl_3$): δ 7.44 (d, *J* = 8.2 Hz, 2H), 7.18 (d, *J* = 8.2 Hz, 2H), 3.66 (s, 2H), 3.49 (t, *J* =7.7 Hz, 1H), 2.03 (m, 1H), 1.74 (m, 1H), 1.25 (m, 8H), 0.86 (t, *J* = 6.9 Hz, 3H). *$^{13}C\{^{1}H\}$ NMR* (100 MHz, $CDCl_3$): δ 173.2, 137.2, 130.6, 128.7, 120.1, 51.5, 50.5, 32.4, 30.5, 27.9, 26.4, 21.5, 13.0.

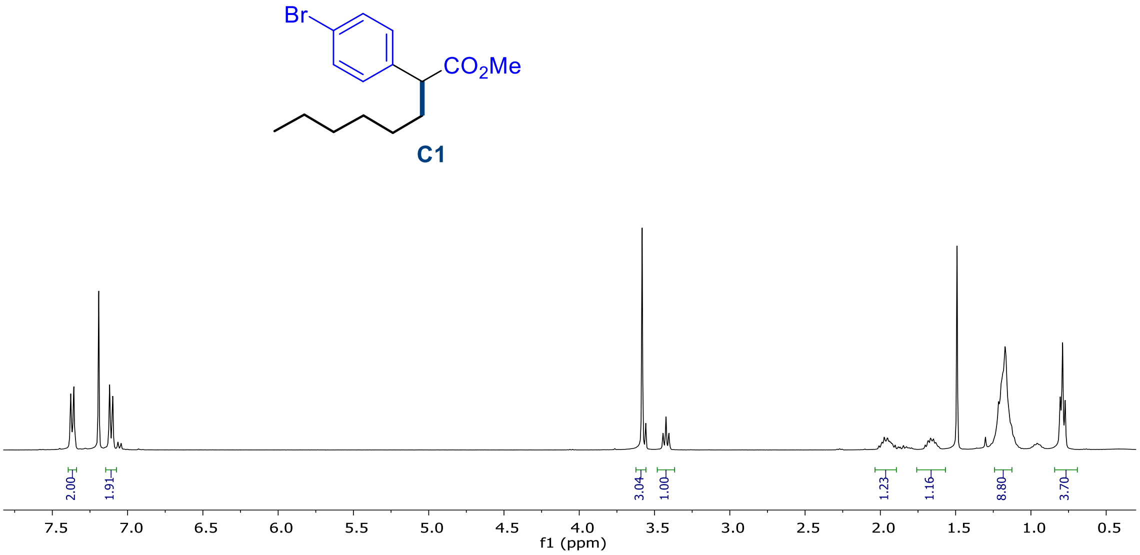

*Figure S50: ^{1}H NMR spectrum of **5a** (400 MHz, $CDCl_3$).*

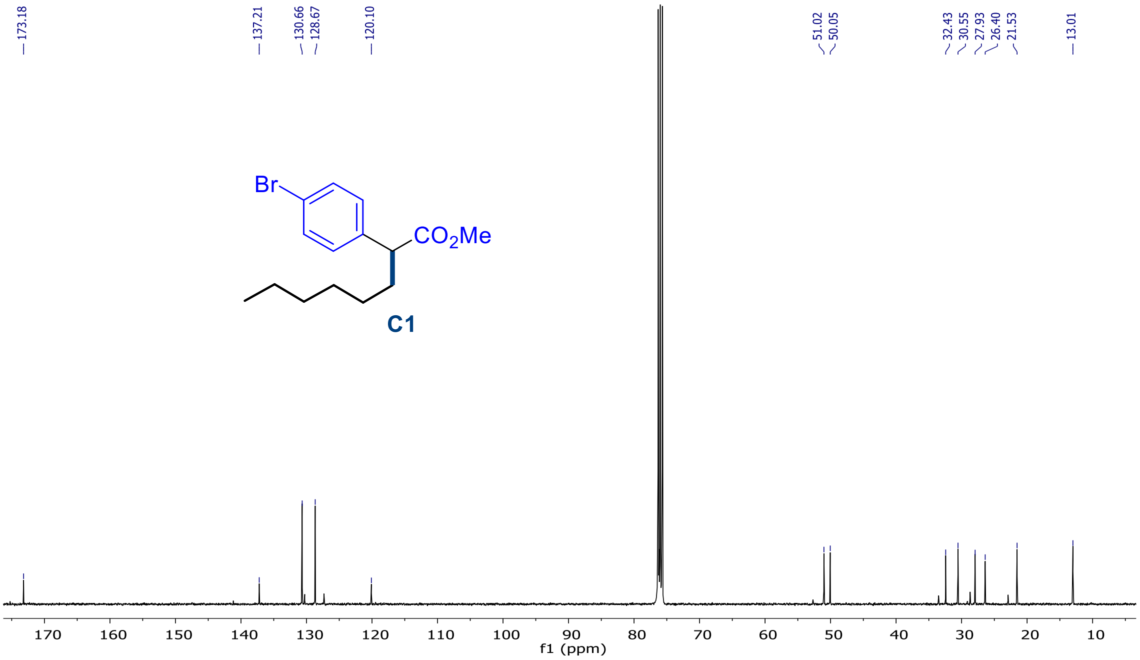

*Figure S51: $^{13}C\{^1H\}$ NMR spectrum of **5a** (100 MHz, $CDCl_3$).*

Mixture of methyl 2-(4-bromophenyl)-3-methylheptanoate (5b) and methyl 2-(4-bromophenyl)-3-ethylheptanoate (5c). 86 % Yield. Selected *^{1}H NMR* (400 MHz, $CDCl_3$): δ 3.41 (d, *J* = 10.5 Hz, 1H) **5c**; δ 3.24 (d, *J* = 11 Hz, 1H), 3.22 (d, *J* = 11 Hz, 1H) **5b**, 40:60 mixture of diastereoisomers.

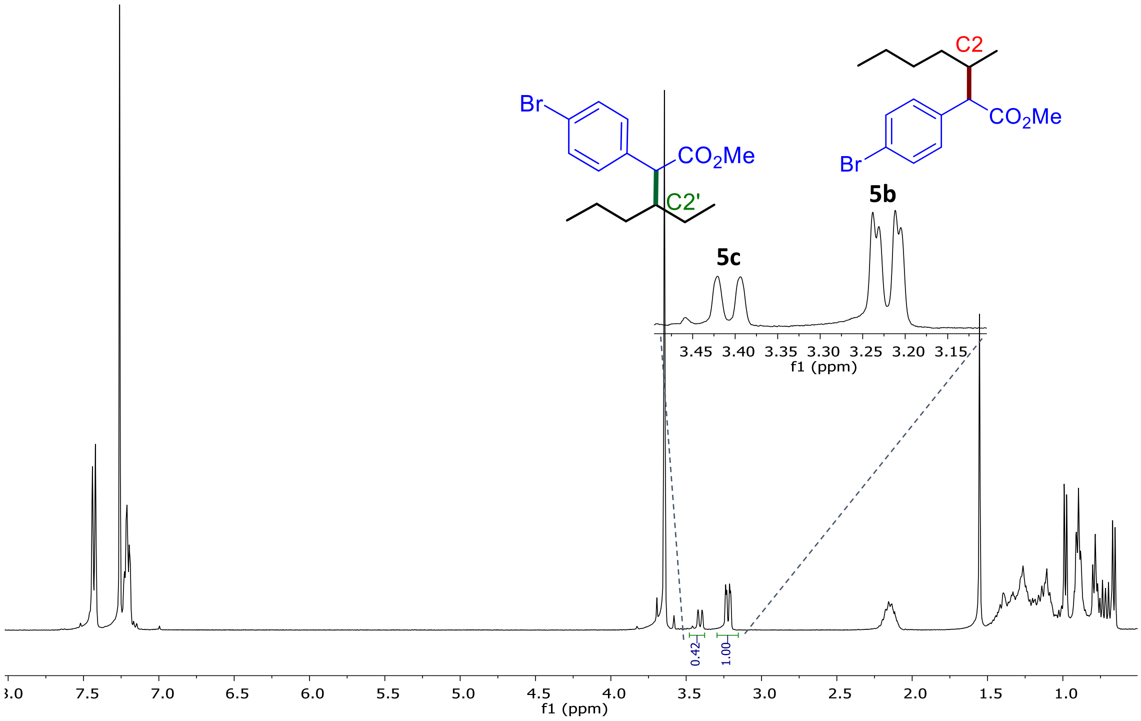

*Figure S52: ^{1}H NMR spectrum of **5b** and **5c** (400 MHz, $CDCl_3$).*

2,2,2-trifluoroethyl-2-(4-bromophenyl)octanoate 6a. 95 % Yield. *^{1}H NMR* (400 MHz, $CDCl_3$): δ 7.46 (d, *J* = 8.2 Hz, 2H), 7.18 (d, *J* = 8.2 Hz, 2H), 4.56 (dq, 1H, $J_{H\text{-}H}$ = 12.7, $J_{H\text{-}F}$ = 8.4 Hz, CH*H*CF_3, CH*H*CF_3), 4.32 (dq, 1H, $J_{H\text{-}H}$ = 12.7, $J_{H\text{-}F}$ = 8.4 Hz, CH*H*CF_3, CH*H*CF_3), 3.60 (t, *J* =7.7 Hz, 1H), 2.05 (m, 1H), 1.76 (m, 1H), 1.32-1.24 (m, 8H), 0.86 (t, *J* = 7.1 Hz, 3H). *^{13}C{^{1}H} NMR* (100 MHz, $CDCl_3$): δ 171.3, 136.1, 130.8, 128.6, 123.2 (q, *C*F_3, $J_{C\text{-}F}$ = 277 Hz), 59.6 (q, *C*H_2CF_3, $J_{C\text{-}F}$ = 36.5 Hz), 49.7, 32.3, 30.6, 27.8, 26.2, 21.5, 13.0. *^{19}F{^{1}H} NMR* (375 MHz, $CDCl_3$): δ -73.79.

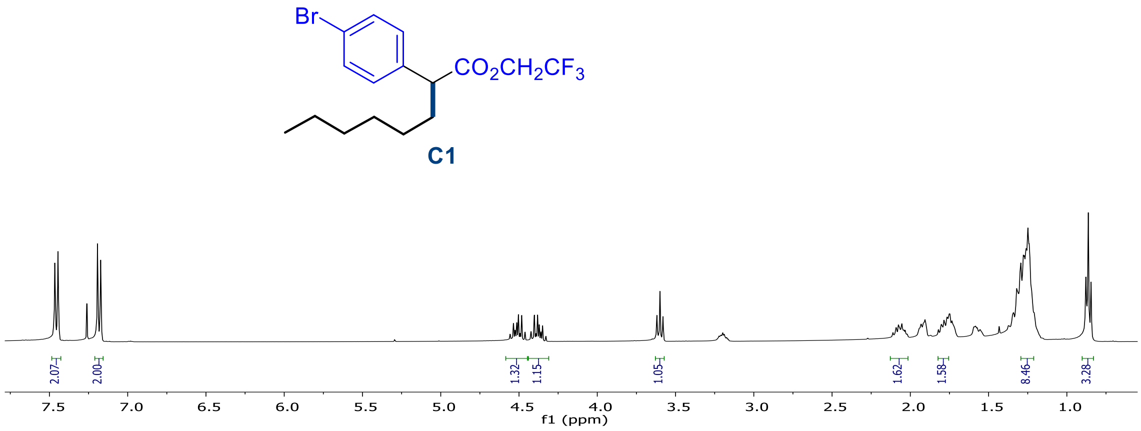

*Figure S53: ^{1}H NMR spectrum of **6a** (400 MHz, $CDCl_3$).*

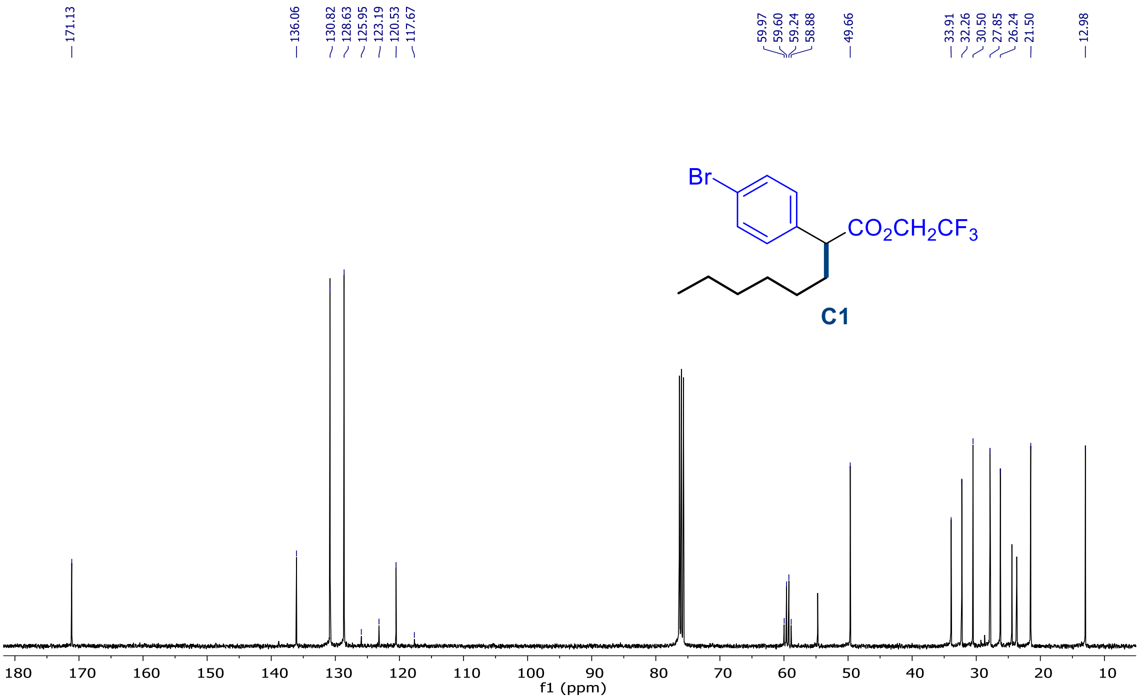

*Figure S54: $^{13}C\{^{1}H\}$ NMR spectrum of **6a** (100 MHz, $CDCl_3$).*

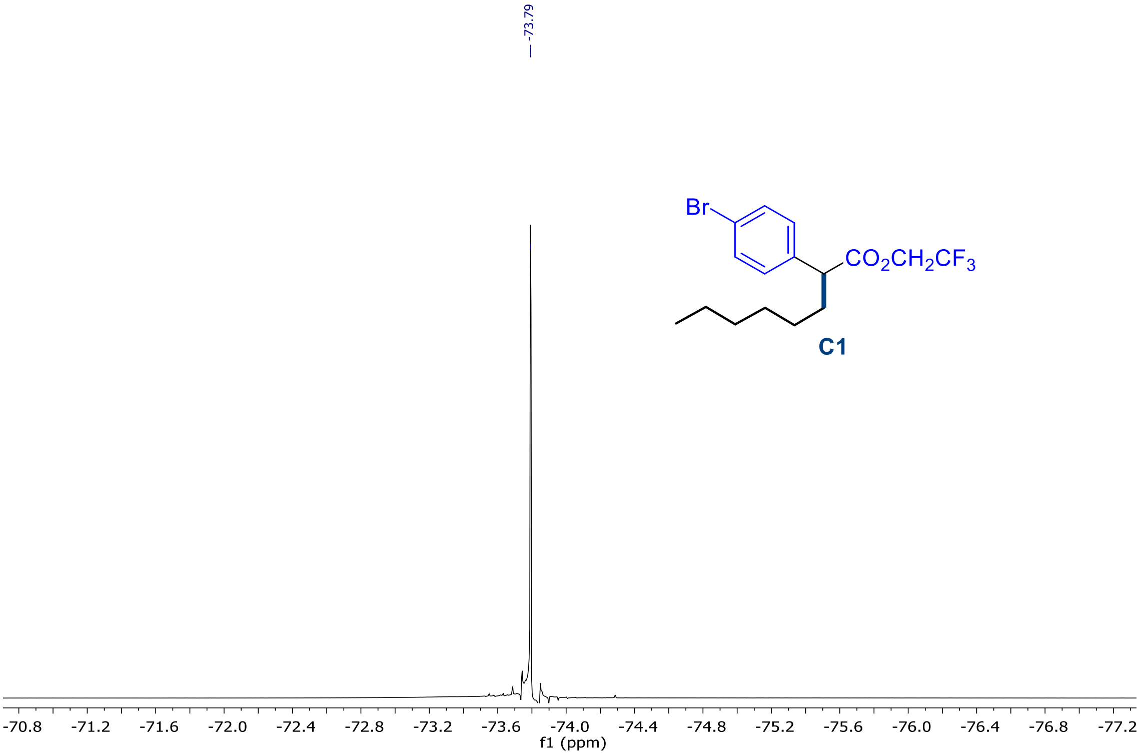

*Figure S55: $^{19}F\{^{1}H\}$ NMR spectrum of **6a** (375 MHz, $CDCl_3$).*

Mixture of 2,2,2-trifluoroethyl-2-(4-bromophenyl)-3-methylheptanoate (6b) and 2,2,2-trifluoroethyl-2-(4-bromophenyl)-3-ethylheptanoate (6c). 84 % Yield. Selected ^{1}H *NMR* (400 MHz, $CDCl_3$): δ 3.44 (d, J = 10.5 Hz, 1H), 3.45 (d, J = 10.5 Hz, 1H) **6c**; δ 3.27 (d, J = 11 Hz, 1H), 3.26

(d, J = 11 Hz, 1H) **6b**, 40:60 mixture of diastereoisomers. $^{19}F\{^{1}H\}$ *NMR* (375 MHz, $CDCl_3$): δ -73.72 **6c**; δ -73.71 **6b**.

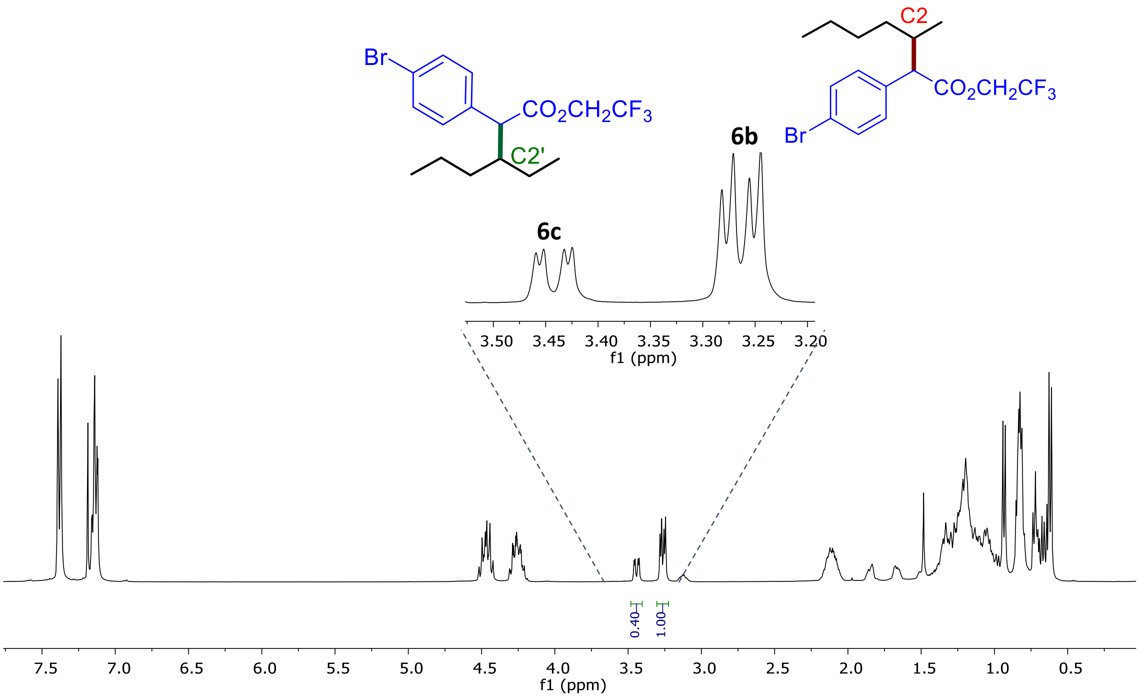

*Figure S56: ^{1}H NMR spectrum of **6b** and **6c** (400 MHz, $CDCl_3$).*

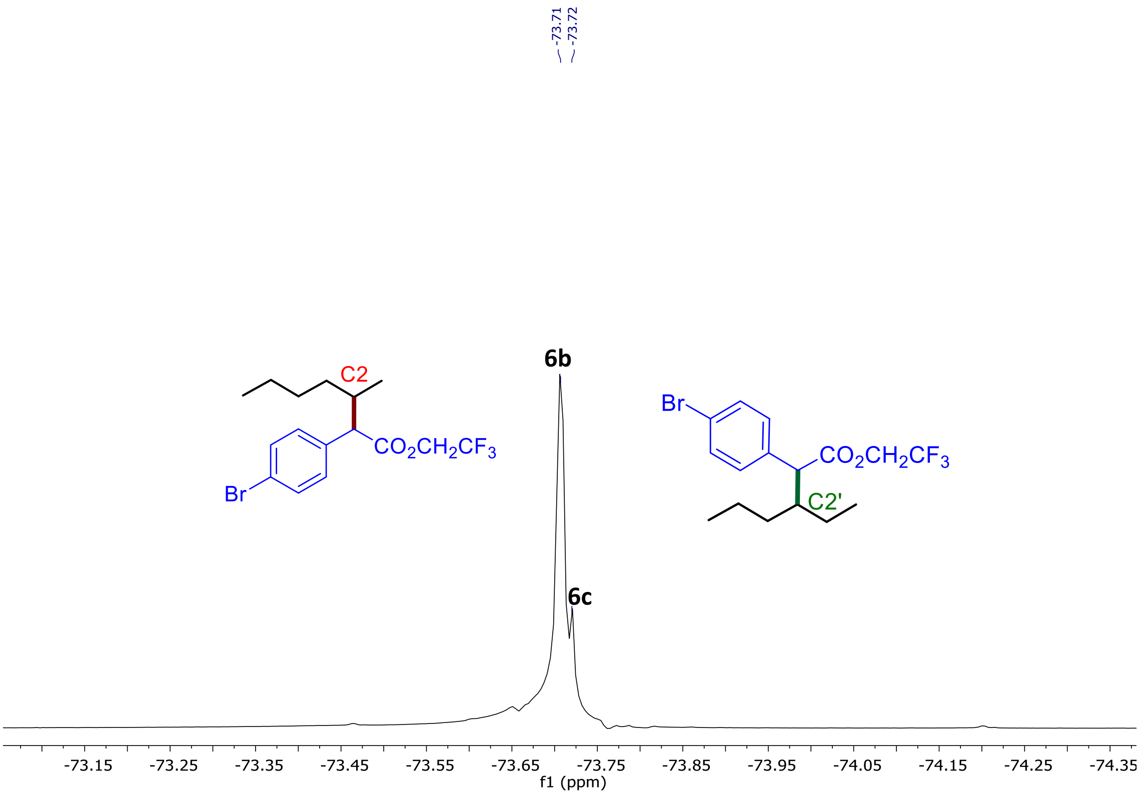

*Figure S7: $^{19}F\{^{1}H\}$ NMR spectrum of **6b** and **6c** (375 MHz, $CDCl_3$).*

2,2,2-trichloroethyl-2-(4-bromophenyl)octanoate 7a. 92 % Yield. *^{1}H NMR* (400 MHz, $CDCl_3$): δ 7.45 (d, *J* = 8.4 Hz, 2H), 7.22 (d, *J* = 8.4 Hz, 2H), 4.70 (q, *J* = 12 Hz, 2H), 3.64 (t, *J* =7.7 Hz, 1H), 2.13 (m, 1H), 1.81 (m, 1H), 1.32-1.24 (m, 8H), 0.86 (t, *J* = 7 Hz, 3H). *$^{13}C\{^{1}H\}$ NMR* (100 MHz, $CDCl_3$): δ 171.0, 136.1, 130.7, 128.8, 120.4, 93.8, 73.0, 50.0, 32.1, 30.5, 27.9, 26.3, 21.5, 13.0.

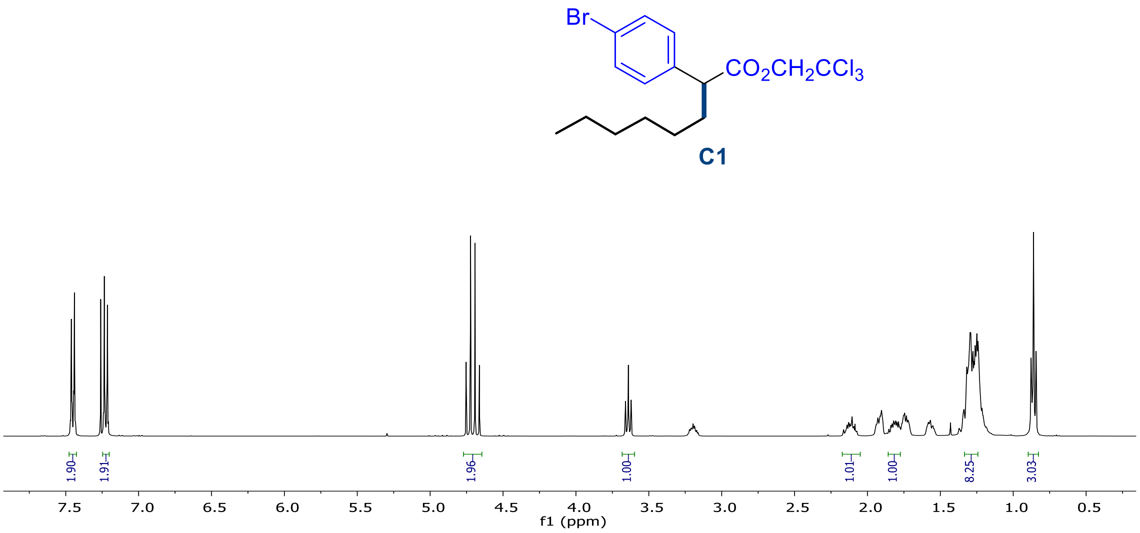

*Figure S58: ^{1}H NMR spectrum of **7a** (400 MHz, $CDCl_3$).*

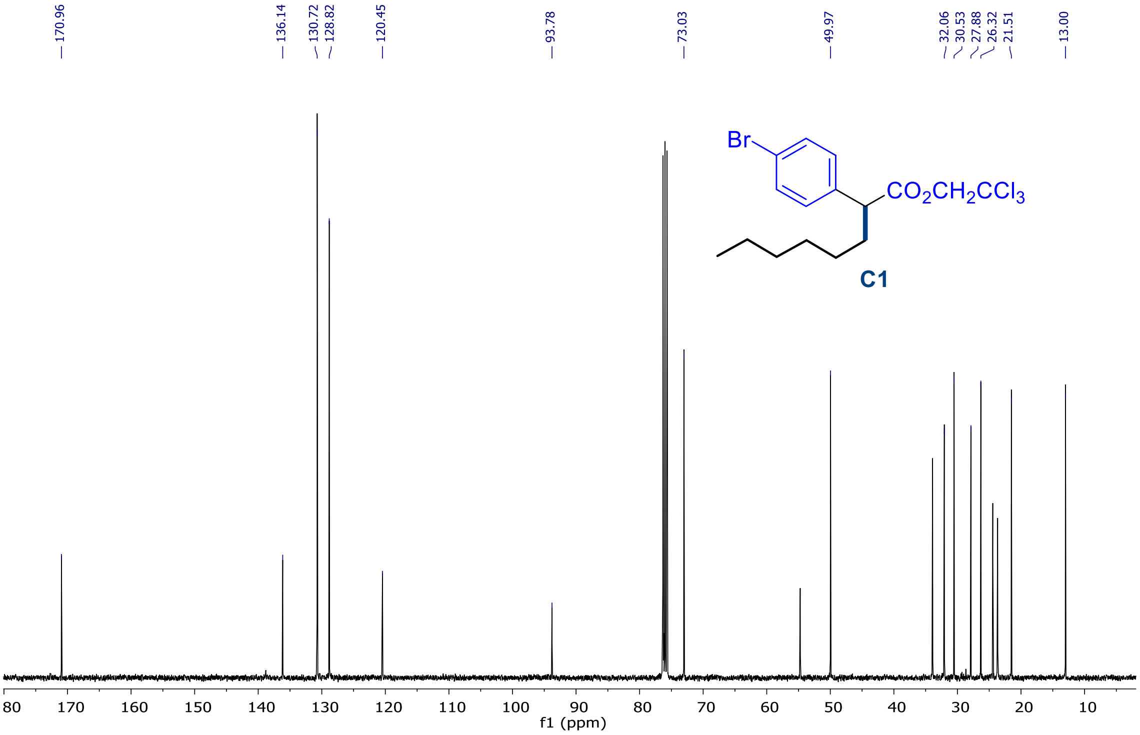

*Figure S59: $^{13}C\{^{1}H\}$ NMR spectrum of **7a** (100 MHz, $CDCl_3$).*

Mixture of 2,2,2-trichlororoethyl-2-(4-bromophenyl)-3-methylheptanoate (7b) and 2,2,2-trichloroethyl-2-(4-bromophenyl)-3-ethylheptanoate (7c). 87% Yield. Selected *^{1}H NMR* (400

MHz, $CDCl_3$): δ 3.48 (d, J = 11 Hz, 1H), 3.49 (d, J = 11 Hz, 1H) **7c**; δ 3.33 (d, J = 10.6 Hz, 1H), 3.32 (d, J = 10.6 Hz, 1H) **7b**, 50:50 mixture of diastereoisomers.

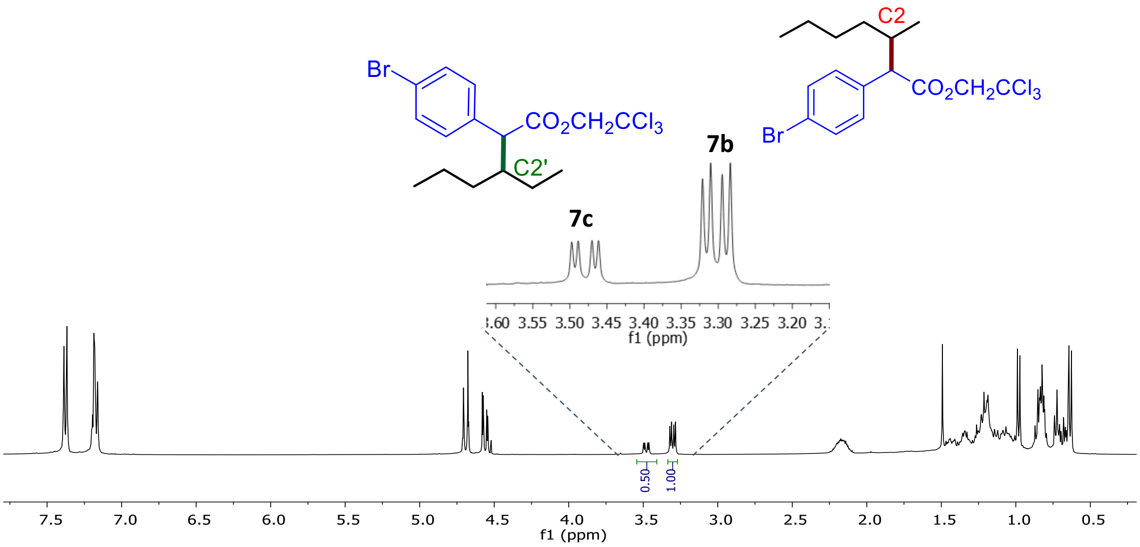

*Figure S60: ^{1}H NMR spectrum of **7b** and **7c** (400 MHz, $CDCl_3$).*

2,2,2-trifluoroethyl-2-(4-chlorophenyl)octanoate 8a. 95 % Yield. *^{1}H NMR* (400 MHz, $CDCl_3$): δ 7.46 (d, J = 8.2 Hz, 2H), 7.18 (d, J = 8.2 Hz, 2H), 4.41 (dq, 1H, J_{H-H} = 12.7, J_{H-F} = 8.4 Hz, CH*H*CF_3, CH*H*CF_3), 4.31 (dq, 1H, J_{H-H} = 12.7, J_{H-F} = 8.4 Hz, CH*H*CF_3, CH*H*CF_3), 3.60 (t, J =7.7 Hz, 1H), 2.05 (m, 1H), 1.76 (m, 1H), 1.32-1.24 (m, 8H), 0.86 (t, J = 7.1 Hz, 3H). *$^{13}C\{^{1}H\}$ NMR* (100 MHz, $CDCl_3$): δ 171.2, 135.6, 132.4, 128.6, 123.2 (q, CF_3, J_{C-F} = 277 Hz), 59.5 (q, CH_2CF_3, J_{C-F} = 36.5 Hz), 49.6, 32.3, 30.5, 27.8, 26.2, 21.5, 12.9. *$^{19}F\{^{1}H\}$ NMR* (375 MHz, $CDCl_3$): δ -73.78.

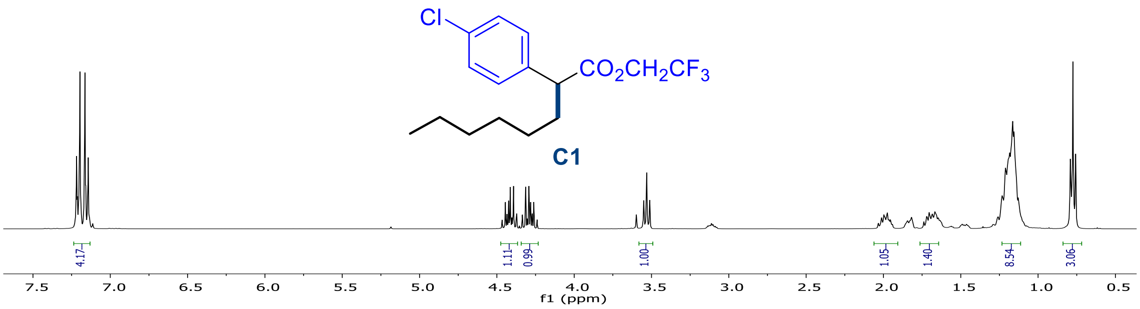

*Figure S61: ^{1}H NMR spectrum of **8a** (400 MHz, $CDCl_3$).*

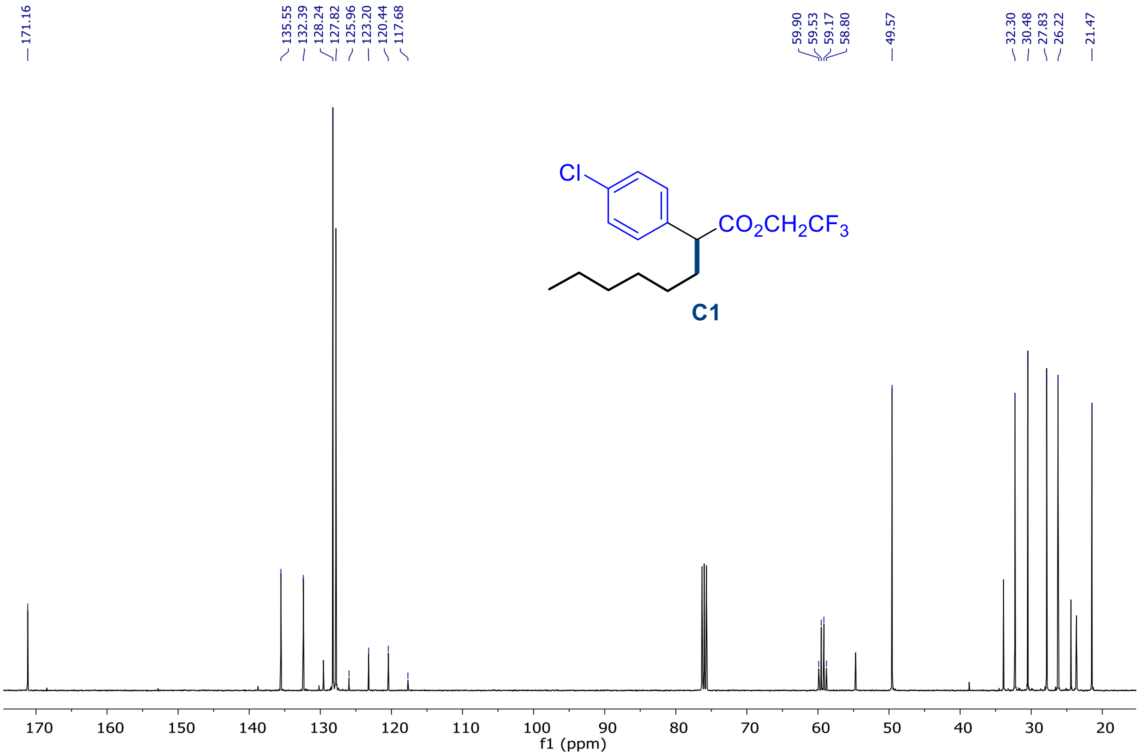

*Figure S62: $^{13}C\{^1H\}$ NMR spectrum of **8a** (100 MHz, $CDCl_3$).*

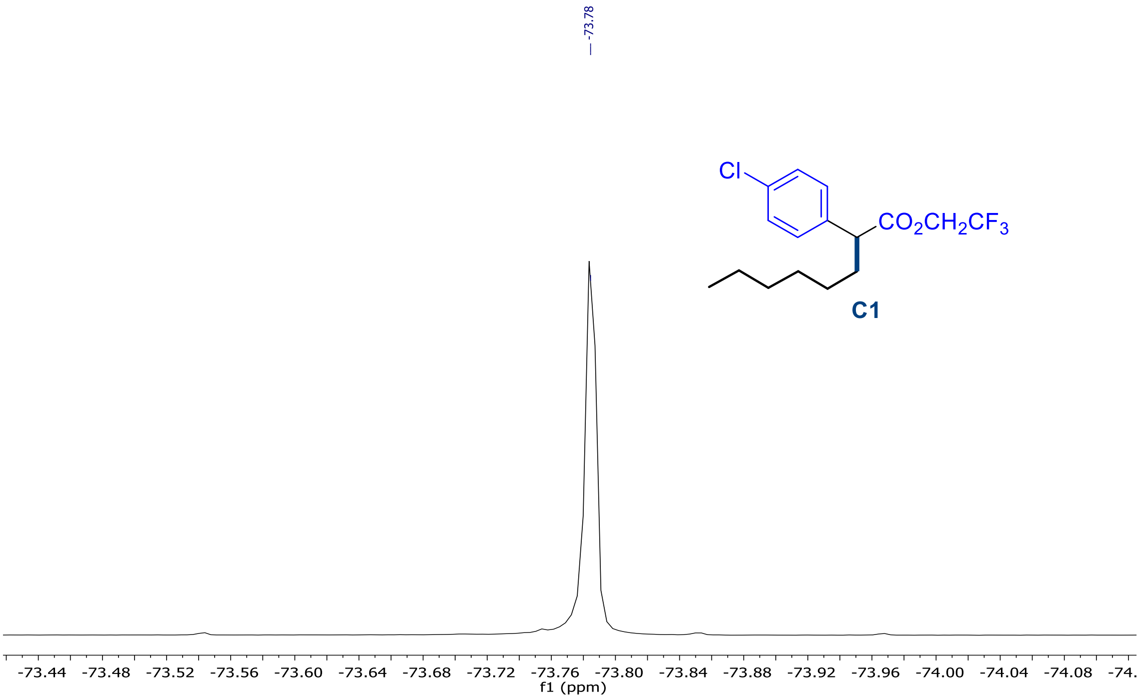

*Figure S63:$^{19}F\{^1H\}$ NMR spectrum of **8a** (375 MHz, $CDCl_3$).*

Mixture of 2,2,2-trifluoroethyl-2-(4-chlorophenyl)-3-methylheptanoate (8b) and 2,2,2-trifluoroethyl-2-(4-chlorophenyl)-3-ethylheptanoate (8c). 92% Yield. Selected ^1H *NMR* (400 MHz, $CDCl_3$): δ 3.48 (d, *J* = 10.5 Hz, 1H), 3.47 (d, *J* = 10.5 Hz, 1H) **8c**; δ 3.28 (d, *J* = 10.9 Hz, 1H),

3.27 (d, J = 10.9 Hz, 1H) **8b**, 40:60 mixture of diastereoisomers. *$^{19}F\{^1H\}$ NMR* (375 MHz, $CDCl_3$): δ -73.72 **8c**; δ -73.71 **8b**.

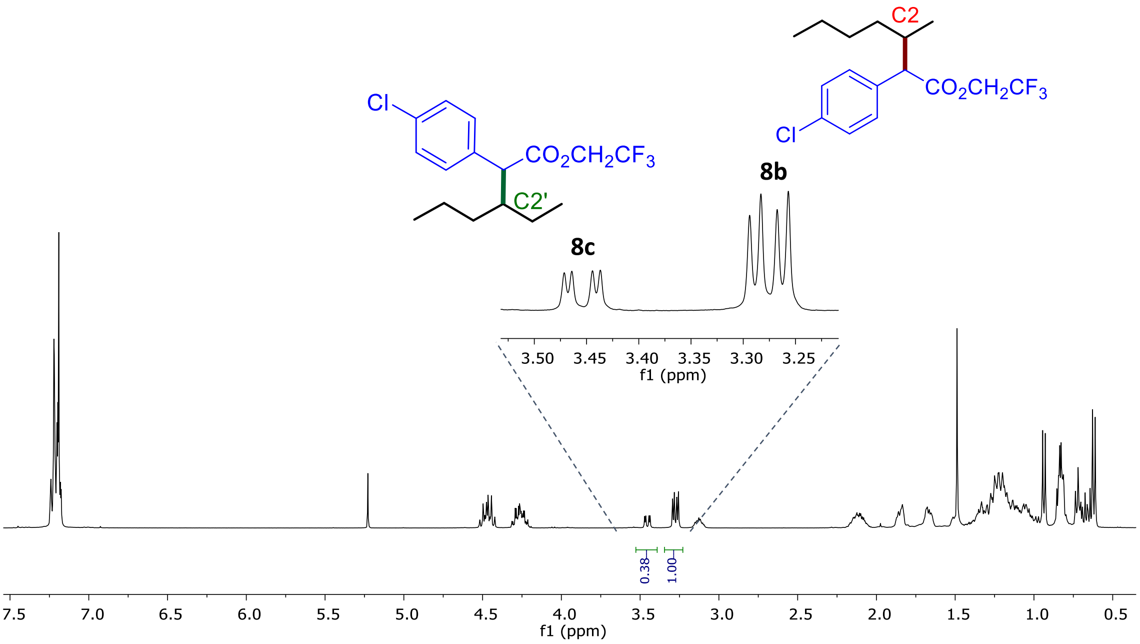

*Figure S64: ^{1}H NMR spectrum of **8b** and **8c** (400 MHz, $CDCl_3$).*

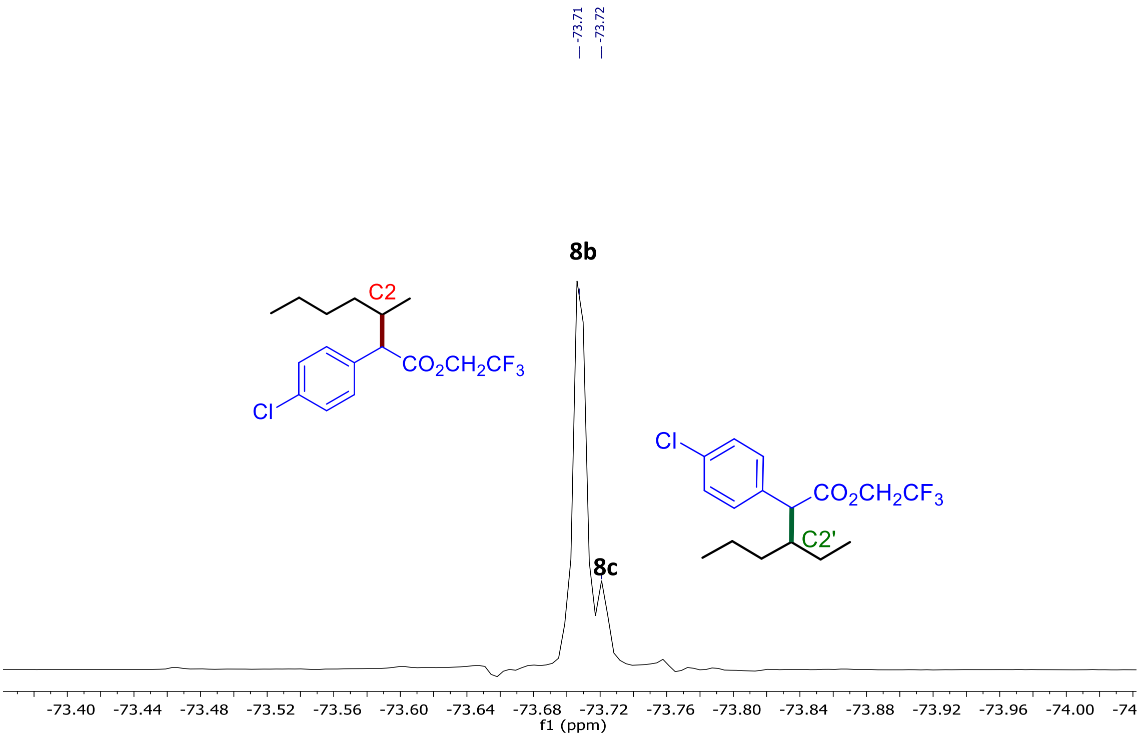

*Figure S65: $^{19}F\{^1H\}$ NMR spectrum of **8b** and **8c** (375 MHz, $CDCl_3$).*

2,2,2-trifluoroethyl-2-(4-trifluorophenyl)octanoate 9a. 54 % Yield. ^{1}H *NMR* (400 MHz, $CDCl_3$): δ 7.46 (d, *J* = 8.2 Hz, 2H), 7.18 (d, *J* = 8.2 Hz, 2H),4.56 (m, 1H, CH*H*CF_3), 4.32 (m, 1H, C*H*HCF_3), 3.60 (t, *J* =7.7 Hz, 1H), 2.05 (m, 1H), 1.76 (m, 1H), 1.32-1.24 (m, 8H), 0.86 (t, *J* = 7.1 Hz, 3H). $^{19}F\{^{1}H\}$ *NMR* (375 MHz, $CDCl_3$): δ -62.61, -73.80.

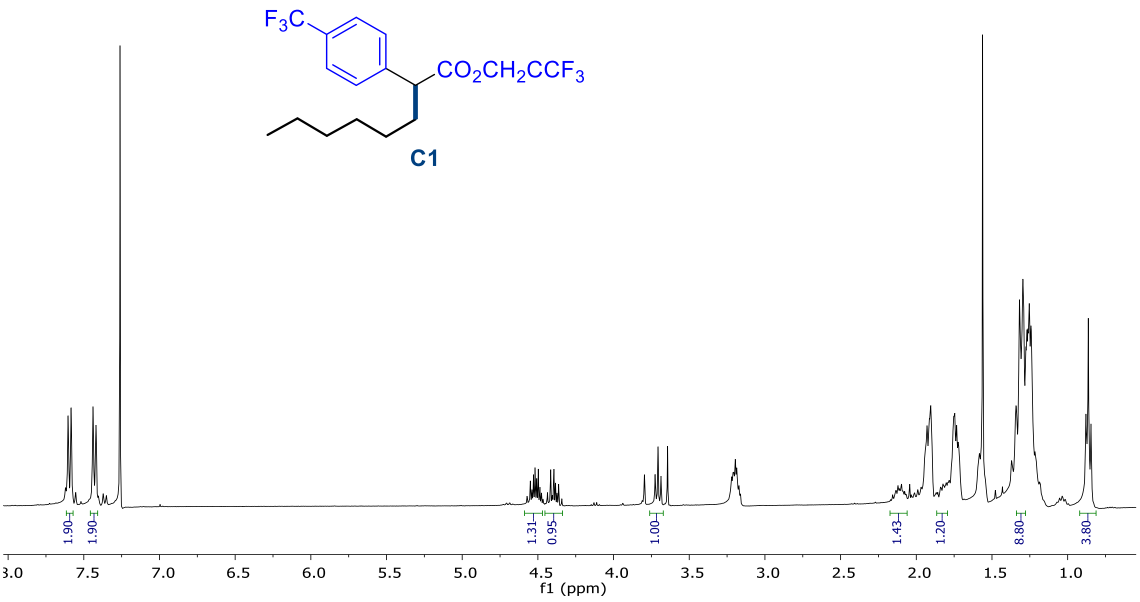

*Figure S66: ^{1}H NMR spectrum of **9a** (400 MHz, $CDCl_3$).*

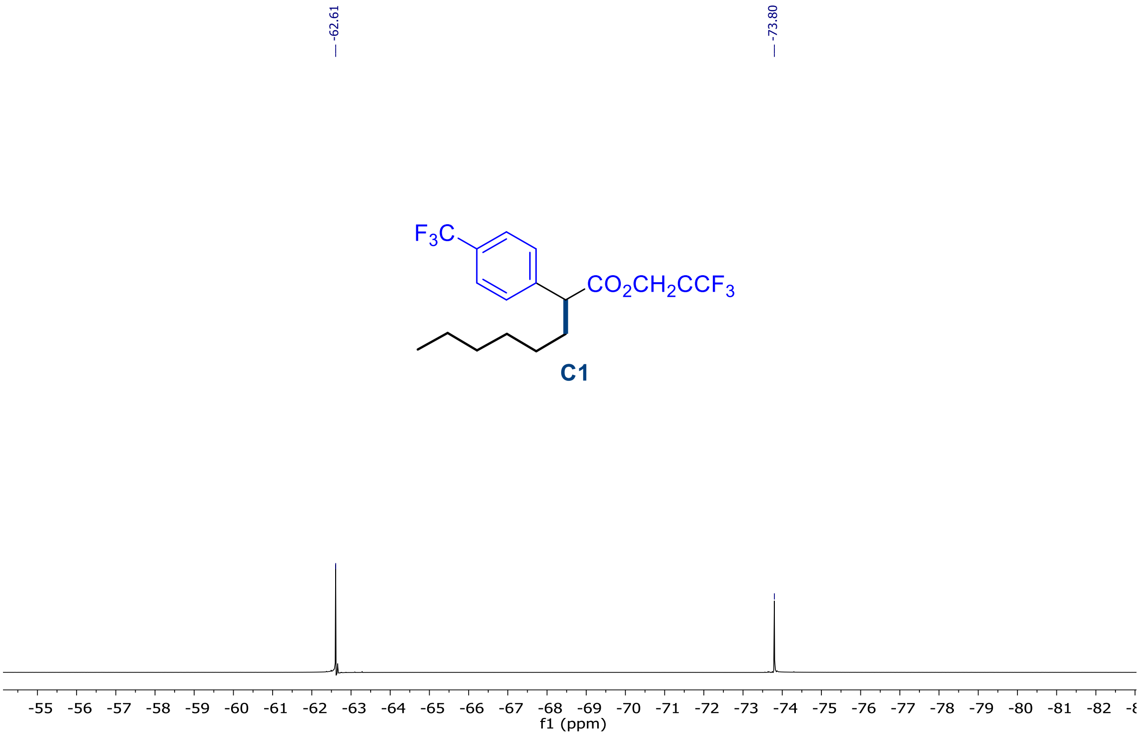

*Figure S67: $^{19}F\{^{1}H\}$ NMR spectrum of **9a** (375 MHz, $CDCl_3$).*

Mixture of 2,2,2-trifluoroethyl-2-(4-trifluorophenyl)-3-methylheptanoate (9b) and 2,2,2-trifluoroethyl-2-(4-trifluorophenyl)-3-ethylheptanoate (9c). 82% Yield. Selected ^{1}H *NMR* (400 MHz, $CDCl_3$): δ 3.55 (d, *J* = 10.2 Hz, 1H), 3.54 (d, *J* = 10.2 Hz, 1H) **9c**; δ 3.37 (d, *J* = 10.5 Hz, 1H), 3.36 (d, *J* = 10.5 Hz, 1H) **9b**, 45:55 mixture of diastereoisomers.^{19}F *{^{1}H} NMR* (375 MHz, $CDCl_3$): δ -73.82 **8b**,-73.80 **8c**; -62.68 **8b** ,-62.67 **8c**.

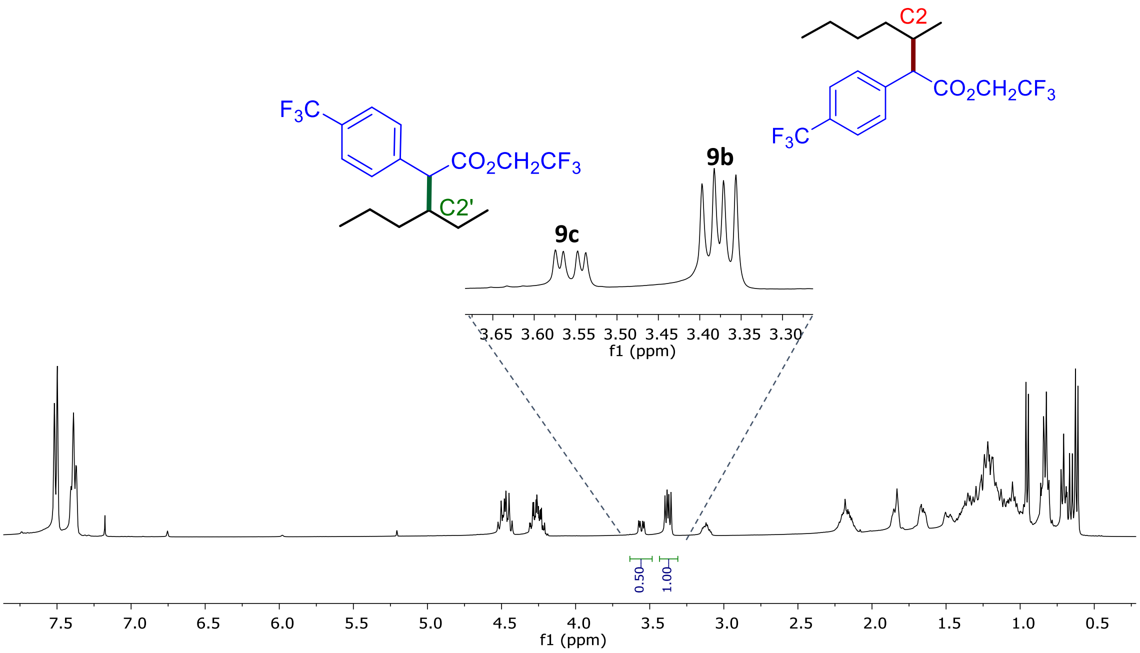

*Figure S69: ^{1}H NMR spectrum of **9b** and **9c** (400 MHz, $CDCl_3$).*

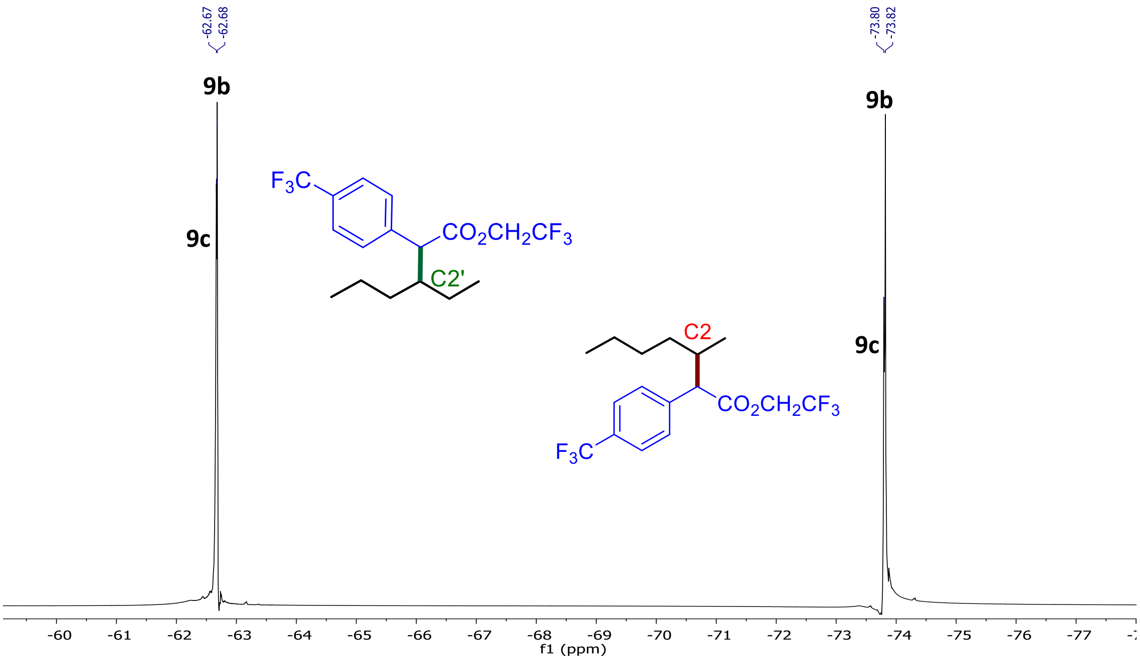

*Figure S70:$^{19}F\{^{1}H\}$ NMR spectrum of **9b** and **9c** (375 MHz, $CDCl_3$).*

5. **Study of the catalytic reactivity of silver complexes with hexane and monosubstituted diazoacetates.**

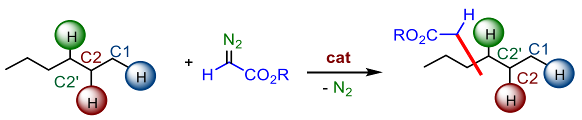

Silver complexes (0.01 mmol) were placed in a Schlenk flask under nitrogen atmosphere with 50 mL of dry hexane. Then the diazocompound (0.15 mmol) was added. For the cases of 2,4-dimethylpentan-3-yl 2-diazoacetate and 2,6-di-tert-butyl-4-methoxyphenyl 2-diazoacetate, the catalyst was dissolved in 30 mL and the diazocompounds were added dissolved in 20 mL. The reactions were protected from light with aluminum foil and allowed to stir at rt for 24 h, before evaluating the result by gas chromatography.

6. **General procedure for reactions with aryl diazoacetates.**

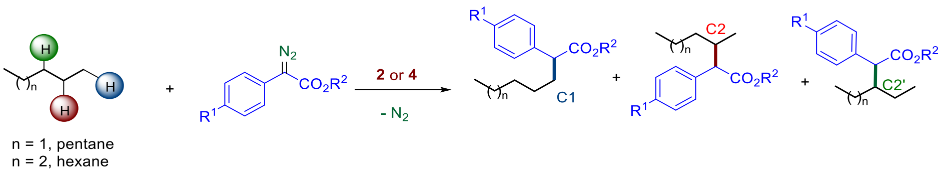

The silver complexes (0.01 mmol) were placed under nitrogen, in an ampoule for catalyst **4** and in a round bottom flask for complex **2**. In both cases, the corresponding alkane (50 mL) was added. Next, the aryl diazocompound (0.15 mmol) dissolved in 30 mL of the alkane was added. The reactions were protected from light with aluminum foil. In the case of the reactions with hexane using catalyst **4**, these were heated at 70 °C for 3 h and at 45 °C for the same time when pentane was used. For complex **2**, the reactions were allowed to stir for 15-30 min at room temperature. Then, the solvent was removed and the crude reaction was studied by ^{1}H NMR with trimethoxybenzene as internal standard. And by GC with calibration curves.

7. **Study of the catalytic reactivity of silver complexes with hexane and diethyl diazomalonate.**

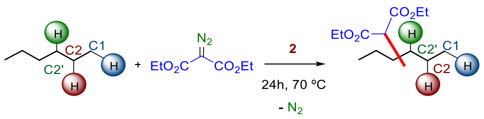

The silver complexes (0.01 mmol) were placed under nitrogen in an ampoule. Hexane (50 mL) and the diazo reagent (0.15 mmol) were added and the reactions were heated at 70 °C for 24 h. The solvent was then removed under vaccum and the crude reaction was studied by ^{1}H NMR with trimethoxybenzene as internal standard.

8. **NMR spectra and GC traces of reaction crudes.**

__Reactions employing monosubstituted diazoacetates__.

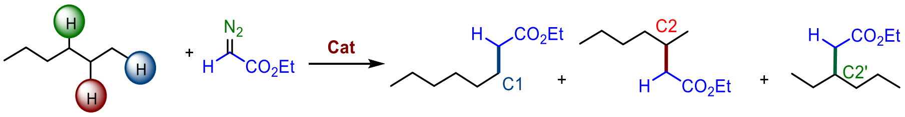

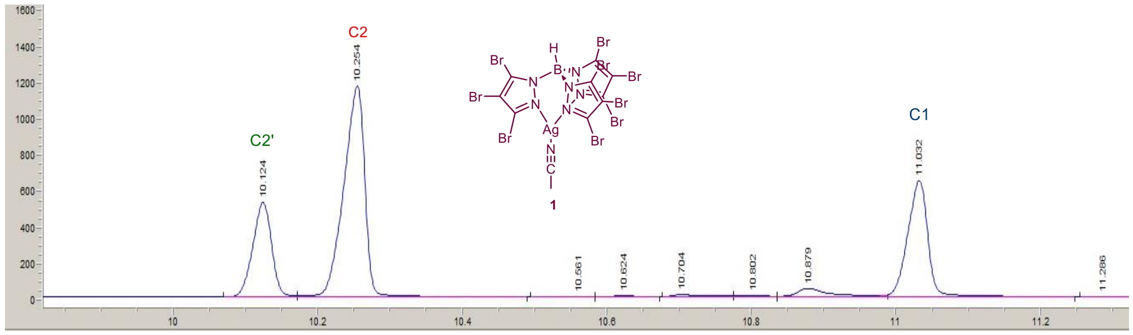

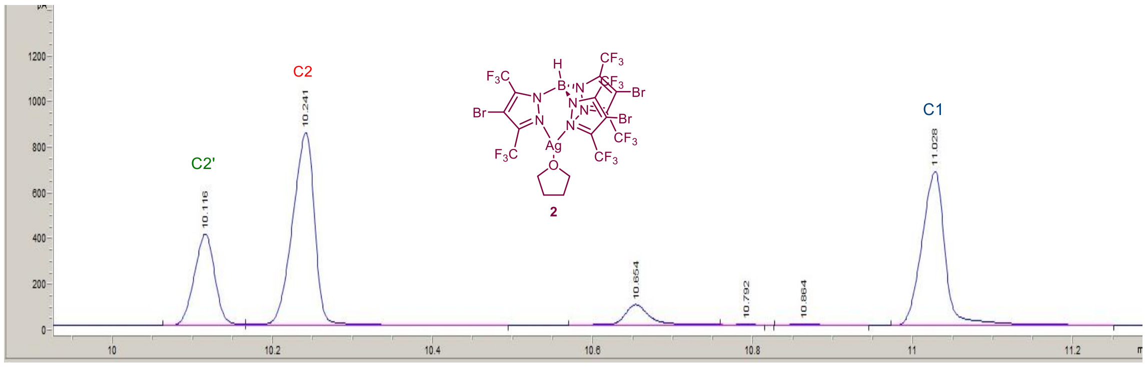

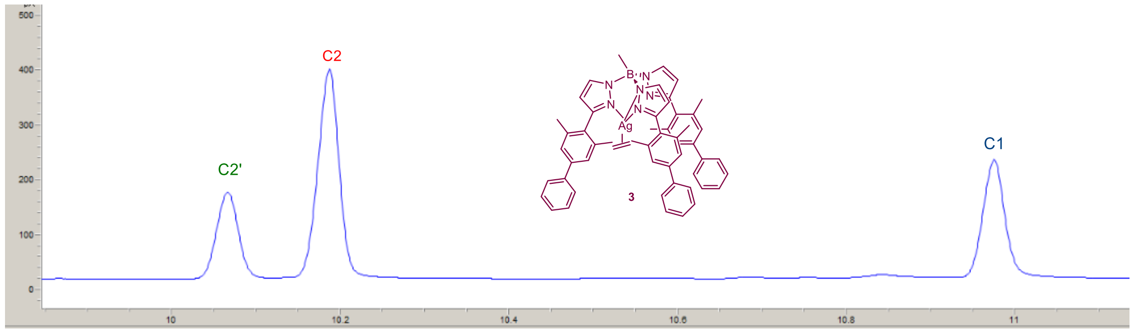

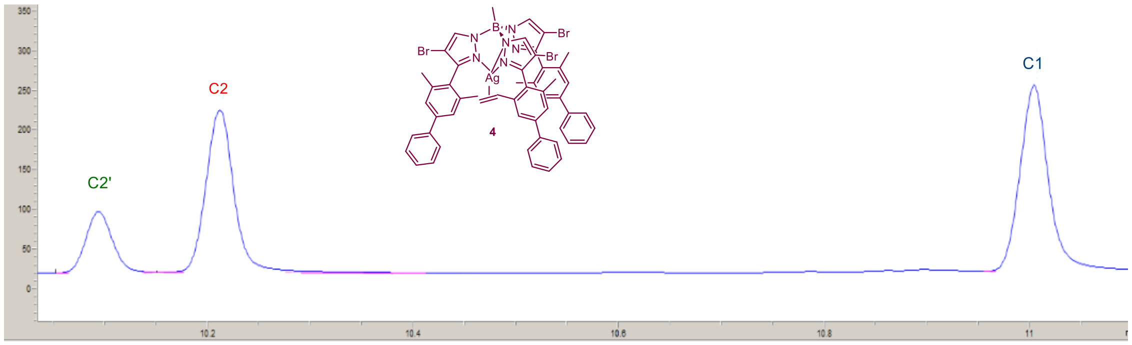

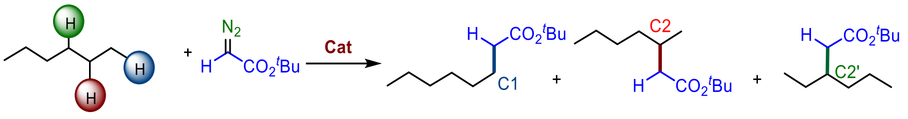
Cat
C1
C2
C2'

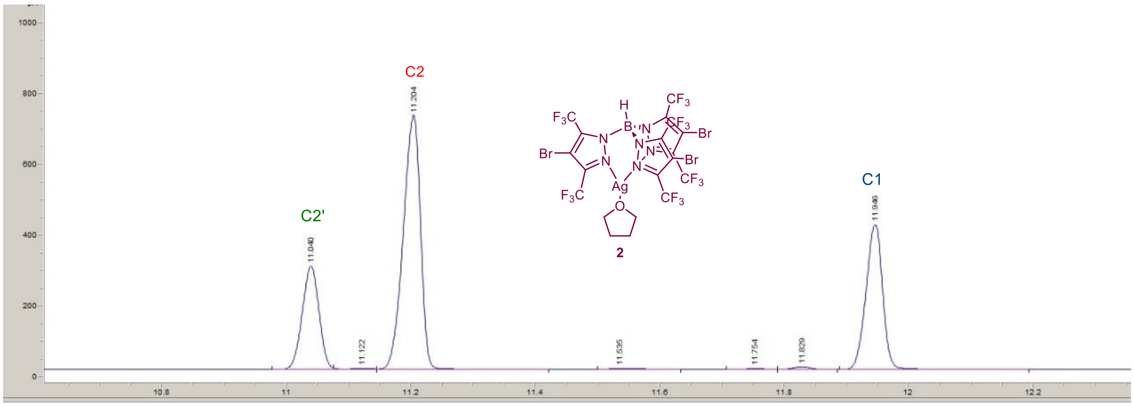
C2'
C2
C1
11.040
11.122
11.204
11.535
11.754
11.829
11.946
2

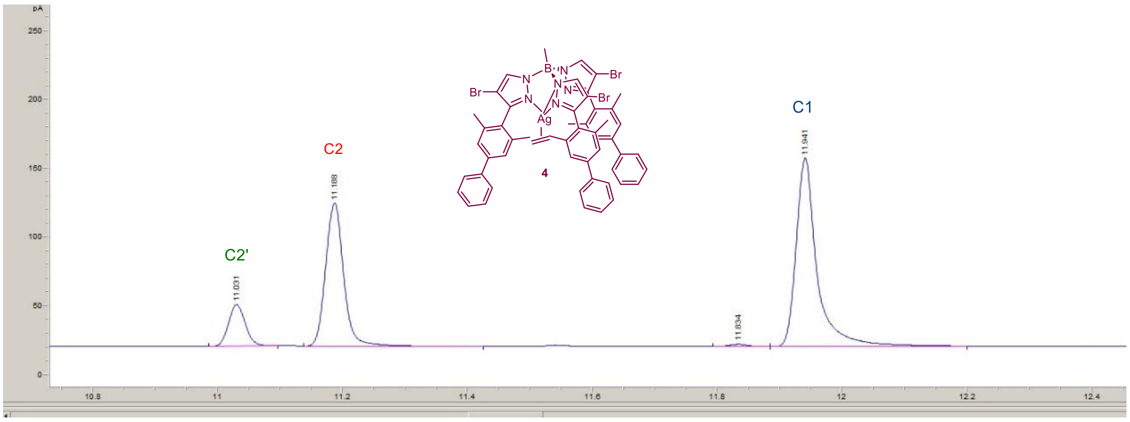
C2'
C2
C1
11.031
11.188
11.834
11.941
4

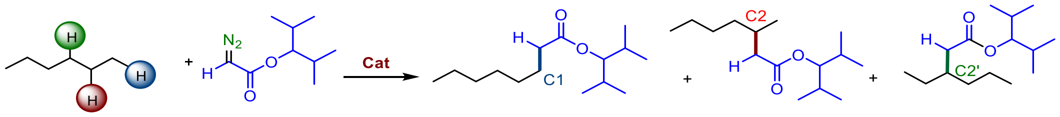
Cat
C1
C2
C2'

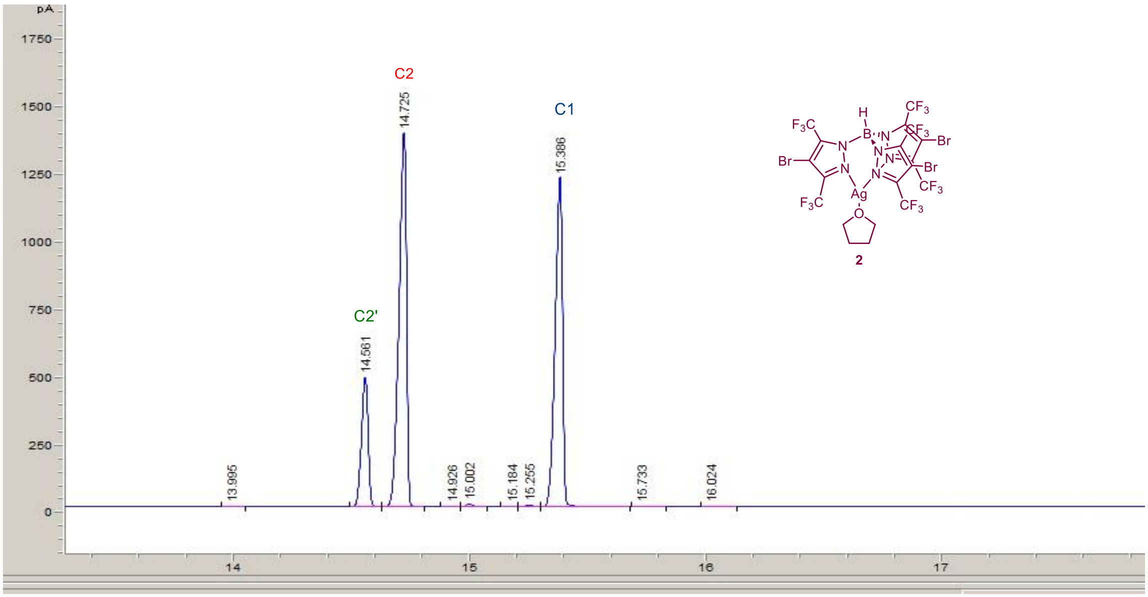
C2'
C2
C1
13.995
14.561
14.725
14.926
15.002
15.184
15.255
15.386
15.733
16.024
2

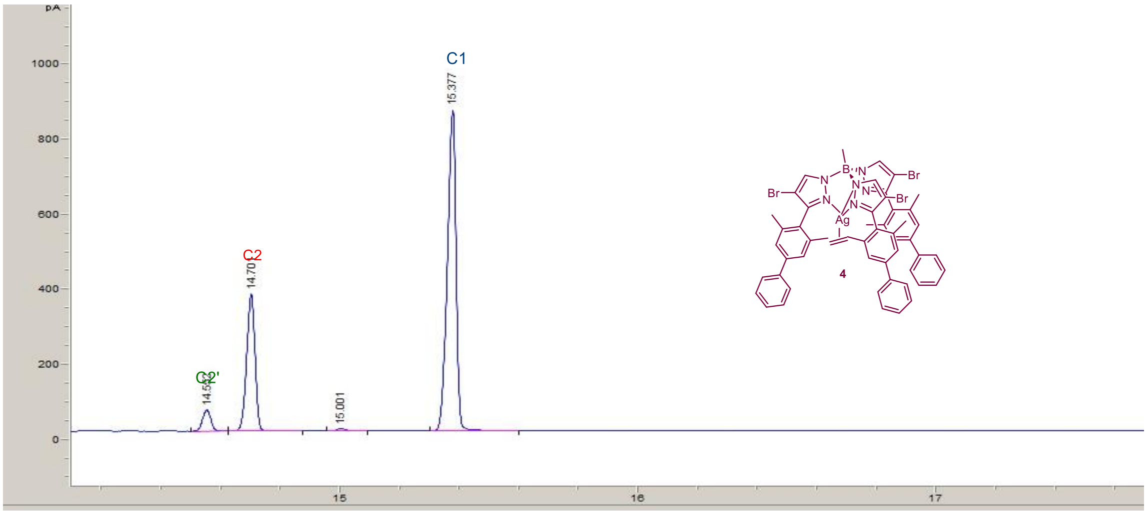
pA
C1
15.377
C2
14.707
C2'
14.55
15.001
4
Br
B
N
Ag
15
16
17

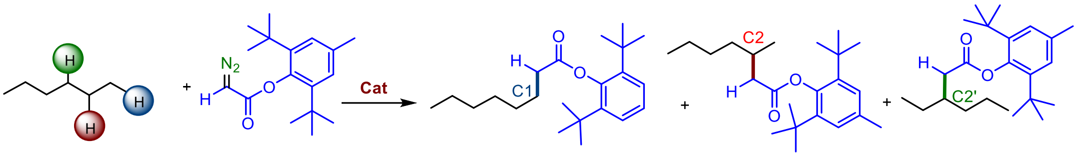
H
H
H
N2
Cat
C1
C2
C2'
+

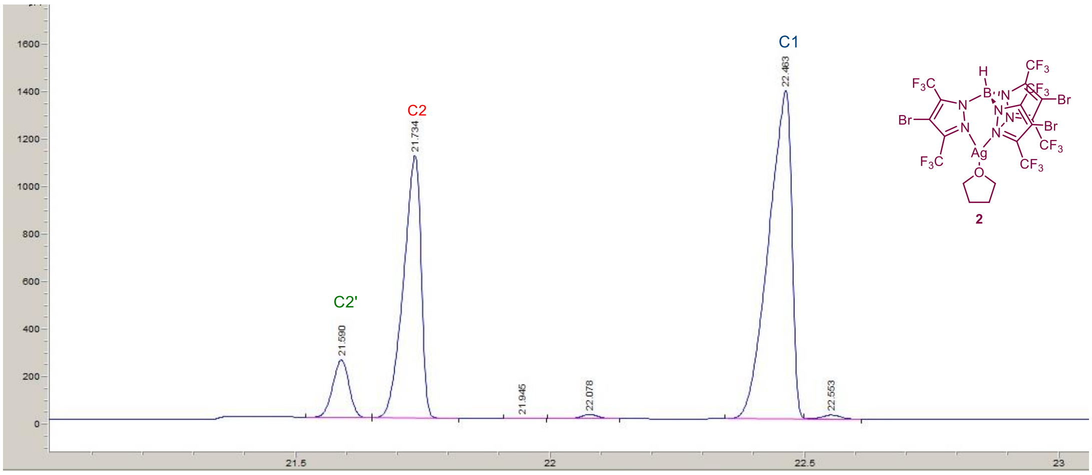
C1
22.463
C2
21.734
C2'
21.590
21.945
22.078
22.553
2
21.5
22
22.5
23

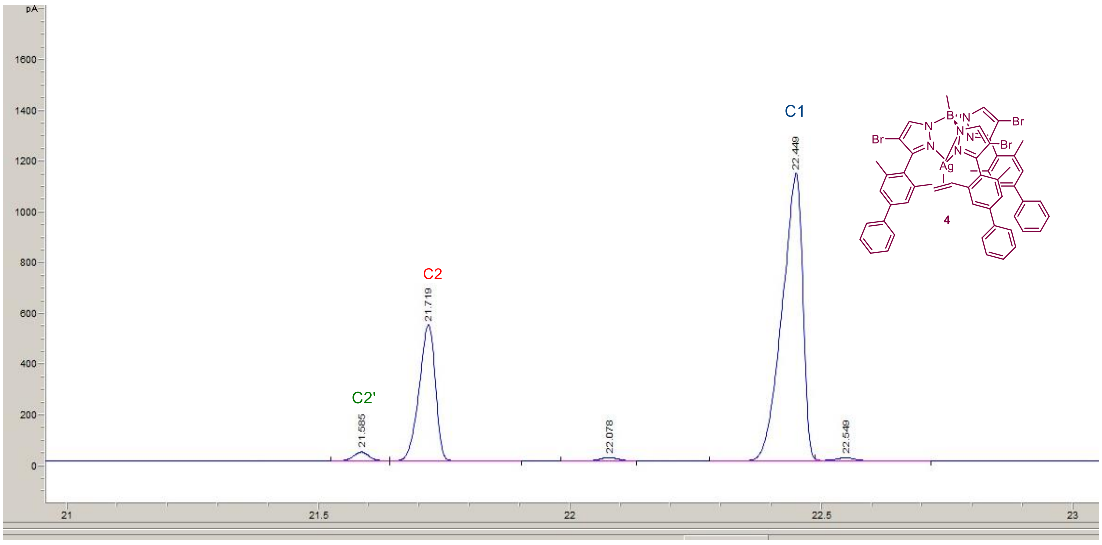
pA
C1
22.449
C2
21.719
C2'
21.585
22.078
22.549
4
21
21.5
22
22.5
23

Reactions employing aryl diazoacetates.

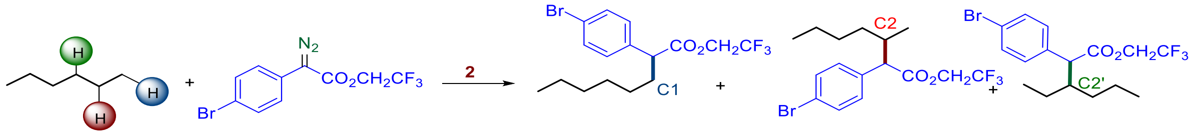

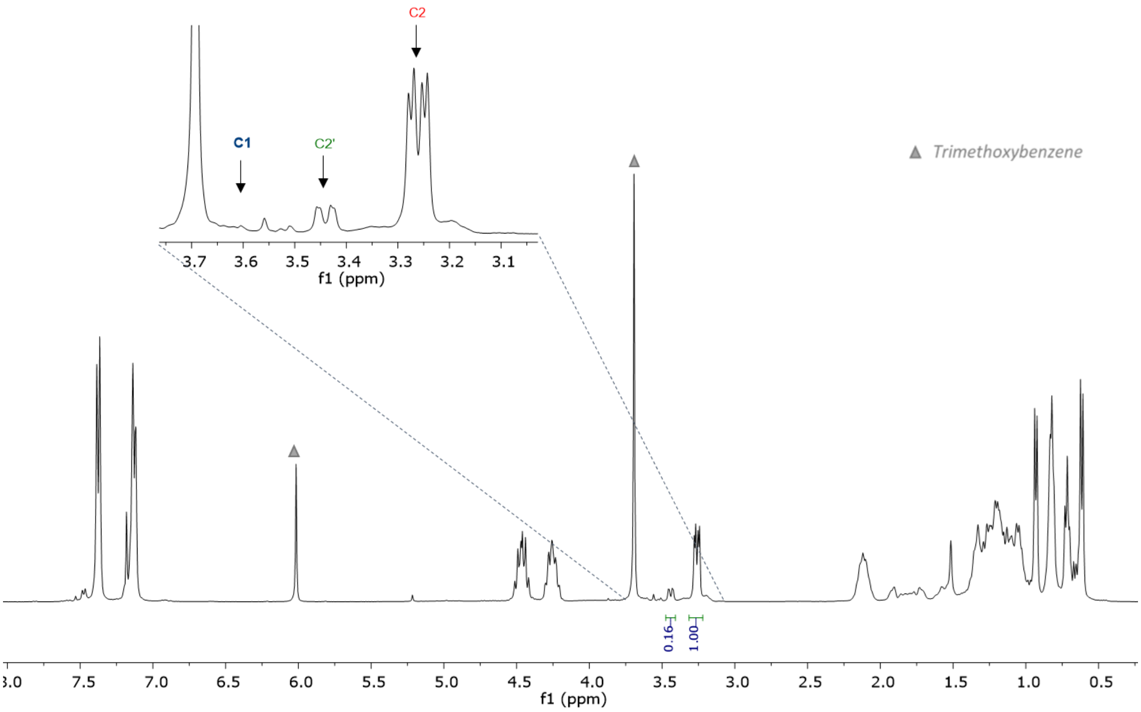

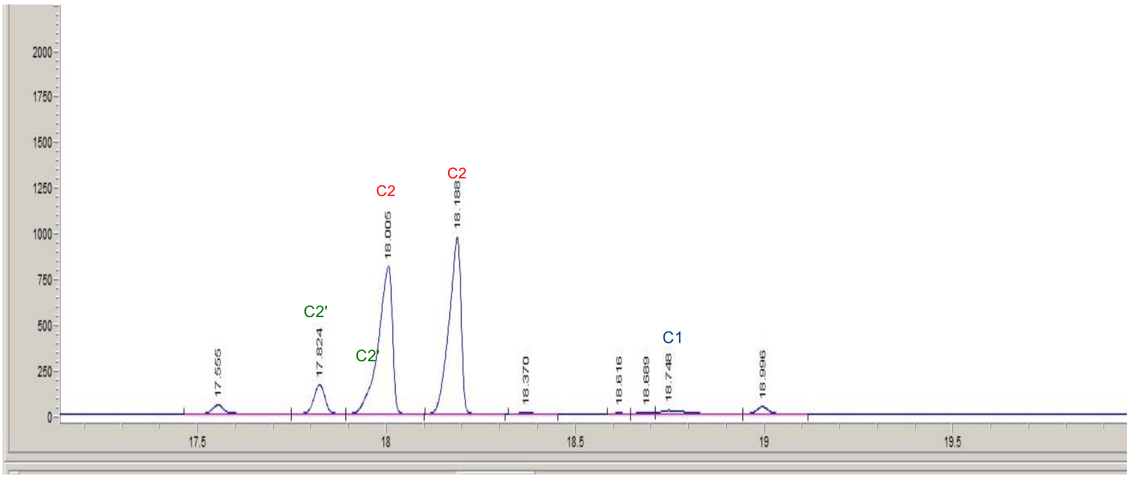

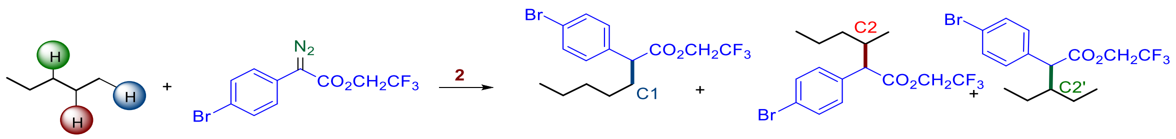
H
H
H
+
Br
N_2
$CO_2CH_2CF_3$
2
Br
$CO_2CH_2CF_3$
C1
+
C2
$CO_2CH_2CF_3$
Br
+
Br
$CO_2CH_2CF_3$
C2'

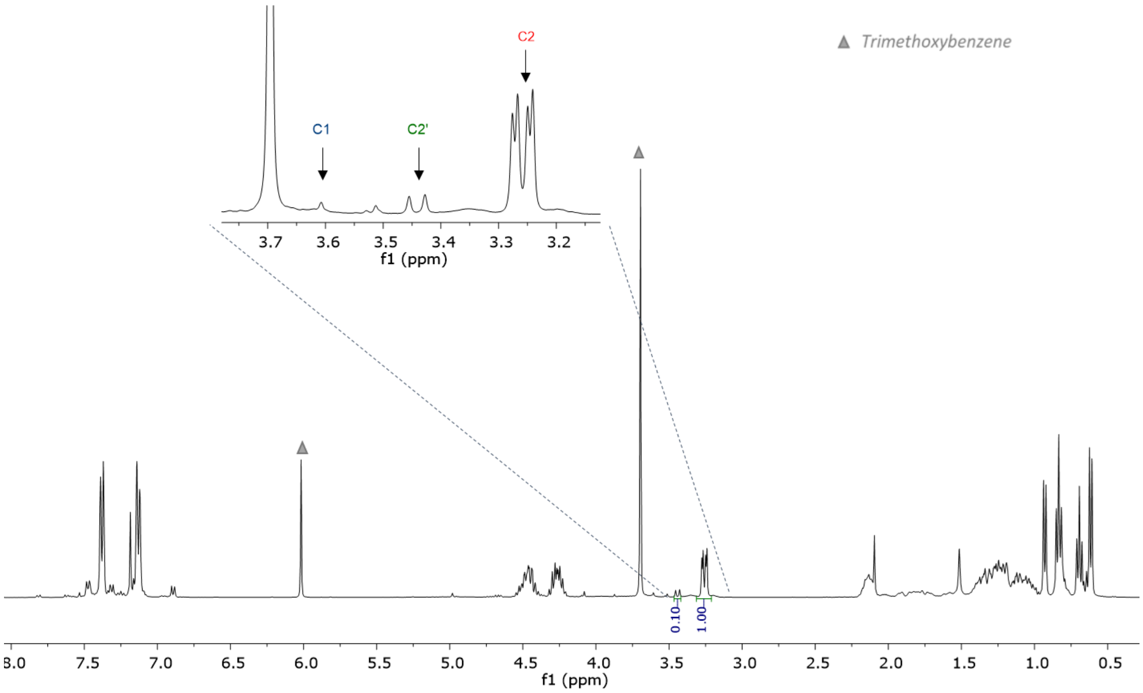
Trimethoxybenzene
C2
C1
C2'
3.7
3.6
3.5
3.4
3.3
3.2
f1 (ppm)
0.10
1.00
8.0
7.5
7.0
6.5
6.0
5.5
5.0
4.5
4.0
3.5
3.0
2.5
2.0
1.5
1.0
0.5
f1 (ppm)

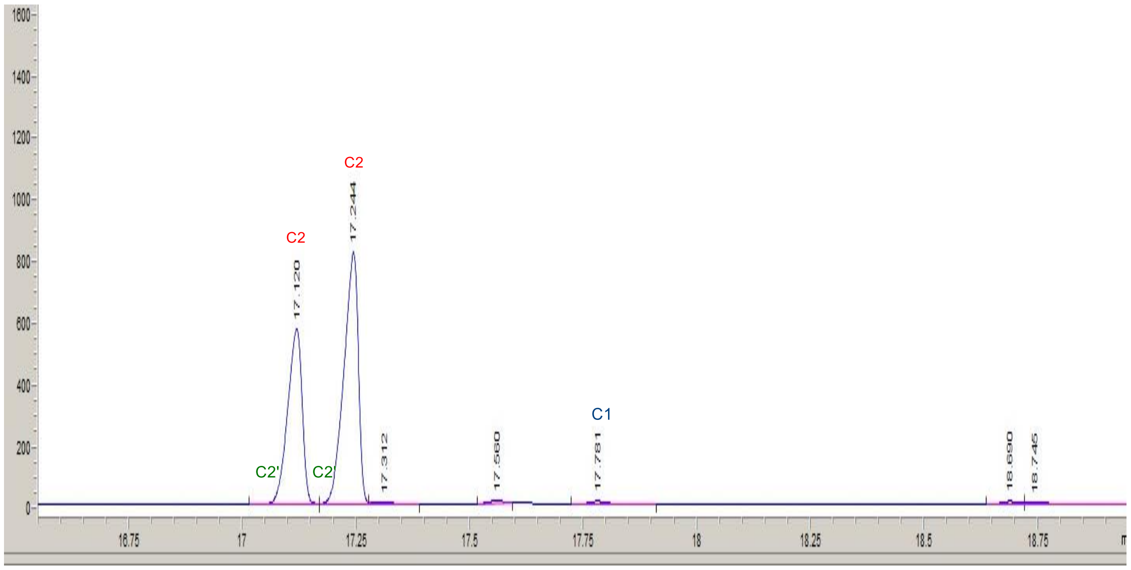
1600
1400
1200
1000
800
600
400
200
0
C2
C2
17.120
17.244
C2'
C2'
17.312
17.560
C1
17.781
18.690
18.745
16.75
17
17.25
17.5
17.75
18
18.25
18.5
18.75

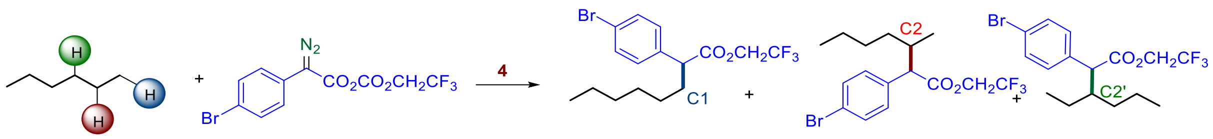
N2
CO2CO2CH2CF3
Br
4
Br
CO2CH2CF3
C1
C2
CO2CH2CF3
Br
Br
CO2CH2CF3
C2'

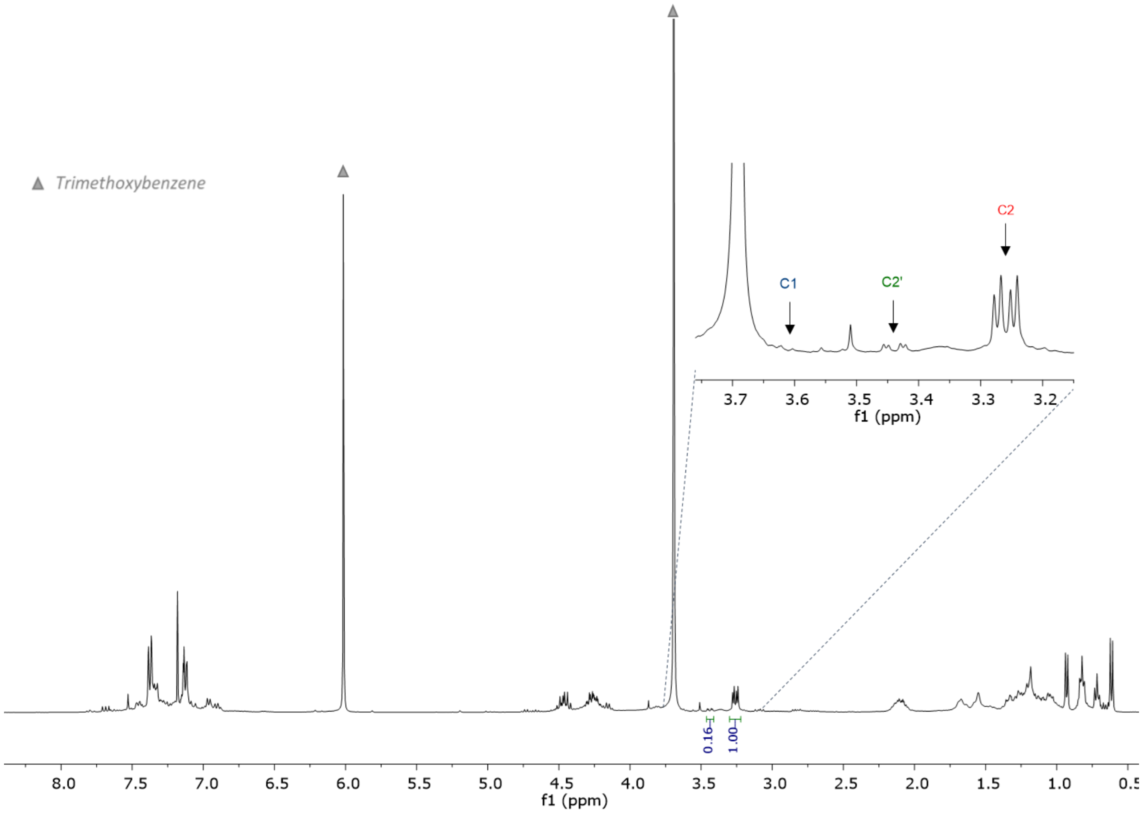
Trimethoxybenzene
C1
C2'
C2
f1 (ppm)
0.16
1.00

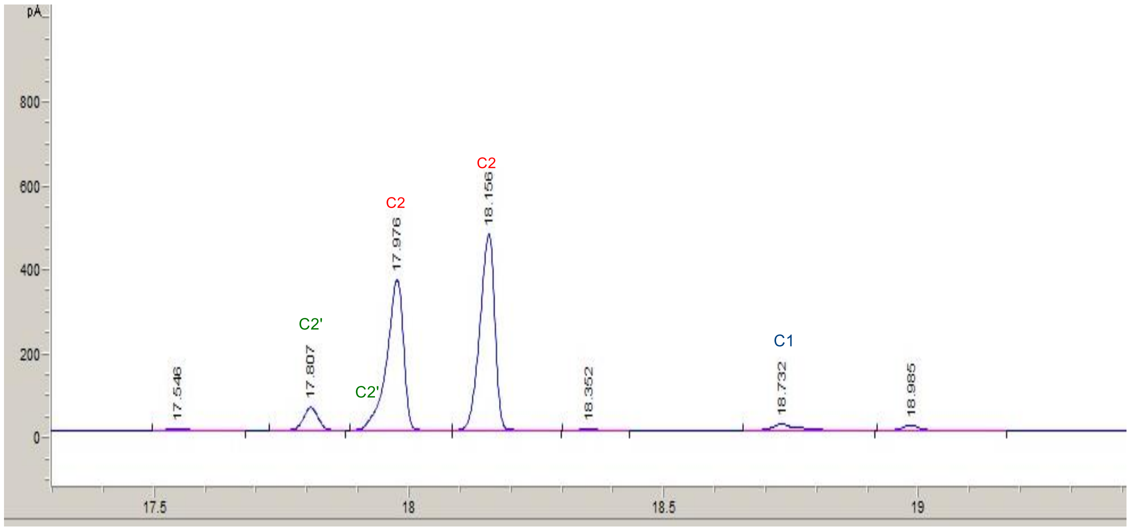
pA
C2'
C2
C2
C1
17.546
17.807
17.976
18.156
18.352
18.732
18.985

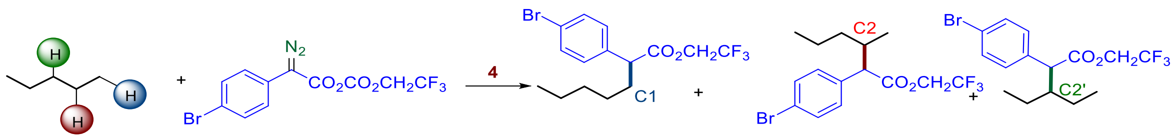
H
H
H
N2
CO2CO2CH2CF3
Br
4
Br
CO2CH2CF3
C1
C2
CO2CH2CF3
Br
Br
CO2CH2CF3
C2'

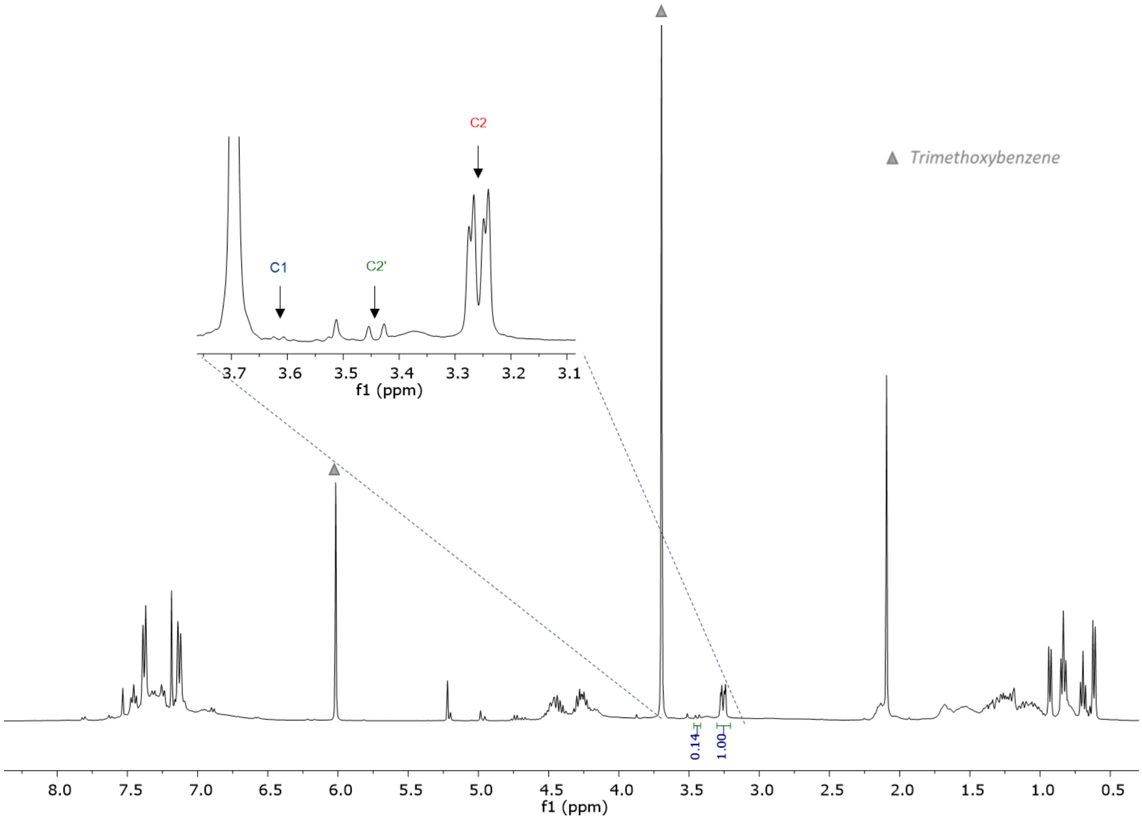
C2
C1
C2'
3.7
3.6
3.5
3.4
3.3
3.2
3.1
f1 (ppm)
Trimethoxybenzene
0.14
1.00
8.0
7.5
7.0
6.5
6.0
5.5
5.0
4.5
4.0
3.5
3.0
2.5
2.0
1.5
1.0
0.5
f1 (ppm)

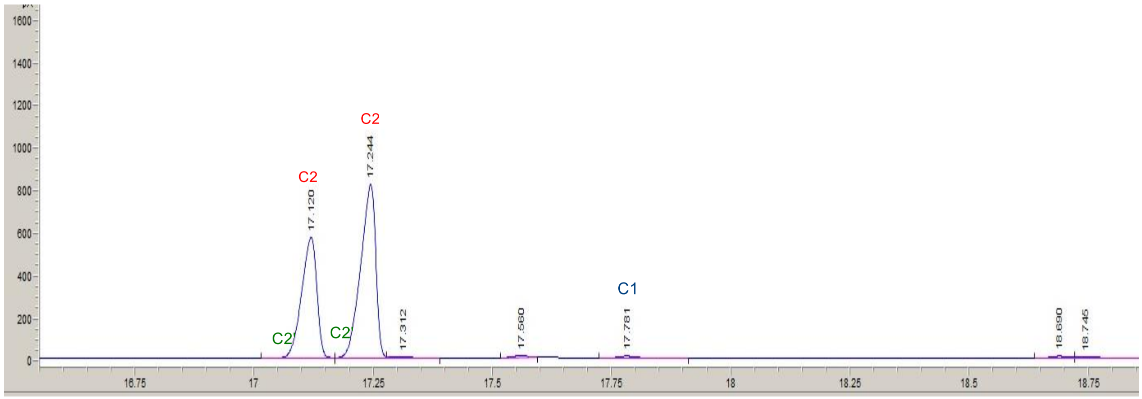
C2
C2
C2'
C2
C1
17.120
17.244
17.312
17.560
17.781
18.690
18.745
16.75
17
17.25
17.5
17.75
18
18.25
18.5
18.75

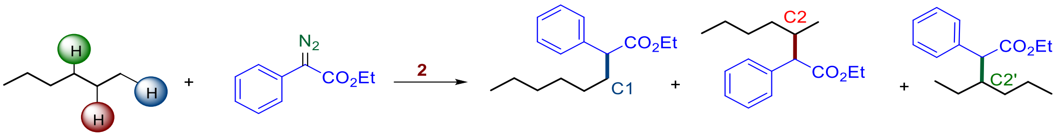
H
H
H
N2
CO2Et
2
CO2Et
C1
C2
CO2Et
CO2Et
C2'
+

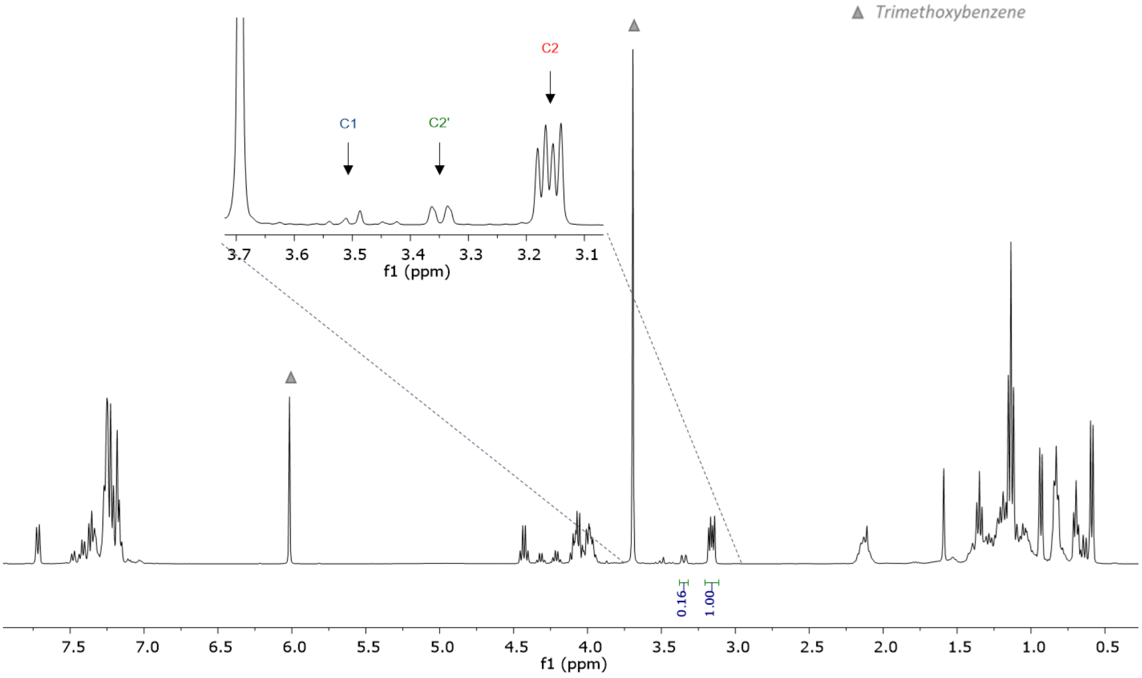
Trimethoxybenzene
C1
C2'
C2
3.7
3.6
3.5
3.4
3.3
3.2
3.1
f1 (ppm)
0.16
1.00
7.5
7.0
6.5
6.0
5.5
5.0
4.5
4.0
3.5
3.0
2.5
2.0
1.5
1.0
0.5
f1 (ppm)

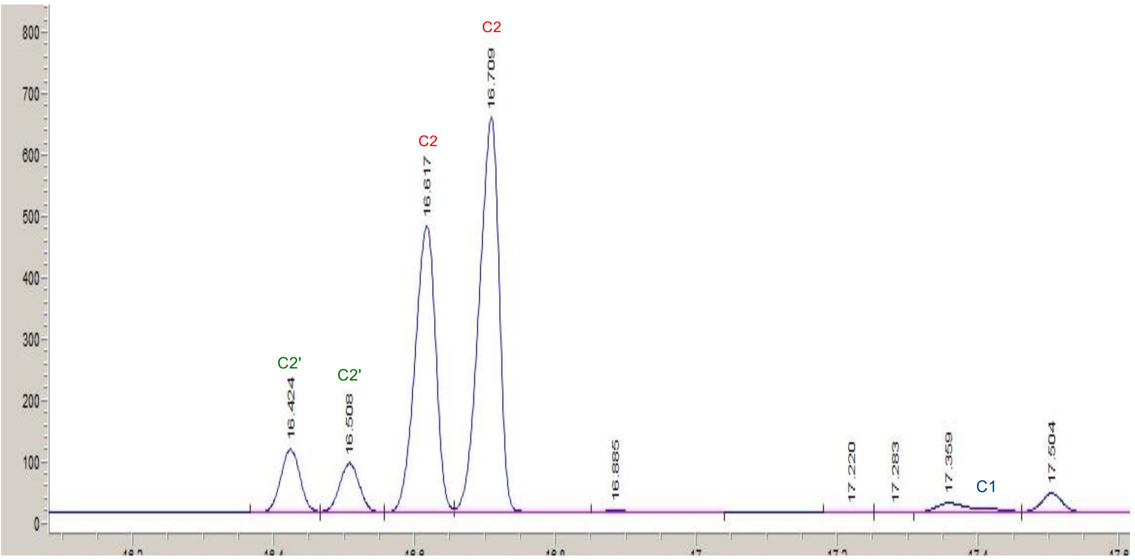
800
700
600
500
400
300
200
100
0
C2'
16.424
C2'
16.508
C2
16.617
C2
16.709
16.885
17.220
17.283
17.359
C1
17.504

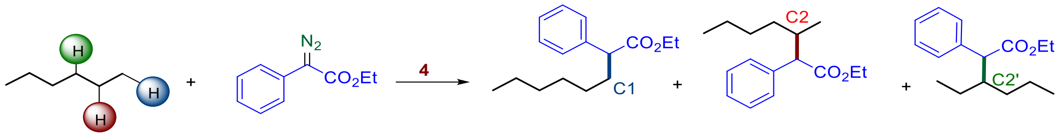

H
H
H
+
N2
CO2Et
4
CO2Et
C1
+
C2
CO2Et
+
CO2Et
C2'

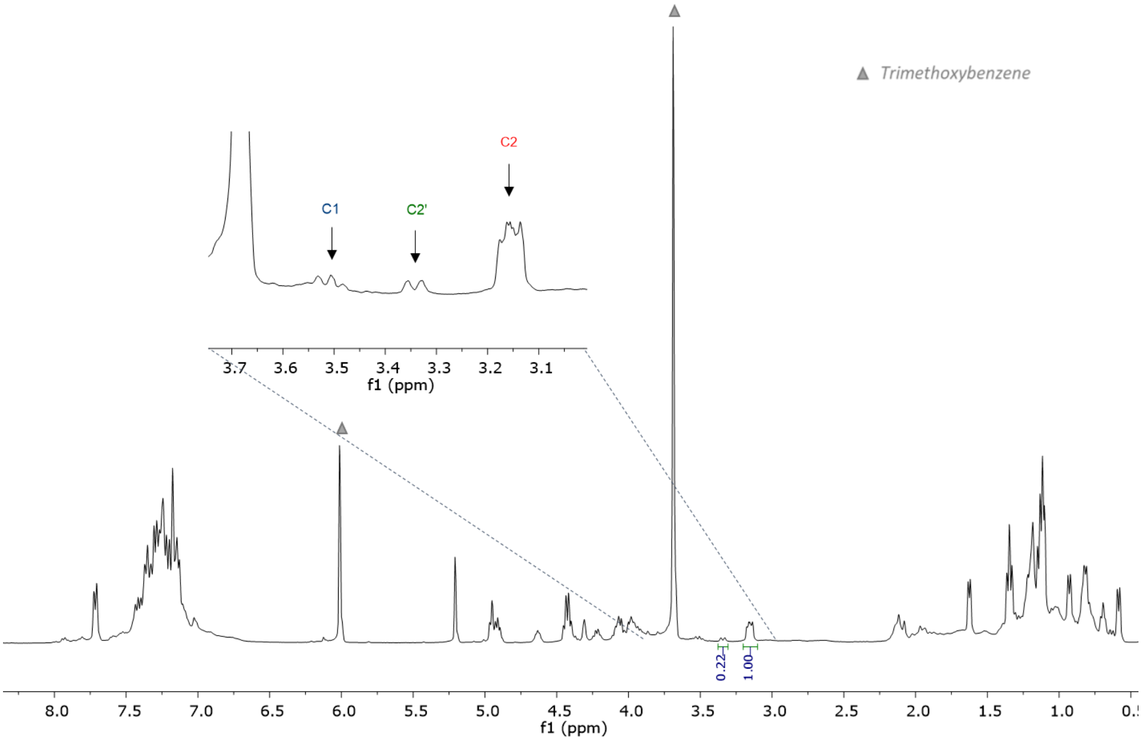

Trimethoxybenzene
C2
C1
C2'
3.7
3.6
3.5
3.4
3.3
3.2
3.1
f1 (ppm)
0.22
1.00
8.0
7.5
7.0
6.5
6.0
5.5
5.0
4.5
4.0
3.5
3.0
2.5
2.0
1.5
1.0
f1 (ppm)

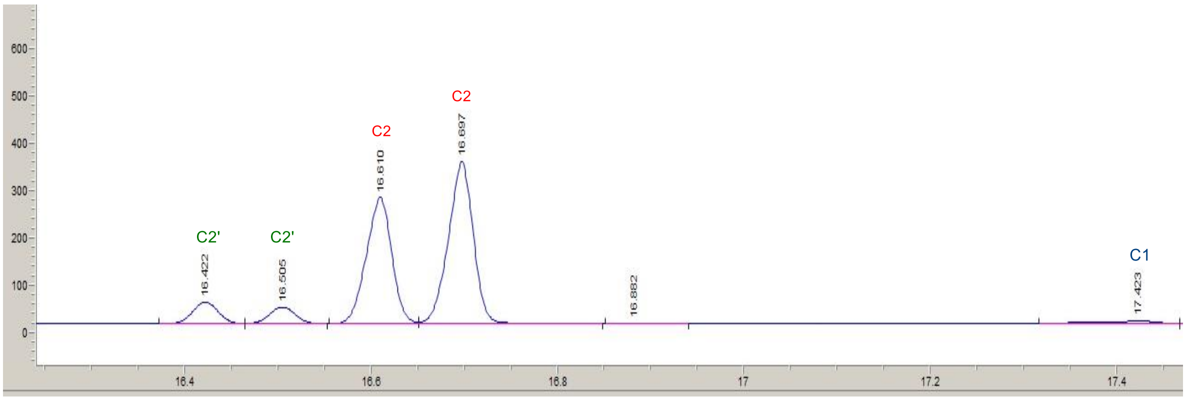

600
500
400
300
200
100
0
C2'
16.422
C2'
16.505
C2
16.610
C2
16.697
16.882
C1
17.423
16.4
16.6
16.8
17
17.2
17.4

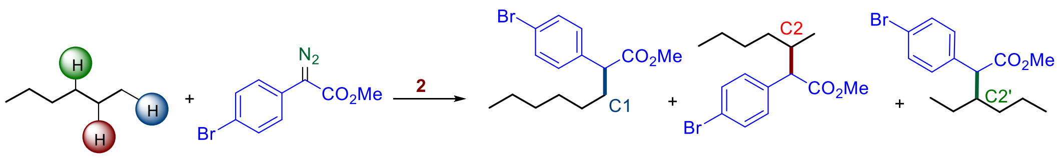

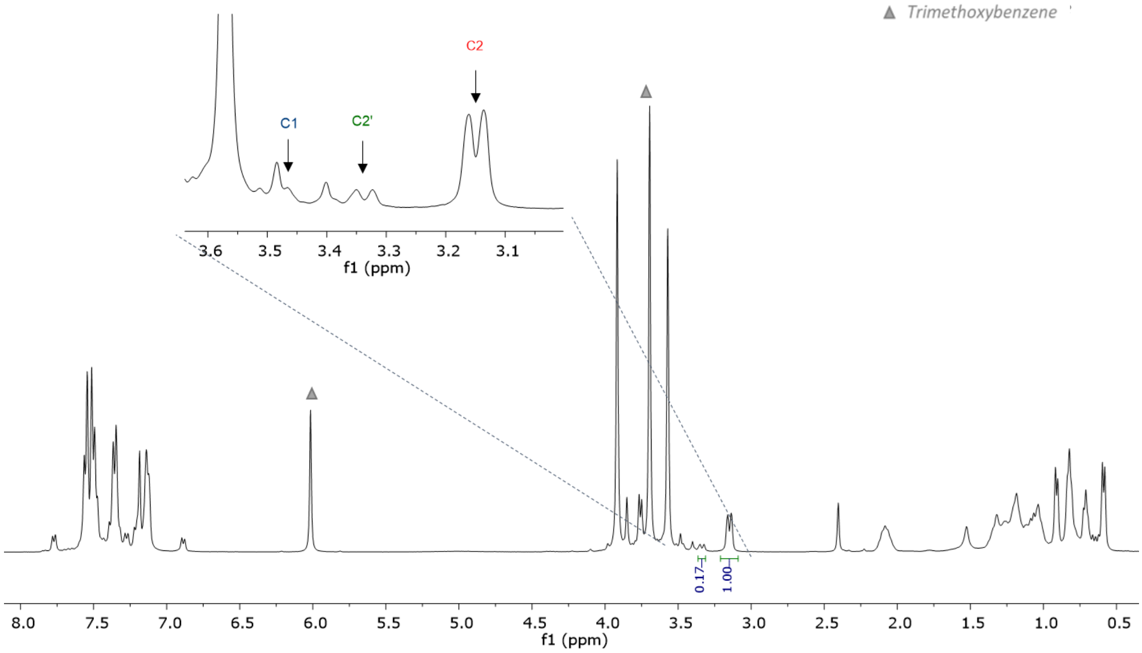

Trimethoxybenzene
C1
C2'
C2
f1 (ppm)
0.17
1.00

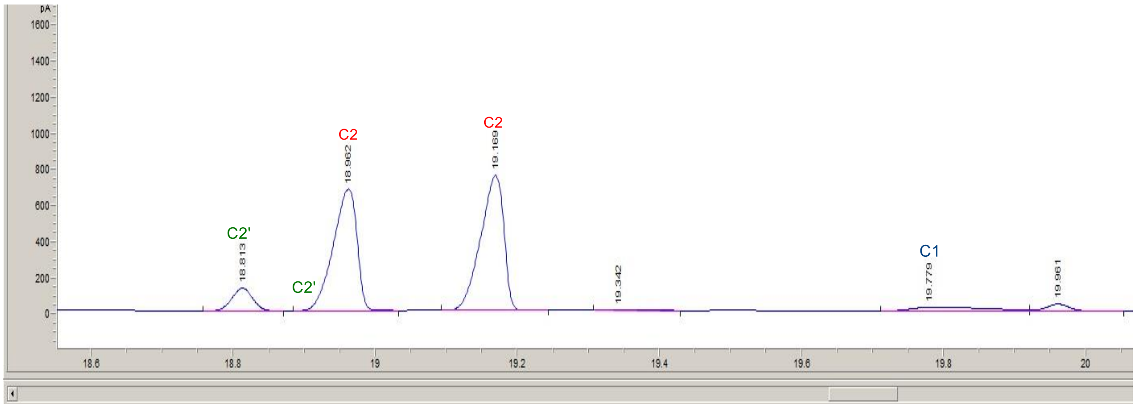

C2'
C2
C2
C1
C2'

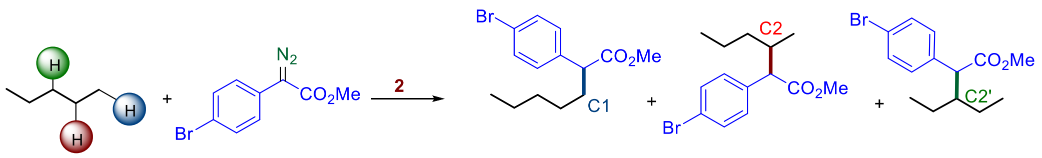
H
H
H
N2
CO2Me
Br
2
Br
CO2Me
C1
C2
CO2Me
Br
Br
CO2Me
C2'

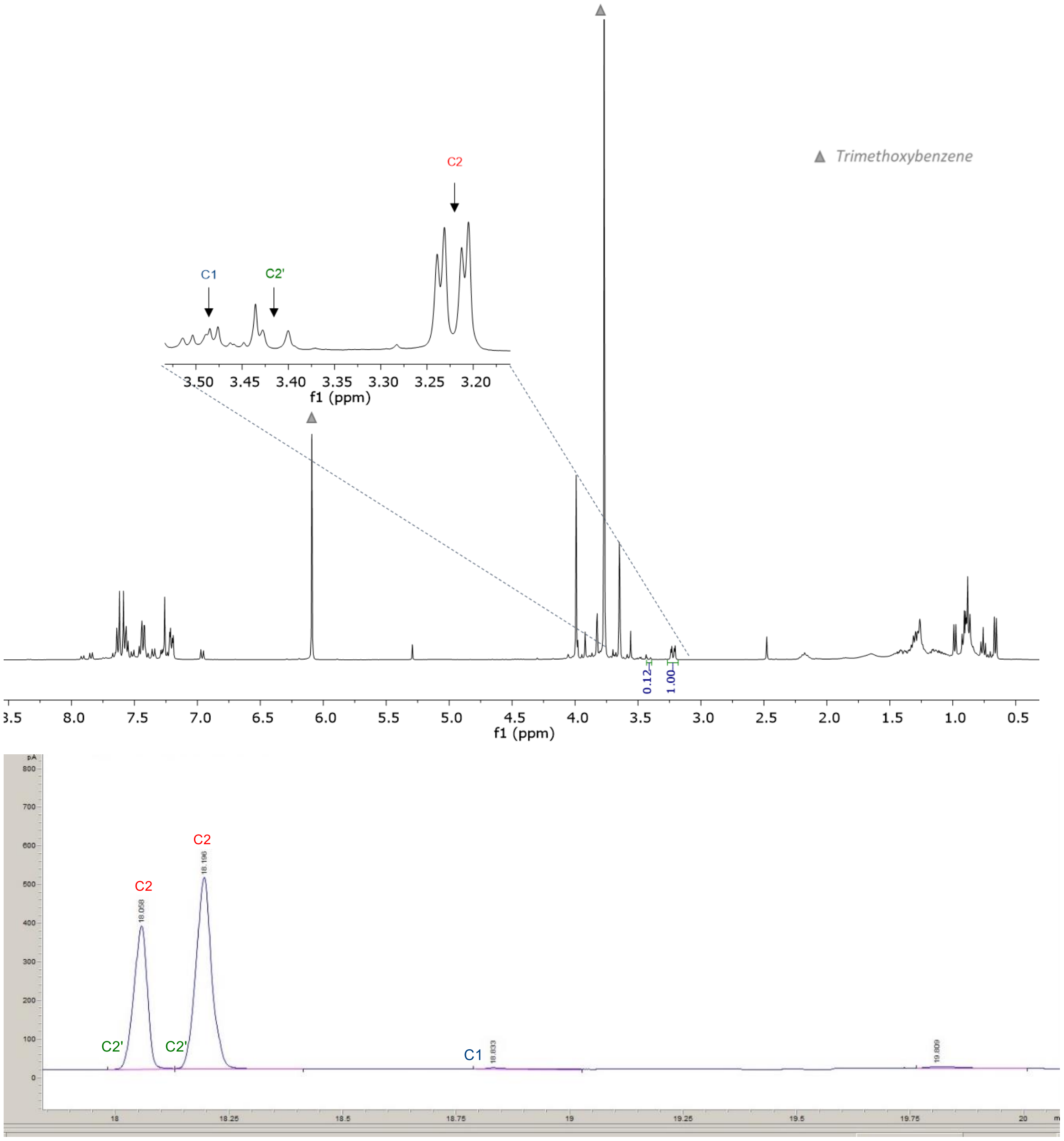
Trimethoxybenzene
C1
C2'
C2
3.50 3.45 3.40 3.35 3.30 3.25 3.20
f1 (ppm)
0.12
1.00
8.0 7.5 7.0 6.5 6.0 5.5 5.0 4.5 4.0 3.5 3.0 2.5 2.0 1.5 1.0 0.5
f1 (ppm)
C2
C2
C2'
C2'
C1
18.058
18.196
18.833
19.809

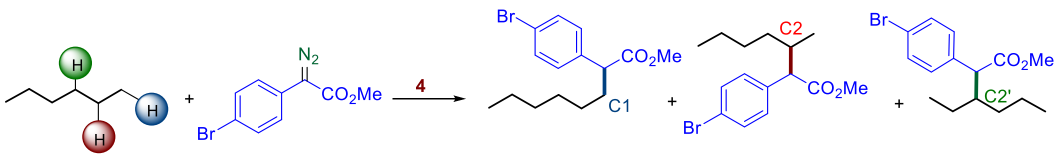
H
H
H
+
N2
CO2Me
Br
4
Br
CO2Me
C1
+
C2
CO2Me
Br
+
Br
CO2Me
C2'

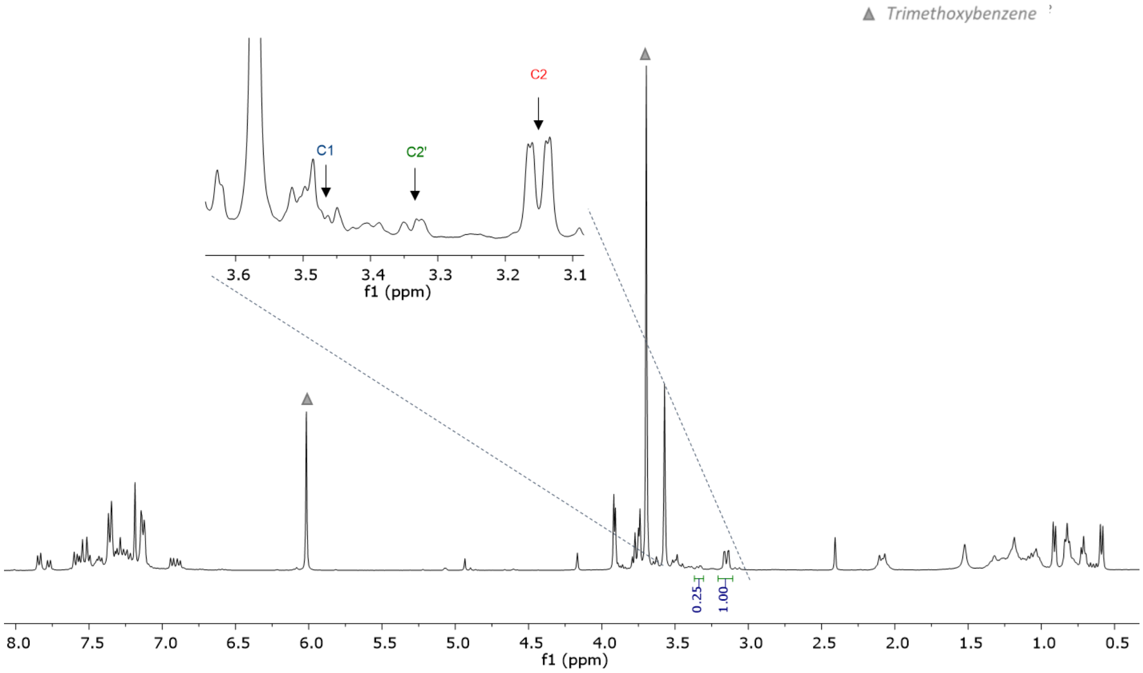
Trimethoxybenzene
C2
C1
C2'
3.6
3.5
3.4
3.3
3.2
3.1
f1 (ppm)
0.25
1.00
8.0
7.5
7.0
6.5
6.0
5.5
5.0
4.5
4.0
3.5
3.0
2.5
2.0
1.5
1.0
0.5
f1 (ppm)

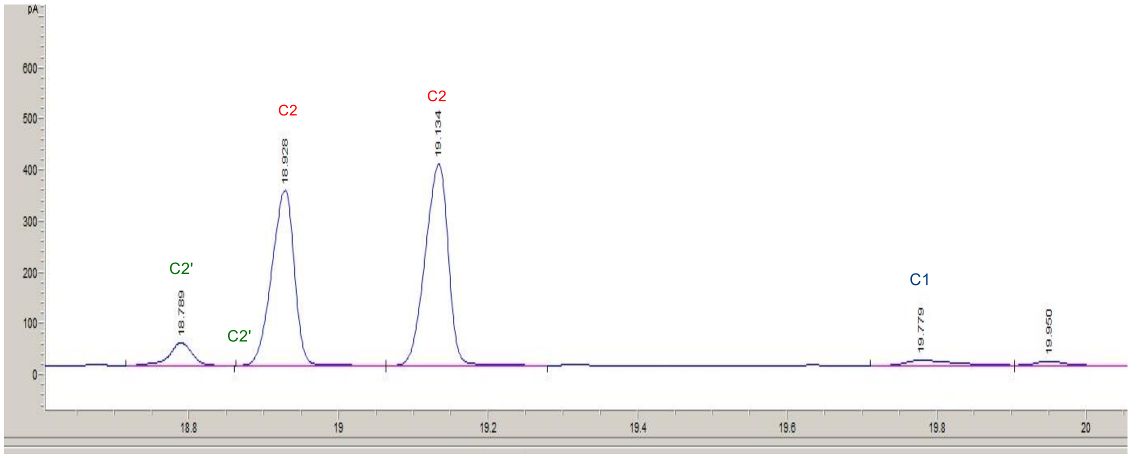
pA
600
500
400
300
200
100
0
C2'
18.789
C2'
C2
18.928
C2
19.134
C1
19.779
19.950
18.8
19
19.2
19.4
19.6
19.8
20

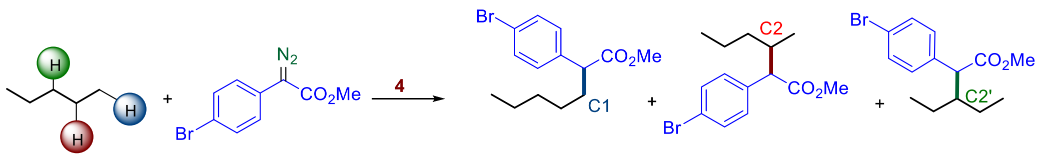
H
H
H
N2
CO2Me
Br
4
Br
CO2Me
C1
C2
CO2Me
Br
Br
CO2Me
C2'

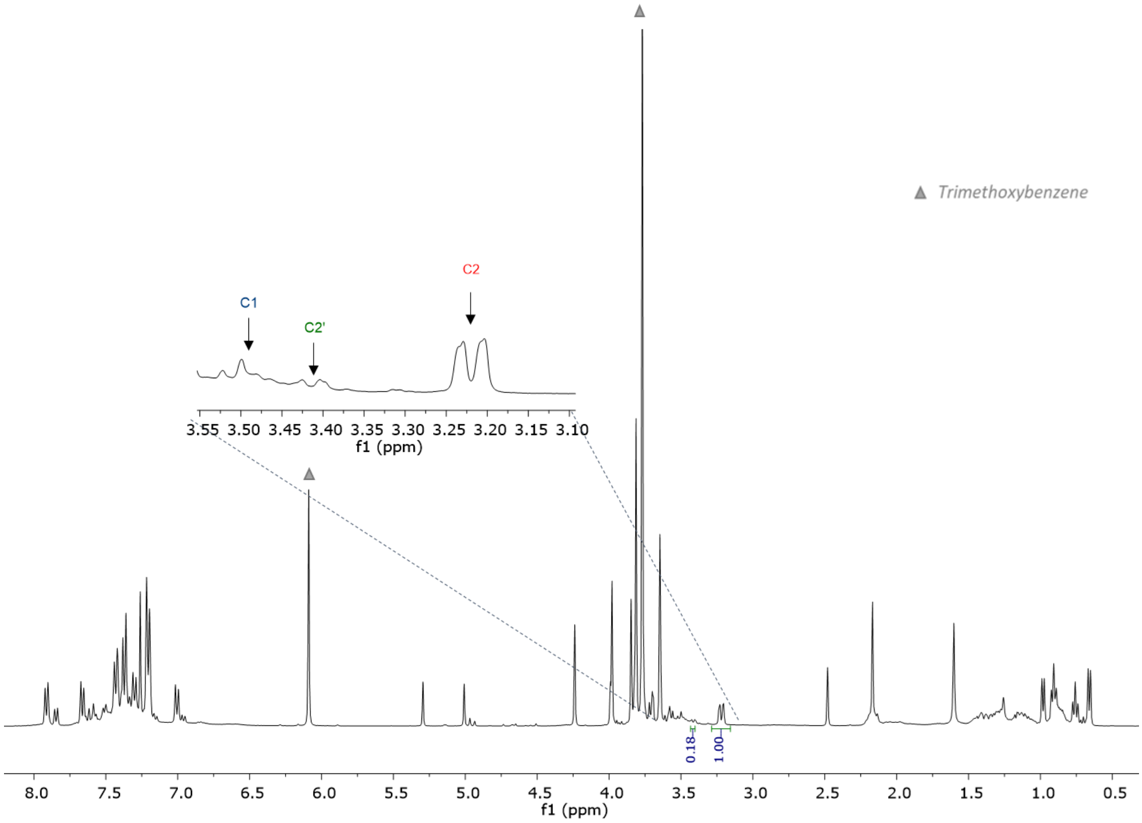
Trimethoxybenzene
C1
C2'
C2
3.55 3.50 3.45 3.40 3.35 3.30 3.25 3.20 3.15 3.10
f1 (ppm)
0.18
1.00
8.0 7.5 7.0 6.5 6.0 5.5 5.0 4.5 4.0 3.5 3.0 2.5 2.0 1.5 1.0 0.5
f1 (ppm)

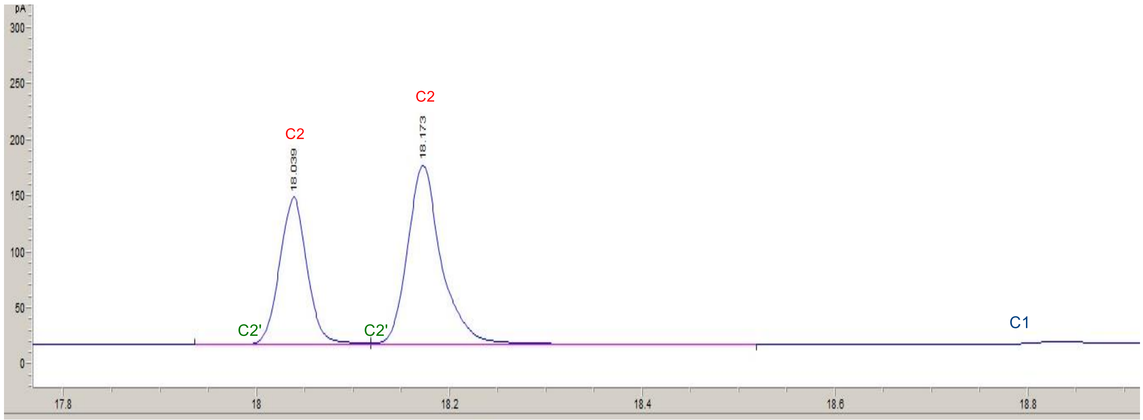
pA
C2
18.039
C2
18.173
C2'
C2'
C1
17.8
18
18.2
18.4
18.6
18.8

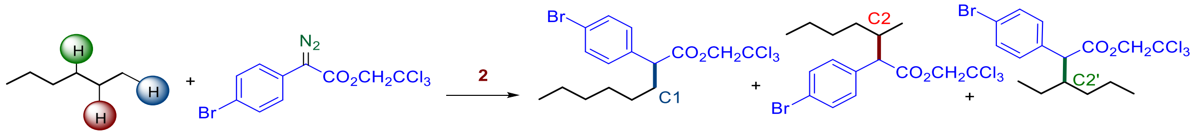

H
H
H
N2
CO2CH2CCl3
Br
2
Br
CO2CH2CCl3
C1
C2
CO2CH2CCl3
Br
Br
CO2CH2CCl3
C2'

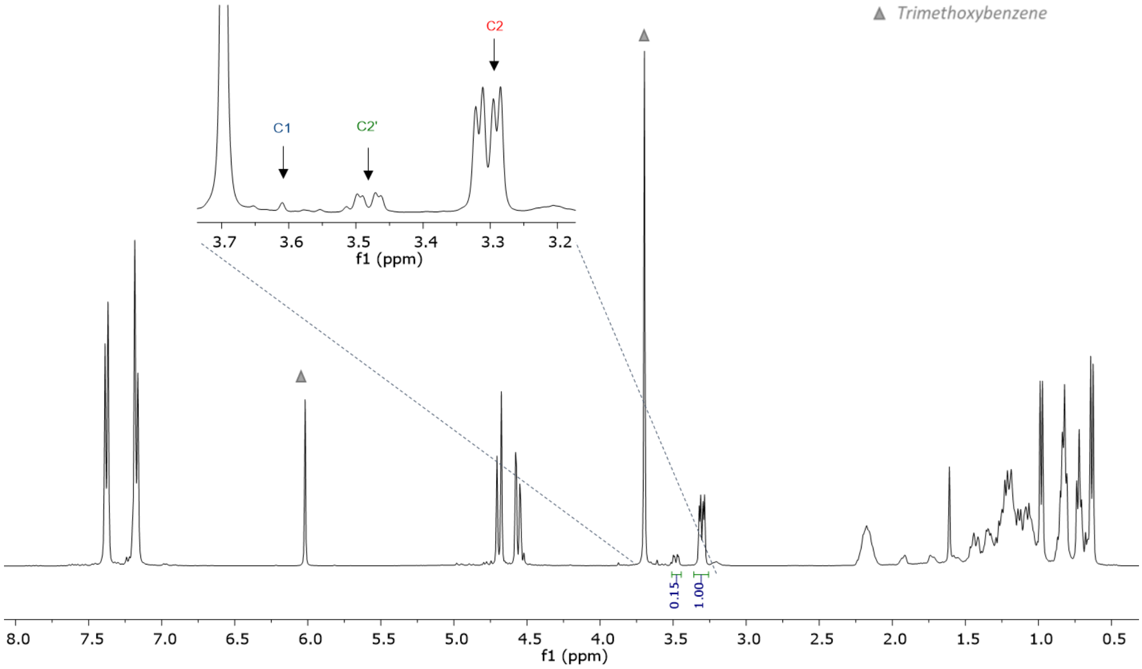

Trimethoxybenzene
C2
C1
C2'
3.7
3.6
3.5
3.4
3.3
3.2
f1 (ppm)
0.15
1.00
8.0
7.5
7.0
6.5
6.0
5.5
5.0
4.5
4.0
3.5
3.0
2.5
2.0
1.5
1.0
0.5
f1 (ppm)

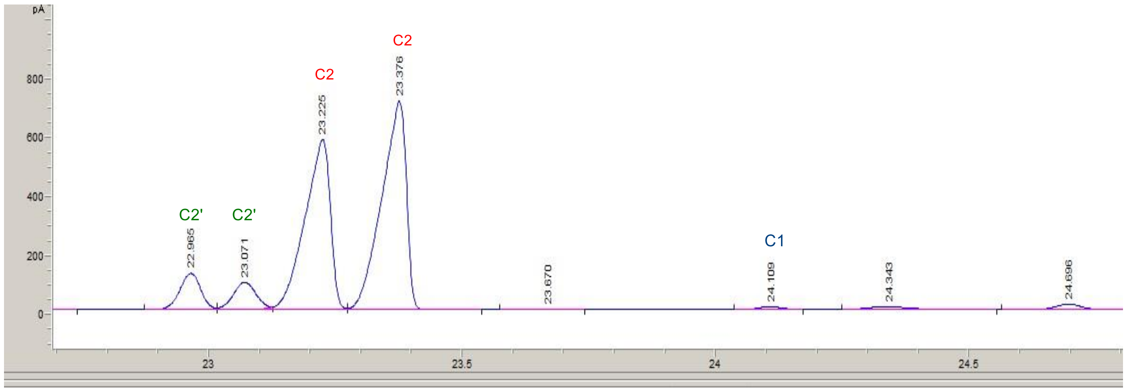

pA
800
600
400
200
0
C2'
22.965
C2'
23.071
C2
23.225
C2
23.376
23.670
C1
24.109
24.343
24.696
23
23.5
24
24.5

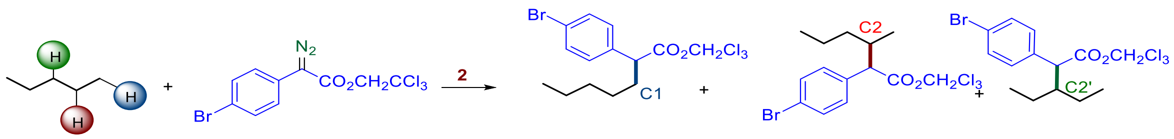

H
H
H
+
N2
Br
CO2CH2CCl3
2
Br
CO2CH2Cl3
C1
+
C2
CO2CH2Cl3
Br
+
Br
CO2CH2Cl3
C2'

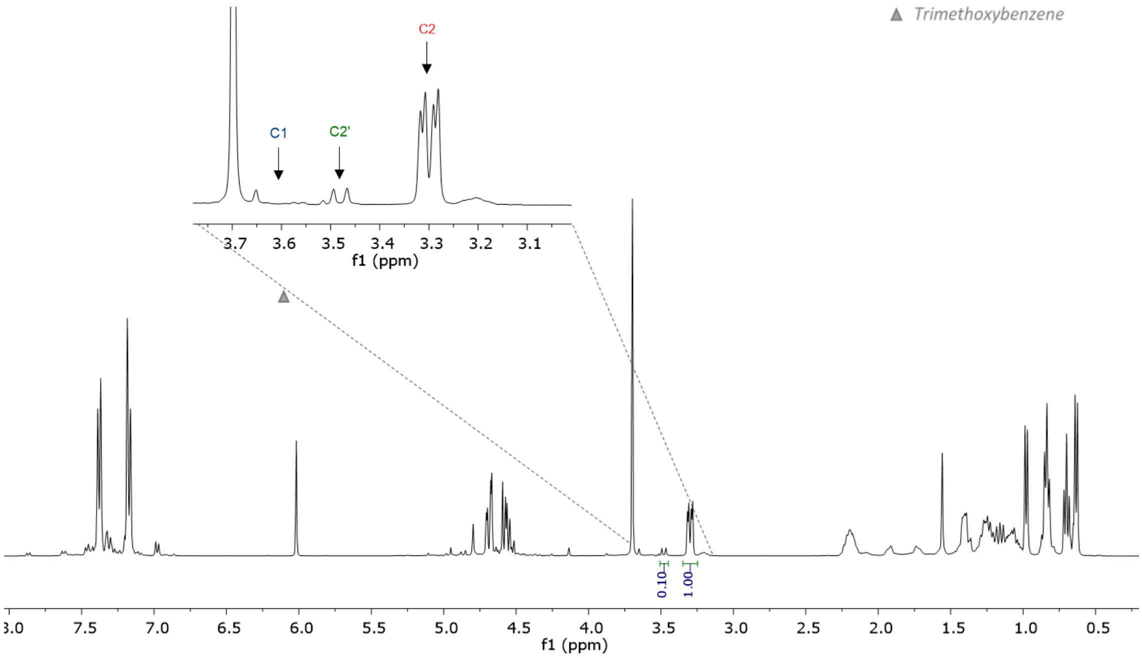

Trimethoxybenzene
C2
C1
C2'
f1 (ppm)
0.10
1.00

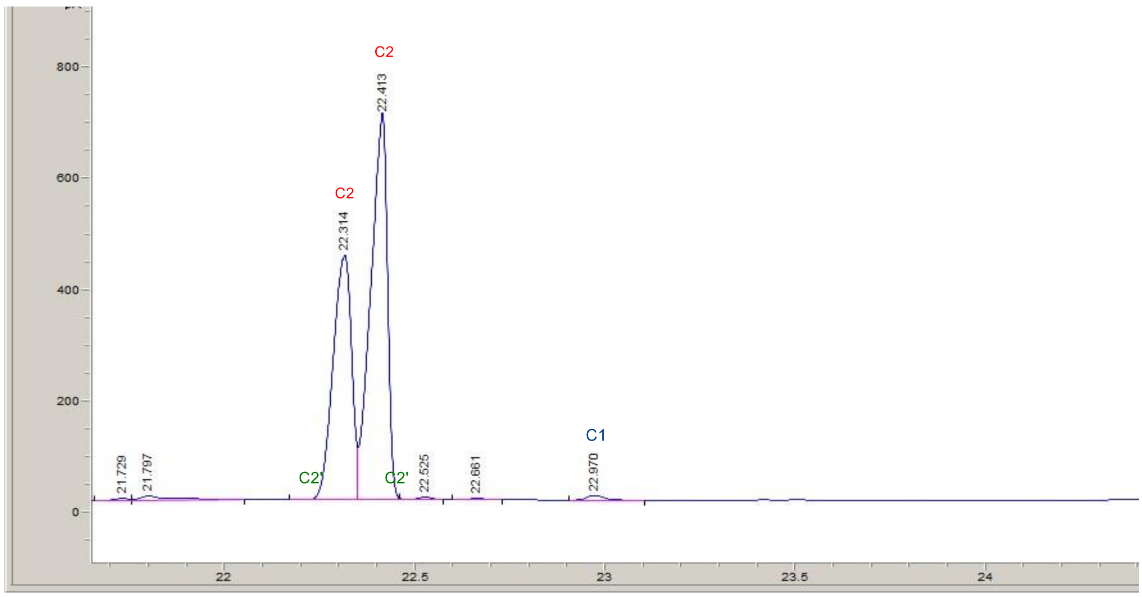

C2
22.413
C2
22.314
C2'
C2'
C1
21.729
21.797
22.525
22.661
22.970

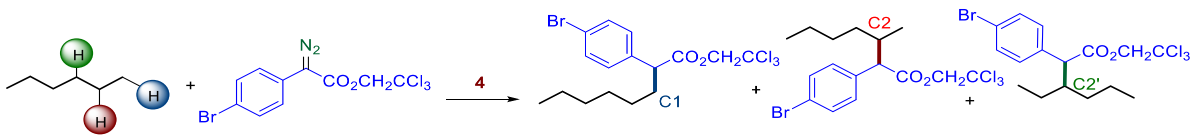
N2
CO2CH2CCl3
Br
4
Br
CO2CH2CCl3
C1
C2
CO2CH2CCl3
Br
Br
CO2CH2CCl3
C2'

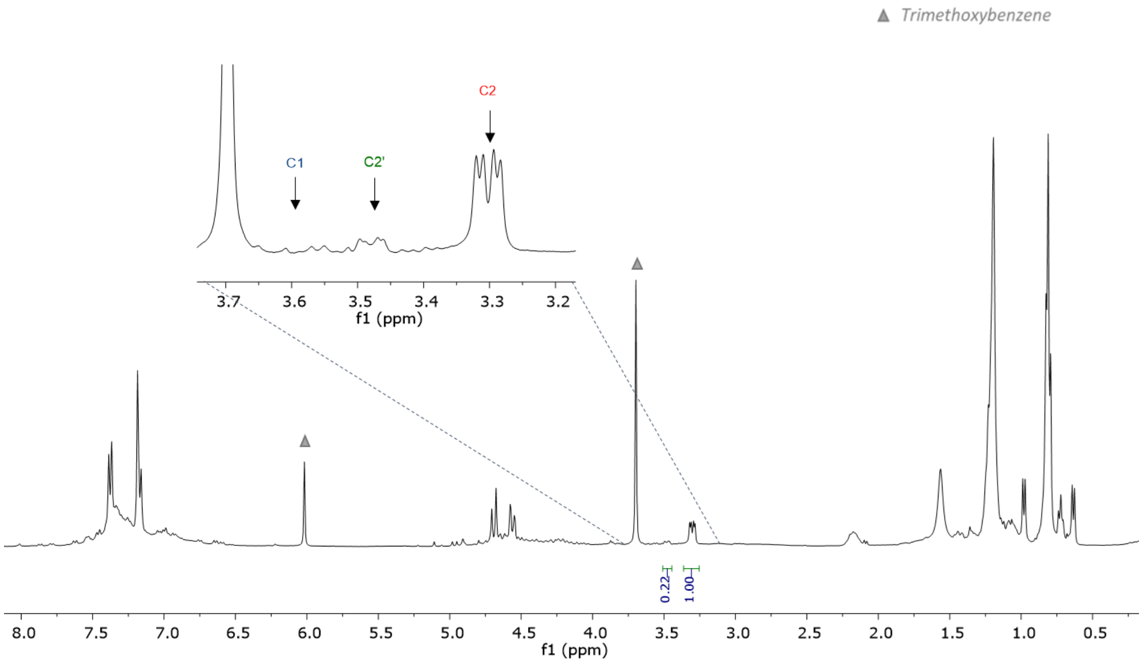
Trimethoxybenzene
C2
C1
C2'
3.7
3.6
3.5
3.4
3.3
3.2
f1 (ppm)
0.22
1.00
8.0
7.5
7.0
6.5
6.0
5.5
5.0
4.5
4.0
3.5
3.0
2.5
2.0
1.5
1.0
0.5
f1 (ppm)

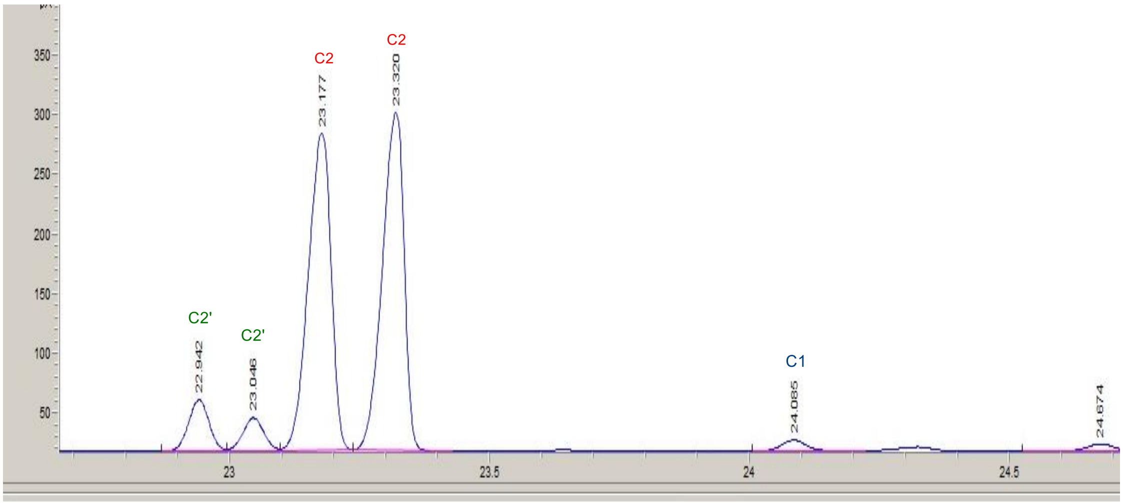
C2'
22.942
C2'
23.046
C2
23.177
C2
23.320
C1
24.085
24.674
350
300
250
200
150
100
50
23
23.5
24
24.5

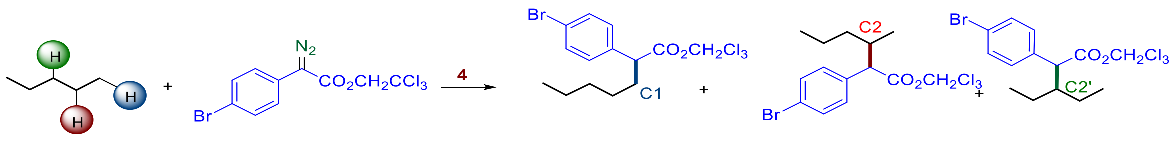

H
H
H
Br
N_2
$CO_2CH_2CCl_3$
4
Br
$CO_2CH_2Cl_3$
C1
C2
$CO_2CH_2Cl_3$
Br
Br
$CO_2CH_2Cl_3$
C2'

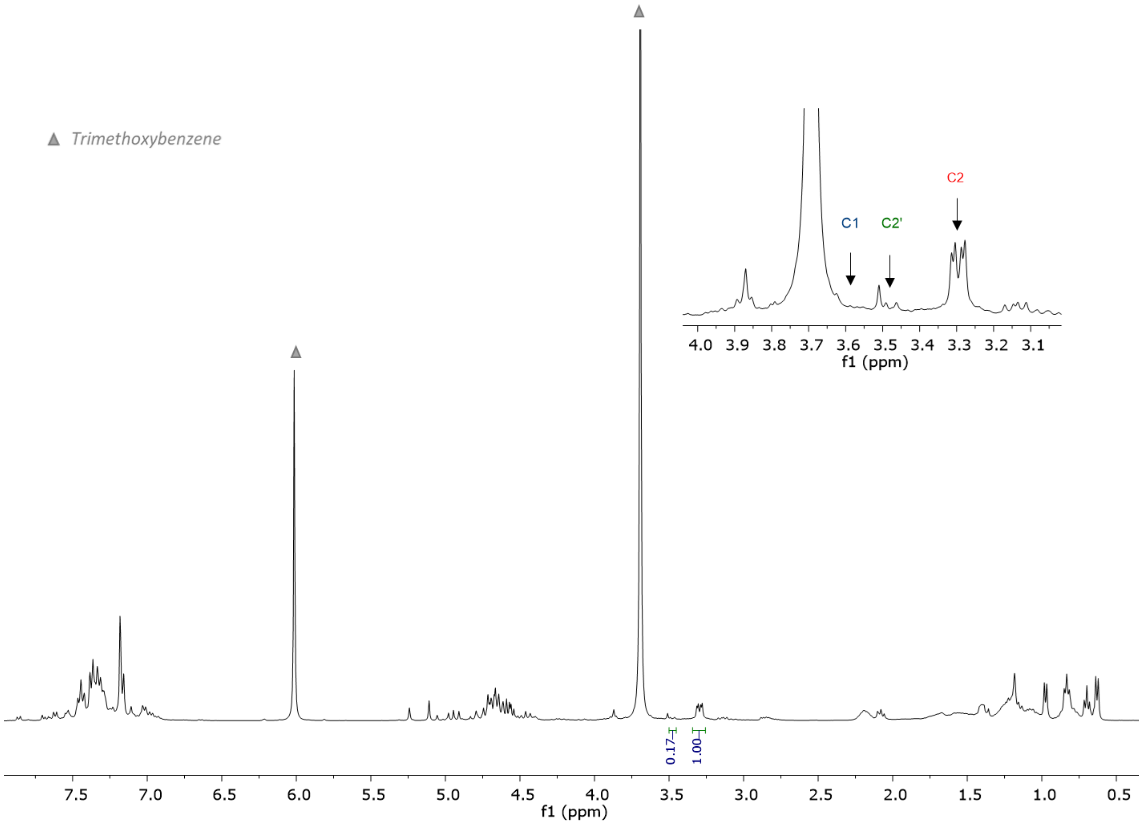

Trimethoxybenzene
C1
C2'
C2
0.17
1.00
f1 (ppm)

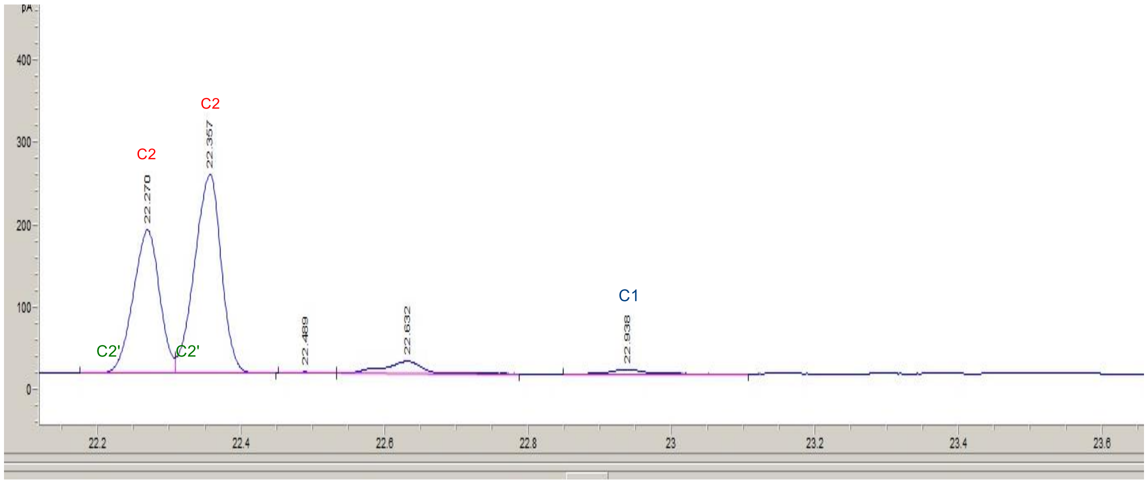

pA
C2
C2
C2'
C2'
C1
22.270
22.357
22.489
22.632
22.938

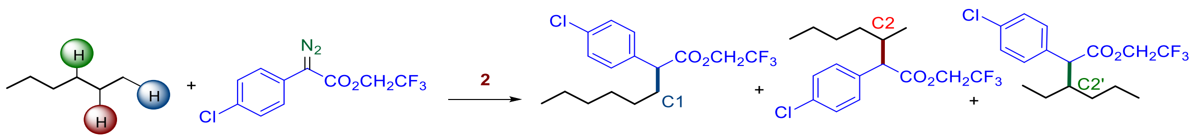
H
H
H
N2
CO2CH2CF3
Cl
2
Cl
CO2CH2CF3
C1
C2
CO2CH2CF3
Cl
Cl
CO2CH2CF3
C2'
+
+
+

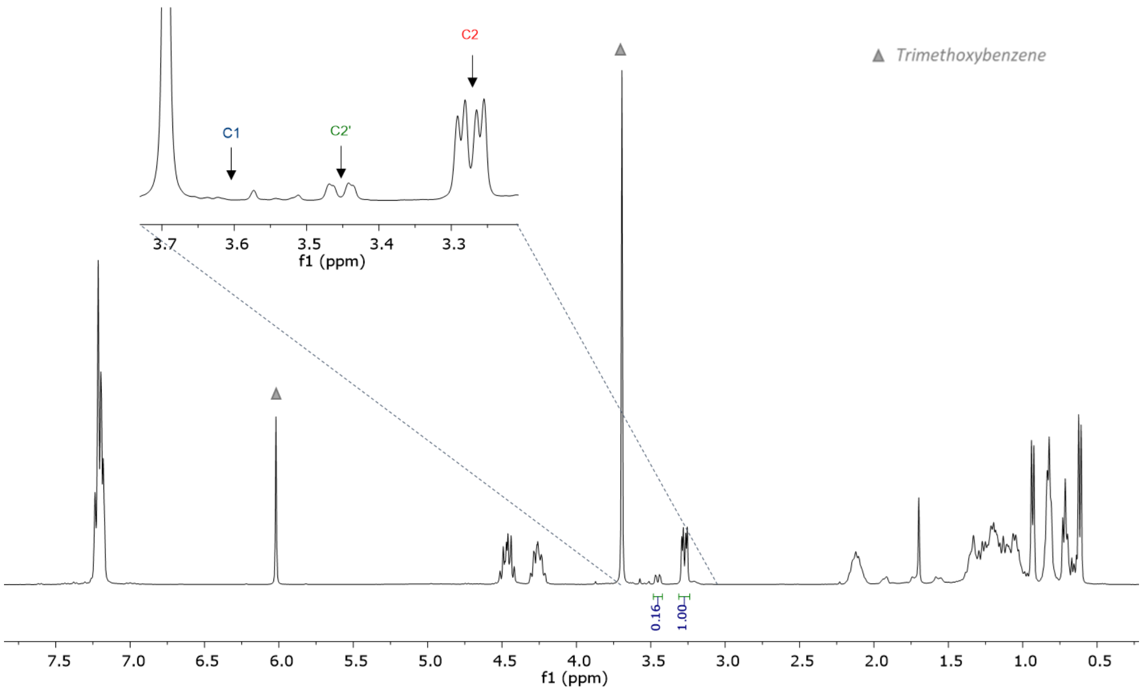
Trimethoxybenzene
C1
C2'
C2
3.7
3.6
3.5
3.4
3.3
f1 (ppm)
0.16
1.00
7.5
7.0
6.5
6.0
5.5
5.0
4.5
4.0
3.5
3.0
2.5
2.0
1.5
1.0
0.5
f1 (ppm)

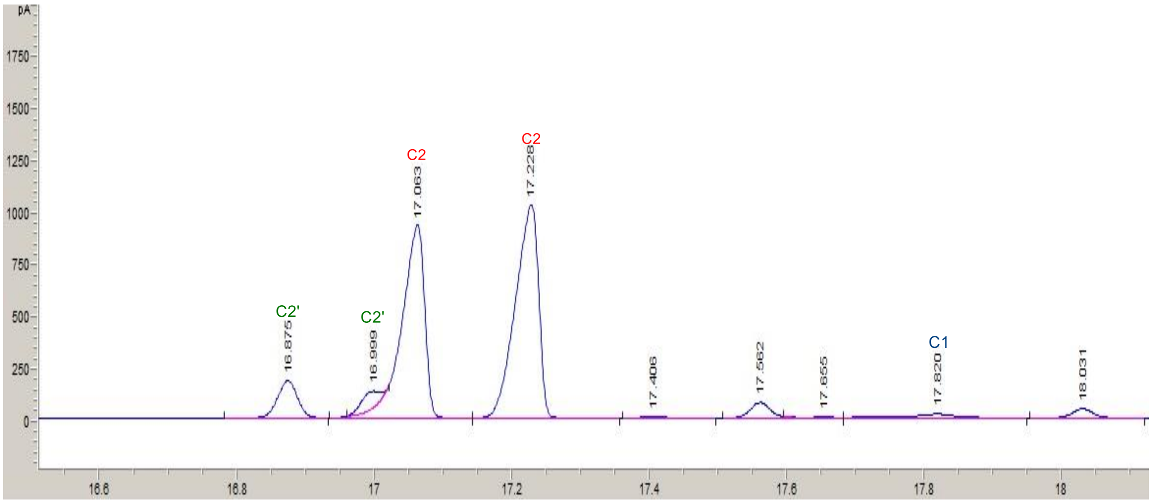
pA
1750
1500
1250
1000
750
500
250
0
C2'
16.875
C2'
16.999
C2
17.063
C2
17.228
17.406
17.562
17.655
C1
17.820
18.031
16.6
16.8
17
17.2
17.4
17.6
17.8
18

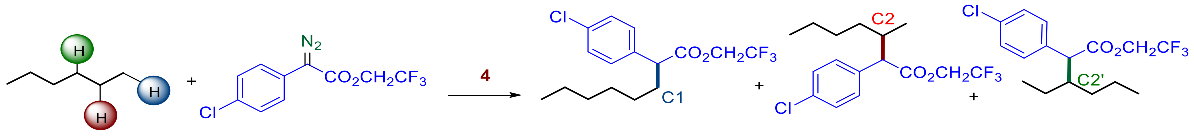
H
H
H
+
N_2
Cl
$CO_2CH_2CF_3$
4
Cl
$CO_2CH_2CF_3$
C1
+
C2
$CO_2CH_2CF_3$
Cl
+
Cl
$CO_2CH_2CF_3$
C2'

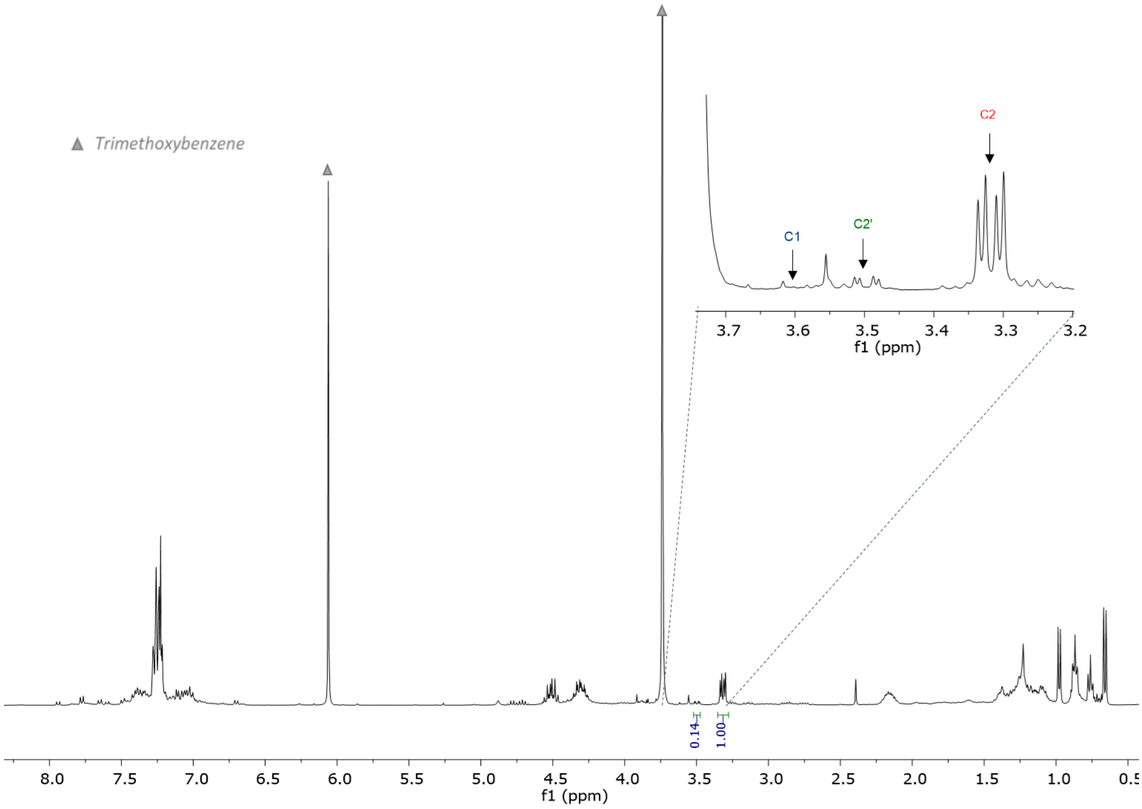
Trimethoxybenzene
C2
C1
C2'
3.7
3.6
3.5
3.4
3.3
3.2
f1 (ppm)
0.14
1.00
8.0
7.5
7.0
6.5
6.0
5.5
5.0
4.5
4.0
3.5
3.0
2.5
2.0
1.5
1.0
0.5
f1 (ppm)

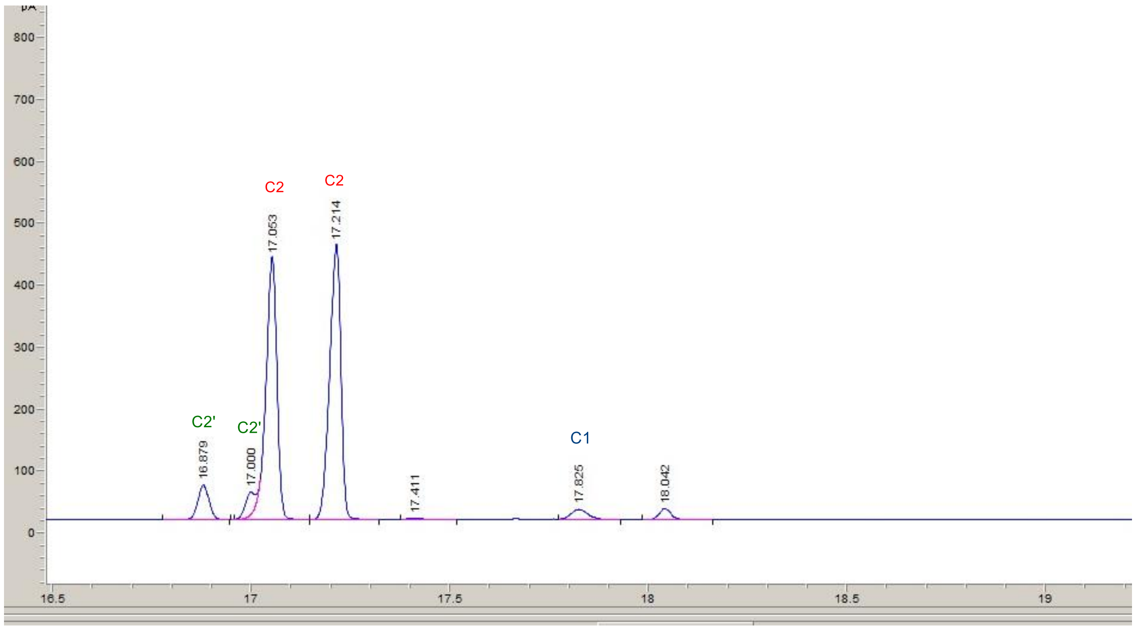
pA
800
700
600
500
400
300
200
100
0
C2
C2
C2'
C2'
C1
16.879
17.000
17.053
17.214
17.411
17.825
18.042
16.5
17
17.5
18
18.5
19

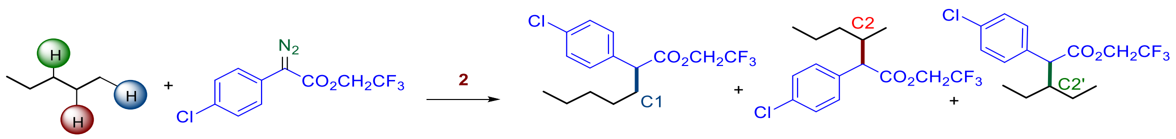
H
H
H
N2
CO2CH2CF3
Cl
2
Cl
CO2CH2CF3
C1
C2
CO2CH2CF3
Cl
Cl
CO2CH2CF3
C2'

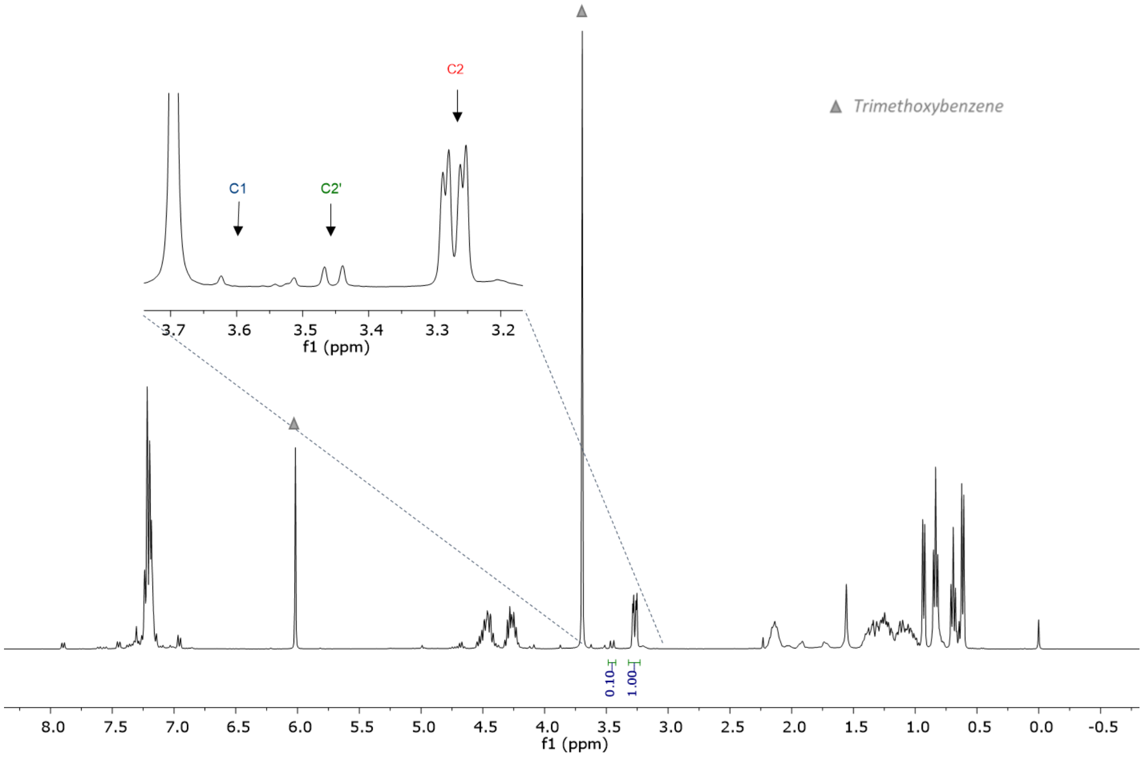
Trimethoxybenzene
C2
C1
C2'
3.7
3.6
3.5
3.4
3.3
3.2
f1 (ppm)
0.10
1.00
8.0
7.5
7.0
6.5
6.0
5.5
5.0
4.5
4.0
3.5
3.0
2.5
2.0
1.5
1.0
0.5
0.0
-0.5
f1 (ppm)

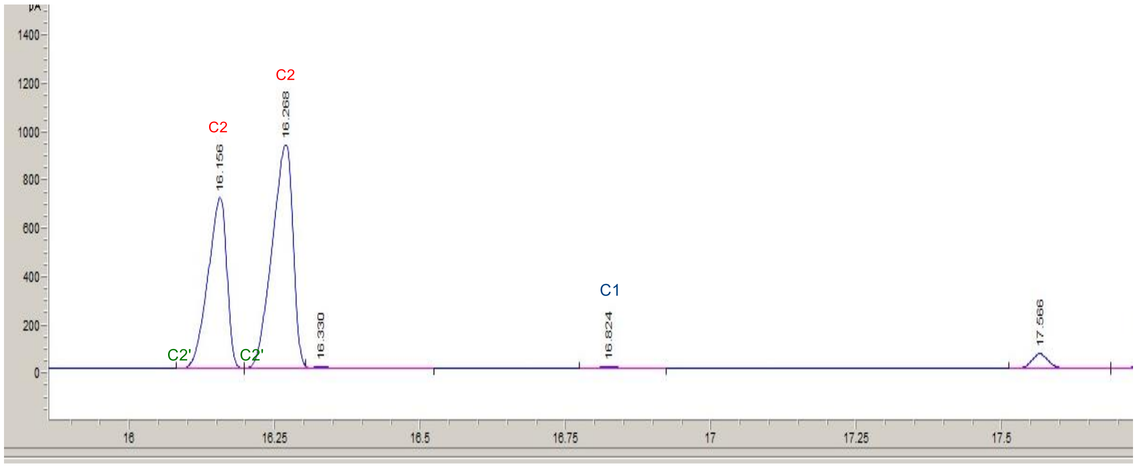
1400
1200
1000
800
600
400
200
0
C2
16.156
C2
16.268
C2'
C2'
16.330
C1
16.824
17.566
16
16.25
16.5
16.75
17
17.25
17.5

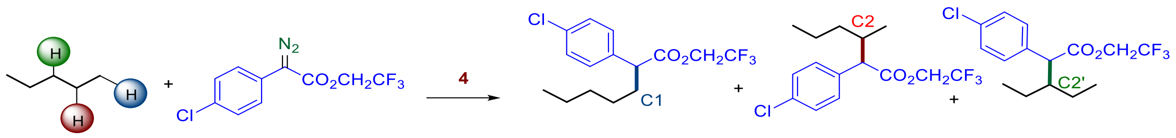
H
H
H
N2
CO2CH2CF3
Cl
4
Cl
CO2CH2CF3
C1
C2
CO2CH2CF3
Cl
Cl
CO2CH2CF3
C2'

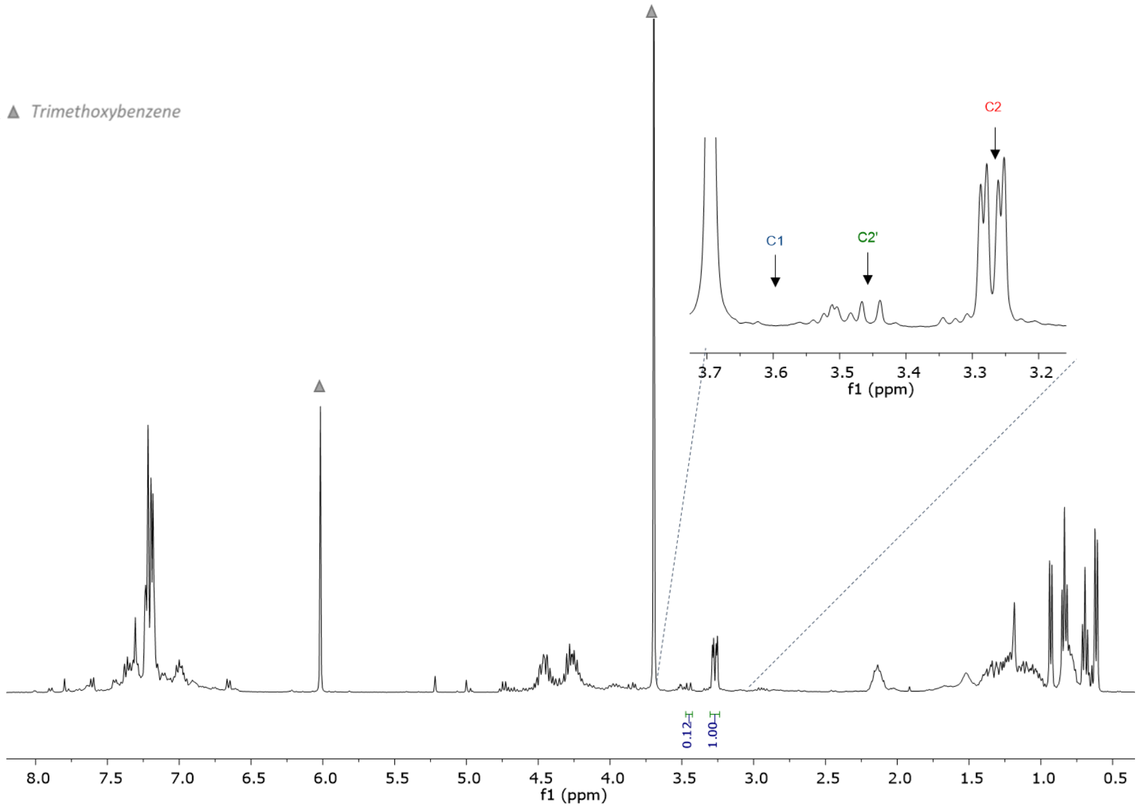
▲ Trimethoxybenzene
C2
C1
C2'
3.7 3.6 3.5 3.4 3.3 3.2
f1 (ppm)
0.12
1.00
8.0 7.5 7.0 6.5 6.0 5.5 5.0 4.5 4.0 3.5 3.0 2.5 2.0 1.5 1.0 0.5
f1 (ppm)

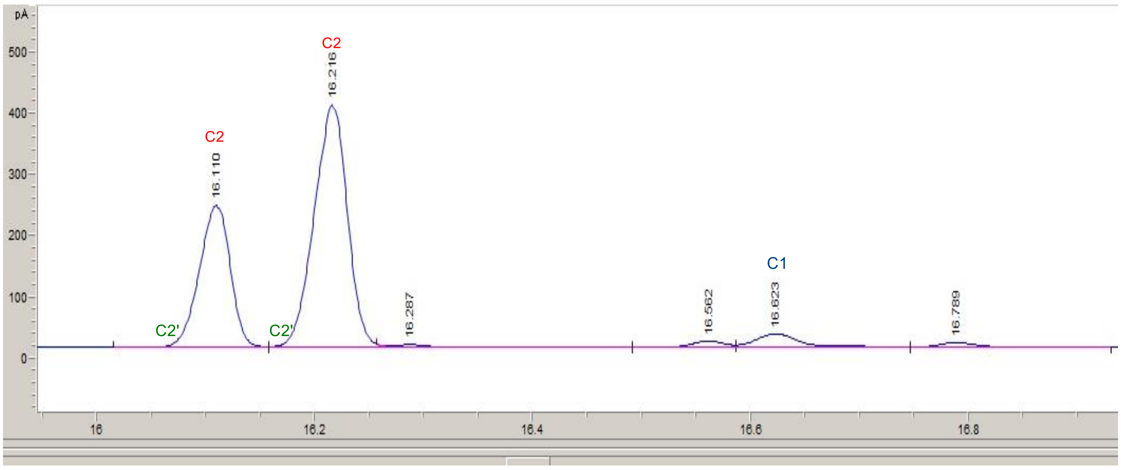
pA
500
400
300
200
100
0
C2
16.110
C2
16.216
16.287
C2'
C2'
16.562
C1
16.623
16.789
16
16.2
16.4
16.6
16.8

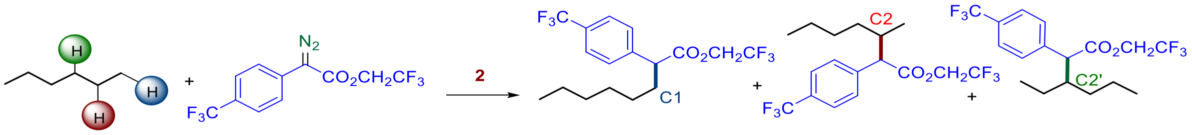
H
H
H
N2
F3C
CO2CH2CF3
2
C1
C2
C2'
+

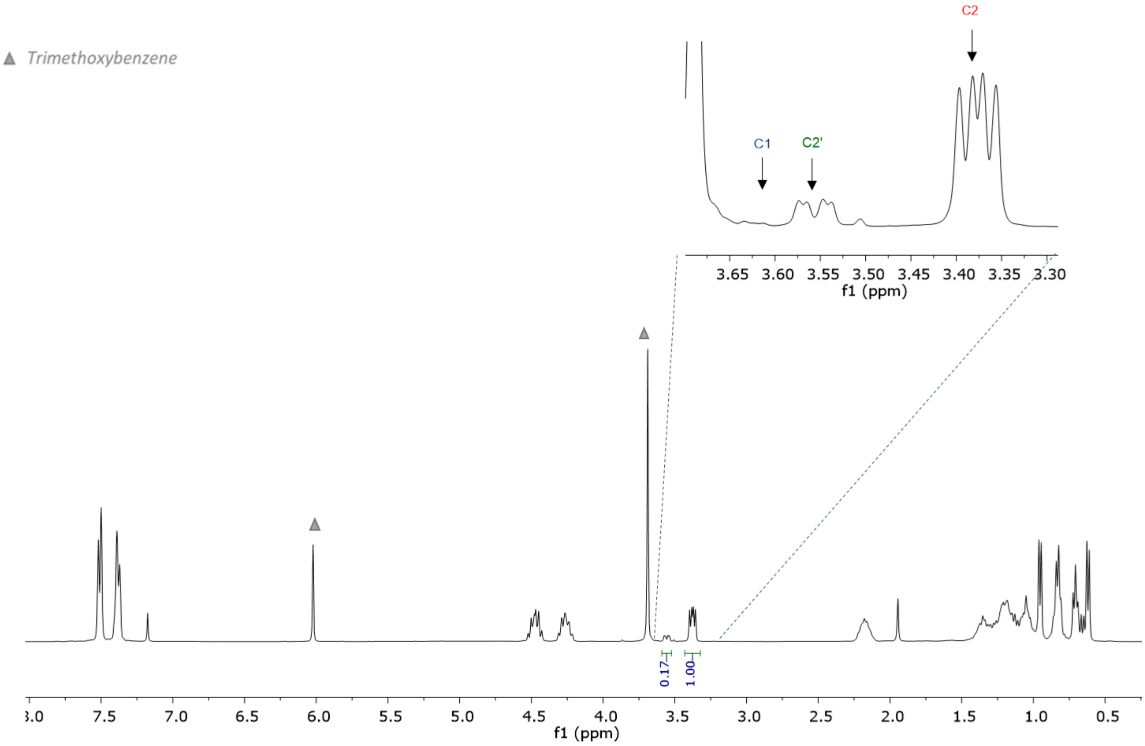
▲ Trimethoxybenzene
C2
C1
C2'
3.65 3.60 3.55 3.50 3.45 3.40 3.35 3.30
f1 (ppm)
0.17
1.00
7.5 7.0 6.5 6.0 5.5 5.0 4.5 4.0 3.5 3.0 2.5 2.0 1.5 1.0 0.5
f1 (ppm)

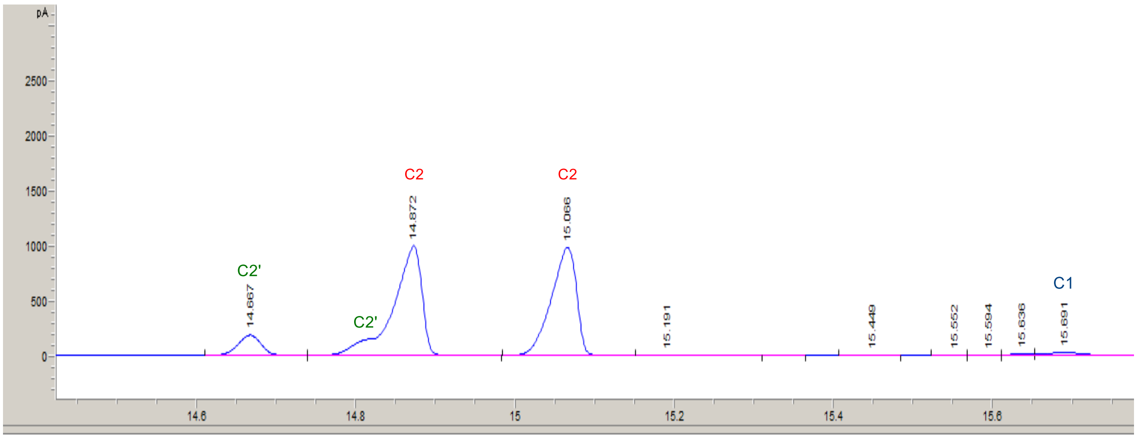
pA
2500
2000
1500
1000
500
0
C2'
14.667
C2'
C2
14.872
C2
15.066
15.191
15.449
15.552
15.594
15.636
C1
15.691
14.6
14.8
15
15.2
15.4
15.6

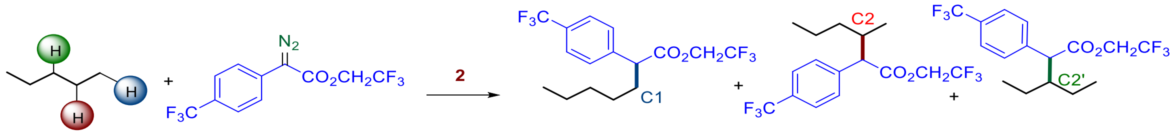
H
H
H
+
N2
F3C
CO2CH2CF3
2
F3C
CO2CH2CF3
C1
+
C2
CO2CH2CF3
F3C
F3C
CO2CH2CF3
+
C2'

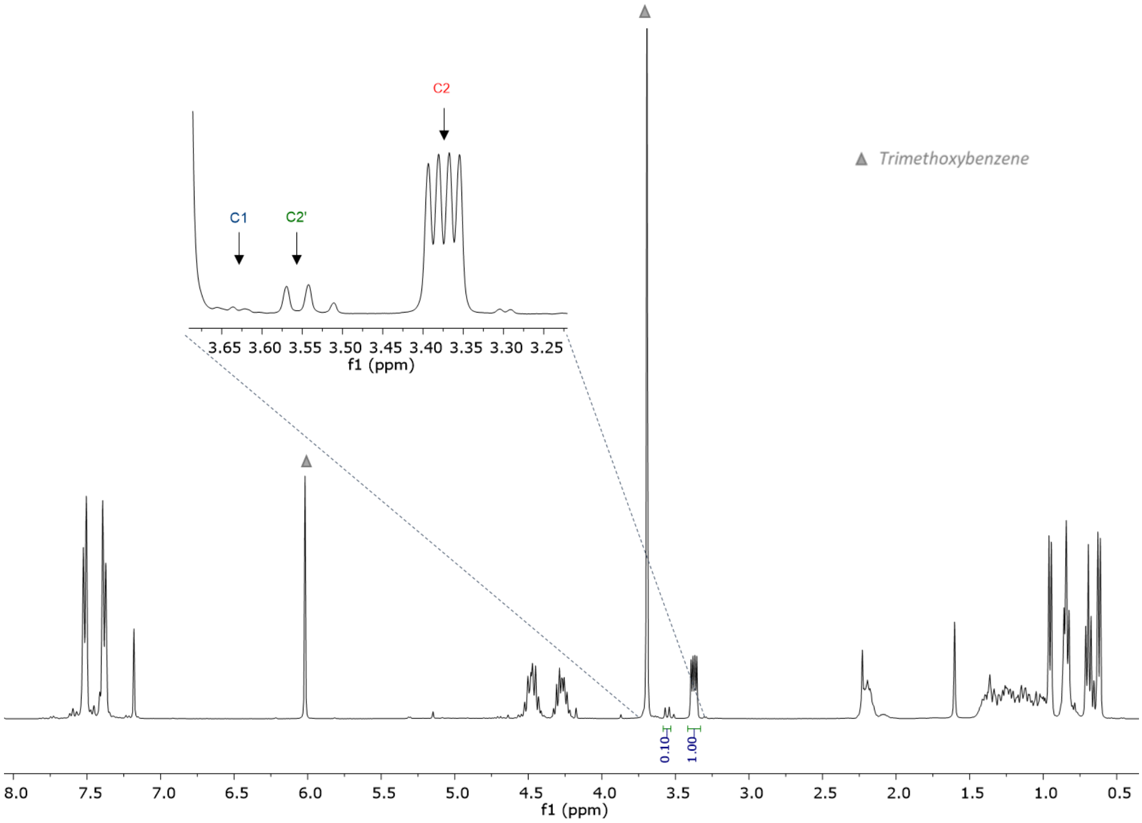
C2
C1
C2'
3.65 3.60 3.55 3.50 3.45 3.40 3.35 3.30 3.25
f1 (ppm)
Trimethoxybenzene
0.10
1.00
8.0 7.5 7.0 6.5 6.0 5.5 5.0 4.5 4.0 3.5 3.0 2.5 2.0 1.5 1.0 0.5
f1 (ppm)

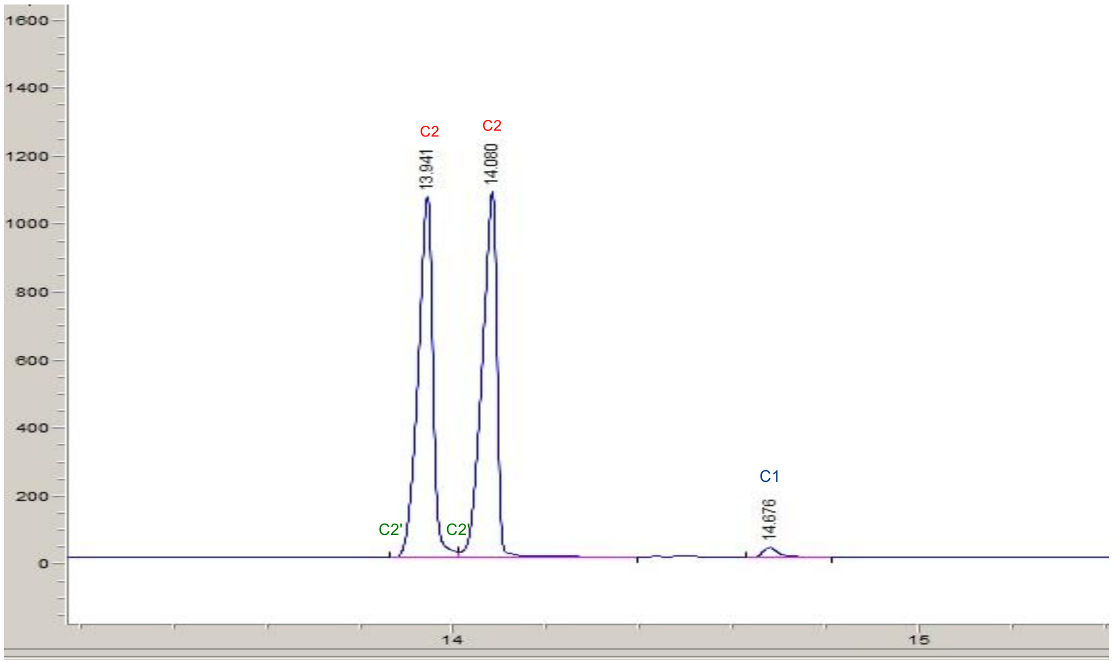
1600
1400
1200
1000
800
600
400
200
0
C2
13.941
C2
14.080
C1
14.676
C2'
C2'
14
15

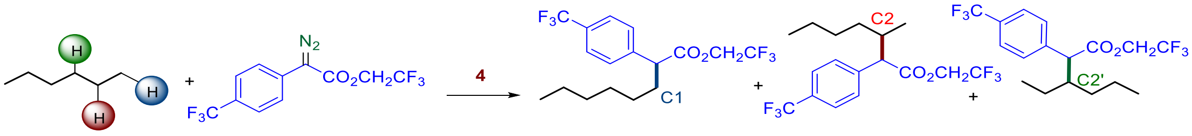
H
H
H
N2
CO2CH2CF3
F3C
4
F3C
CO2CH2CF3
C1
C2
CO2CH2CF3
F3C
F3C
CO2CH2CF3
C2'

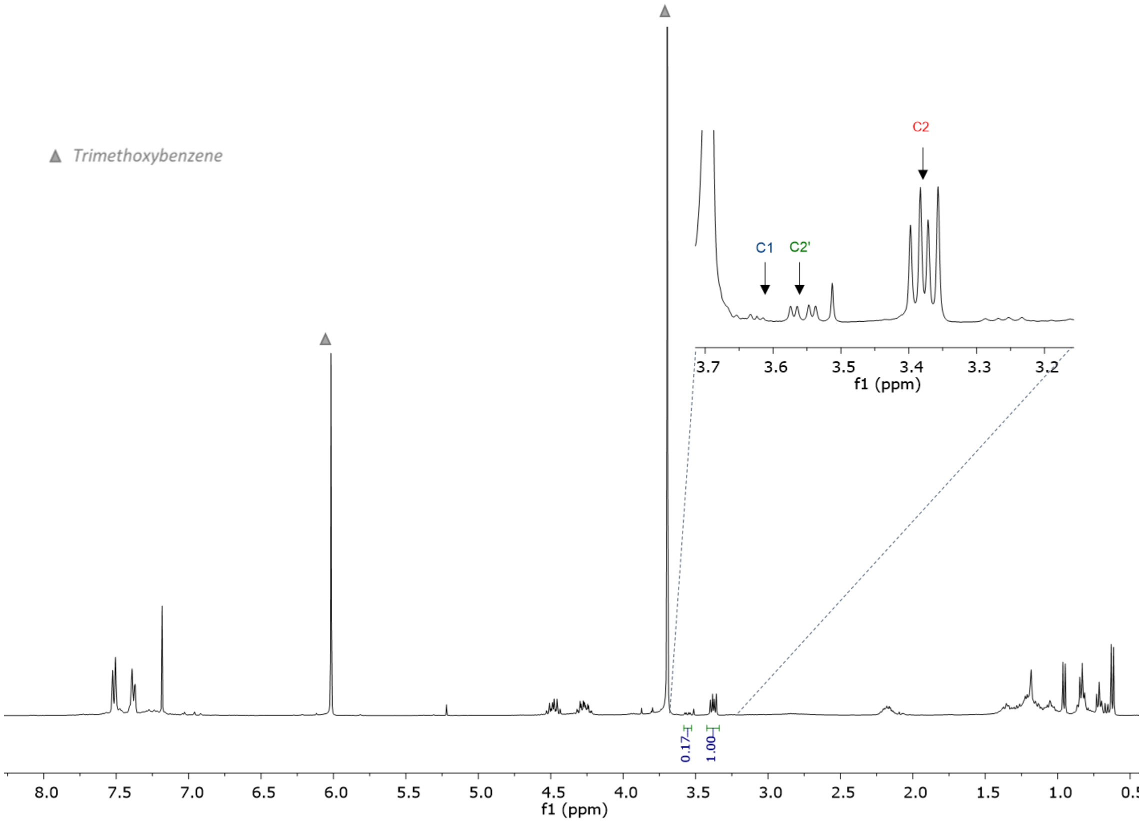
Trimethoxybenzene
C2
C1
C2'
3.7
3.6
3.5
3.4
3.3
3.2
f1 (ppm)
0.17
1.00
8.0
7.5
7.0
6.5
6.0
5.5
5.0
4.5
4.0
3.5
3.0
2.5
2.0
1.5
1.0
f1 (ppm)

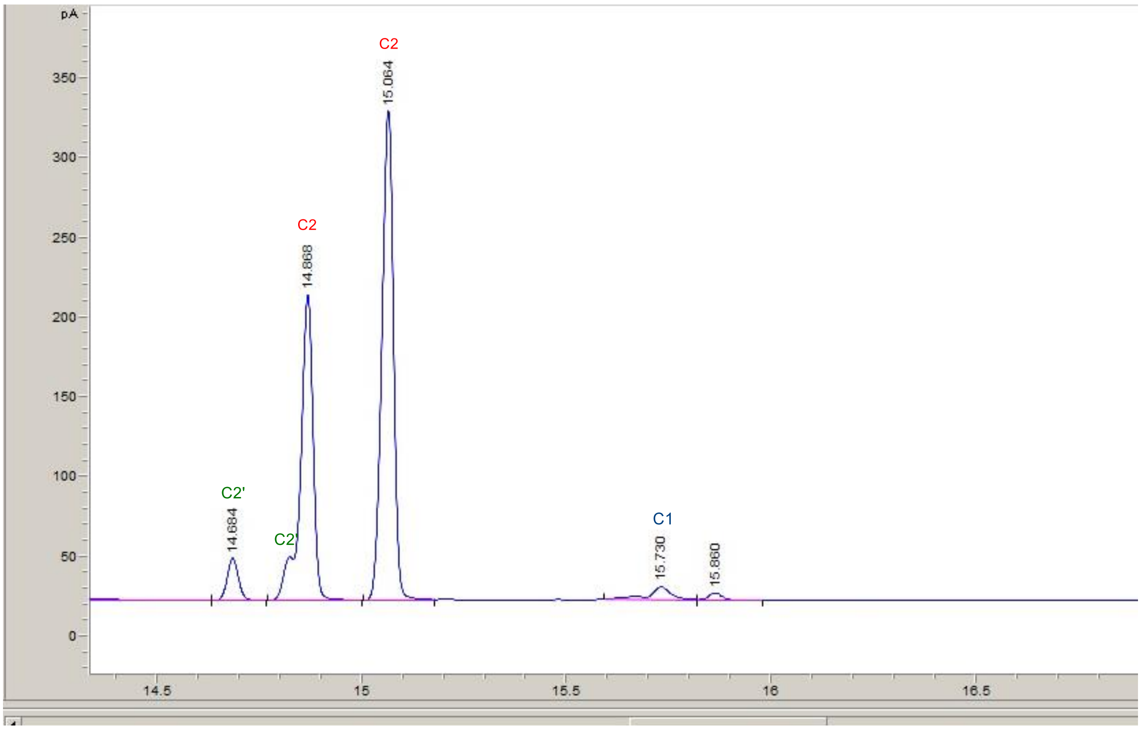
pA
350
300
250
200
150
100
50
0
C2
15.064
C2
14.868
C2'
14.684
C2'
C1
15.730
15.860
14.5
15
15.5
16
16.5

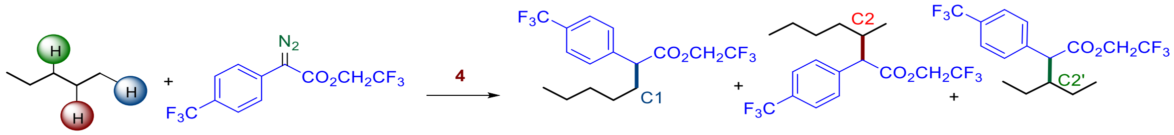
H
H
H
+
N2
CO2CH2CF3
F3C
4
F3C
CO2CH2CF3
C1
+
C2
CO2CH2CF3
F3C
+
F3C
CO2CH2CF3
C2'

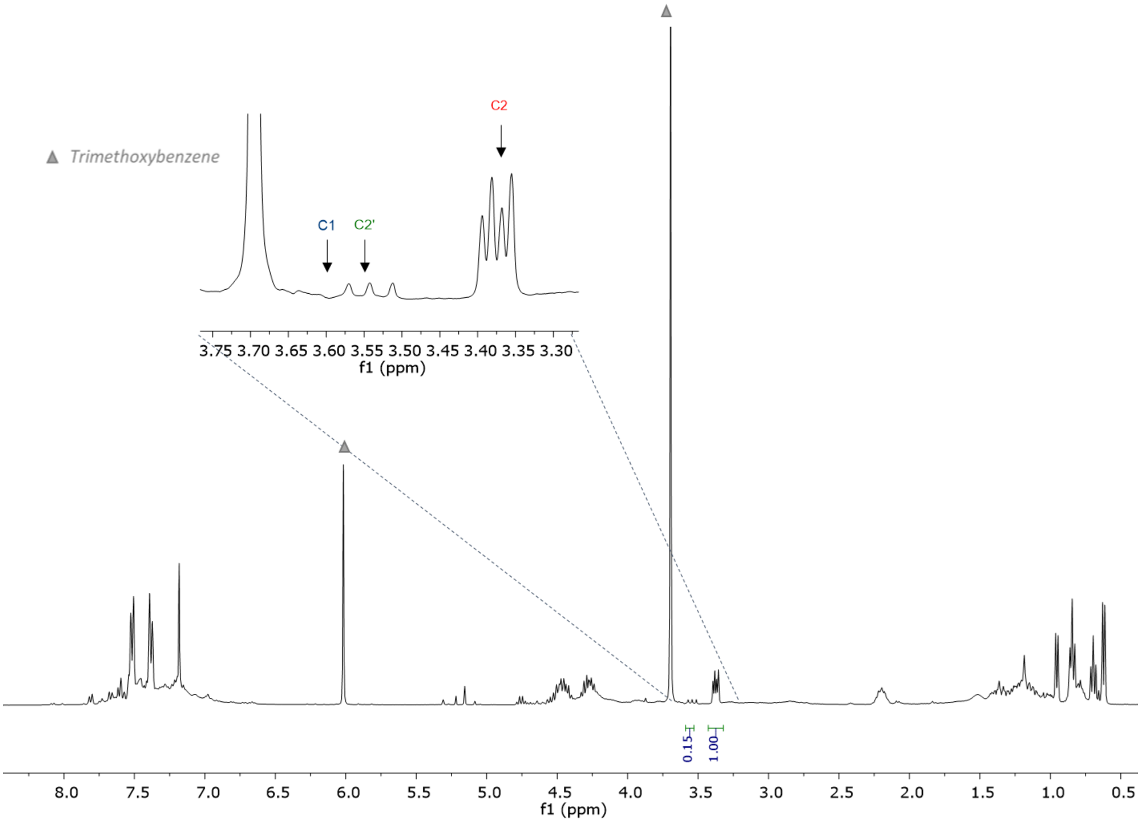

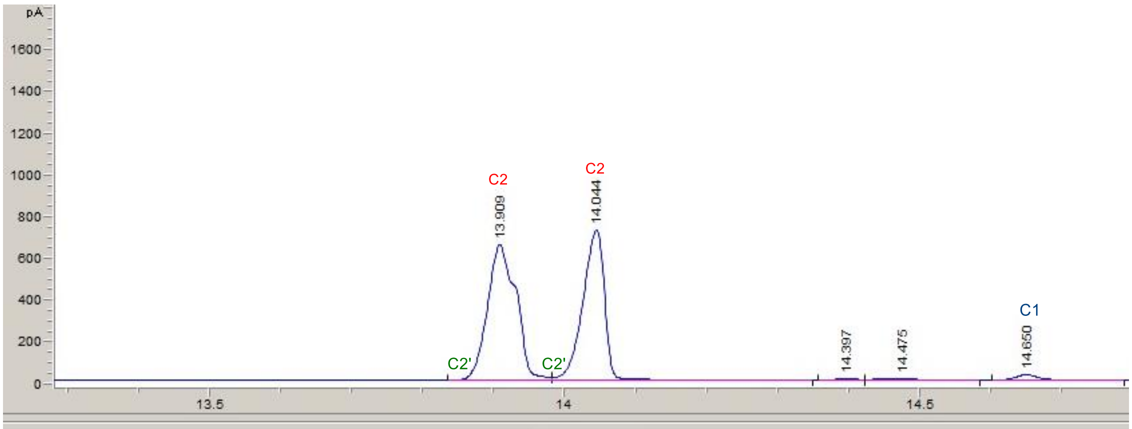

__Reaction employing diethyl diazomalonate__.

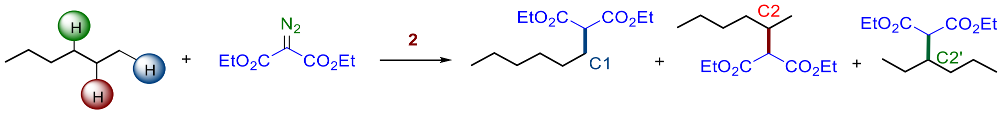

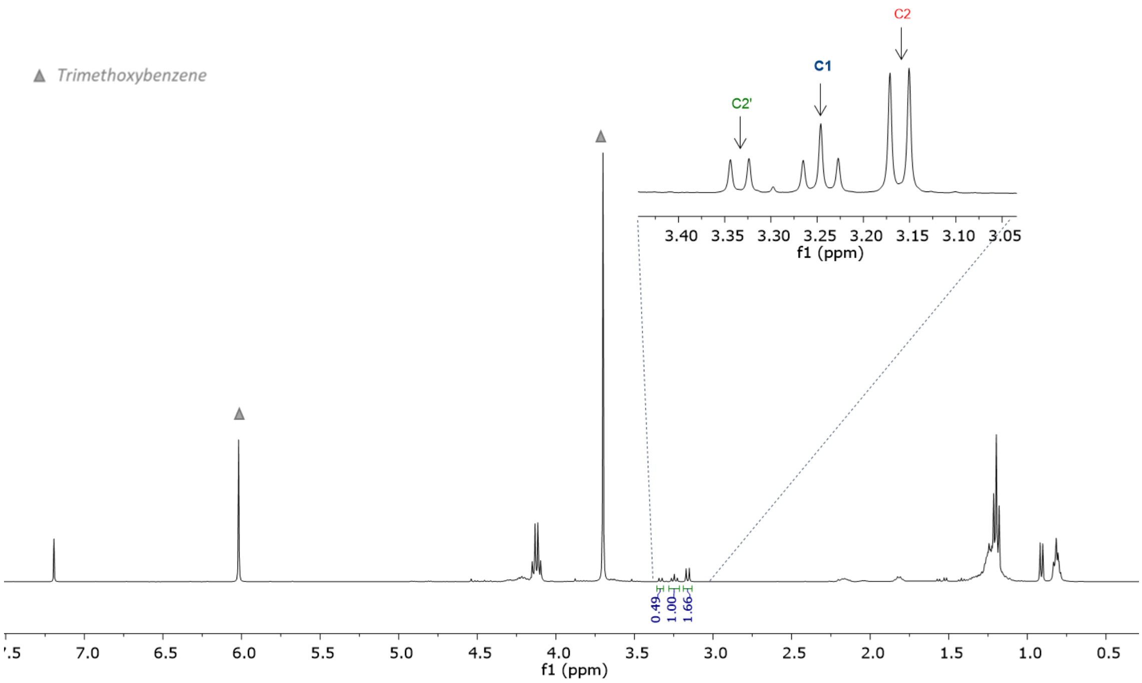

Trimethoxybenzene
C2
C1
C2'
3.40 3.35 3.30 3.25 3.20 3.15 3.10 3.05
f1 (ppm)
0.49
1.00
1.66
7.5 7.0 6.5 6.0 5.5 5.0 4.5 4.0 3.5 3.0 2.5 2.0 1.5 1.0 0.5
f1 (ppm)

9. References

[1] Álvarez, M.; Molina, F.; Fructos, M. R.; Urbano, J.; Álvarez, E.; Sodupe, M.; Lledós, A.; Pérez, P. J. *Dalton Trans.* **2020**, *49*, 14647-14655.

[2] Díaz-Requejo, M. M.; Belderraín, T. R.; Nicasio, M. C.; Trofimenko, S.; Pérez, P. J. *J. Am. Chem. Soc.* **2003**, *125*, 12078–12079.

[3] Gava, R.; Olmos, A.; Noverges, B.; Varea, T.; Álvarez, E.; Belderrain, T. R.; Caballero, A.; Asensio, G.; Pérez, P. J. *ACS Catal.* **2015**, *5*, 3726-3730.

[4] (a) Hari, D. P.; Waser, J. *J. Am. Chem. Soc.* **2017**, *139,* 8420-8423; (b) Bess, E. N.; Guptill, D. M.; Davies, H. M. L. ; Sigman, M. S. *Chem. Sci*. **2015**, *6*, 3057-3062. (c) Liao, K.; Negretti, S.: Musaev, D. G.; Bacsa, J.; Davies, H. M. L. *Nature* **2016**, *533*, 230-234. (d) Sar, S.; Guha, G.; Prabakar, T.; Maiti, D.; Sen, S. *J. Org. Chem.* **2021,** *86*, 11736-11747.

[5] La, M. T.; Kim, H. K. *Tetrahedron* **2018**, *74*, 3748-3754.

[6] Talukdar, A.; Ganguly, D.; Roy, S.; Das, N.; Sarkar, D. *J. Med. Chem*. **2021**, *64*, 8010-8041.

[7] de Quadras, L.; Stahl, J.; Zhuravlev, F.; Gladysz, J. A. *J. Organomet. Chem.* **2007**, *692*, 1859-1870.

[8] Kolb, N.; Hofsäß, R.; Meier, M. A. *Eur. J. Lipid. Sci. Technol*. **2013**, *115*, 729-734.

[9] Tayama, E.; Isaka, W. *Org. Lett.* **2006**, *8*, 5437-5439.
